# A-eye: Automated 3D MRI segmentation and morphometric feature extraction for eye and orbit atlas construction

**DOI:** 10.1371/journal.pone.0352257

**Published:** 2026-07-02

**Authors:** Jaime Barranco, Adrian Konstantin Luyken, Yiwei Jia, Hamza Kebiri, Philipp Stachs, Pedro M. Gordaliza, Oscar Esteban, Yasser Aleman, Raphael Sznitman, Felix Streckenbach, Oliver Stachs, Sönke Langner, Benedetta Franceschiello, Meritxell Bach Cuadra

**Affiliations:** 1 CIBM Center for Biomedical Imaging, Lausanne, Switzerland; 2 Department of Radiology, Lausanne University Hospital (CHUV) and University of Lausanne (UNIL), Lausanne, Switzerland; 3 HES-SO University of Applied Sciences and Arts Western, Switzerland; 4 The Sense Innovation and Research Center, Lausanne and Sion, Switzerland; 5 Department of Ophthalmology, Rostock University Medical Center, Rostock, Germany; 6 School for Cellular and Biomedical Sciences, University of Bern, Bern, Switzerland; 7 Karlsruhe Institute of Technology (KIT), Karlsruhe, Germany; 8 ARTORG Center for Biomedical Engineering, University of Bern, Bern, Switzerland; 9 Department Life, Light & Matter, University of Rostock, Rostock, Germany; 10 Institute for Diagnostic and Interventional Radiology, Pediatric and Neuroradiology, Rostock University Medical Center, Rostock, Germany; University of Marburg: Philipps-Universitat Marburg, GERMANY

## Abstract

In this study we introduce automated 3D segmentation of the healthy human adult eye and orbit from Magnetic Resonance Images, to improve ophthalmic diagnostics and treatments. Past efforts have primarily focused on small sample sizes and varied imaging modalities. Here, we leverage a large-scale dataset of T1-weighted MRI of 1245 subjects and the deep learning-based nnU-Net for MR-Eye segmentation tasks. The results showcase robust and accurate 3D segmentation of lens, globe, optic nerve, rectus muscles, and orbital fat. We also present the automated estimation of key ophthalmic morphometry biomarkers such as axial length and volumetry, while benchmarking correlations between body mass index and eye structure volumes. Quality control protocols are introduced through the pipeline to ensure the reliability of the segmented large-scale data, further enhancing the applicability of our algorithm in clinical research. As a major outcome we provide the first large-scale unbiased eye atlases (female, male, and combined) towards standardization of spatial normalization tools for MR-Eye.

## Introduction

According to the World Health Organization (WHO), 2.2 billion people have vision impairment or blindness [1] and preventable causes account for 80% of the total global visual impairment burden. The eyes, small, complex, and delicate structures that serve as our primary sensory organ [2], are primarily imaged via funduscopy [3], ultrasound [4], and optical computed tomography (OCT) [5,6]. Such devices can extract anatomical measurements of the eyes, but fail to image the posterior part of the eye, therefore providing partial information in presence of volumetric lesions, calcifications or other pathologies [7–10]. In such clinical scenarios, Magnetic Resonance Imaging (MRI), with its non-invasive nature and penetration capabilities, provides 3D measurements of the complete eye, related to both the tissue and organ structure, and informs about particle deposits within the tissues, such as calcifications or tissue deformations. Ophthalmic MRI [7–10], known as MR-Eye [11–15], has proven highly effective in oncology, for the evaluation and treatment planning of tumors, as well as for the quantification of orbital inflammation and for refractive surgery planning [10]. Furthermore, given that neurodegenerative disorders frequently involve ocular and visual comorbidities [11,16,17], and oculomotor dysfunctions can signify underlying brain injuries [18,19], advancing the current capabilities of MR-Eye-based analyses is paramount.

Manual segmentation has traditionally been the reference standard for delineating ocular and orbital structures and tumors [10,20], but it is labor-intensive, operator-dependent, and not scalable for large studies. Fast and reliable clinical analysis therefore requires fully automated, robust segmentation algorithms. Early semi-automated methods used parametric shape-based models with spheres and ellipsoids [20–22]. Active shape models using machine learning added flexibility for anatomical variability by enabling data-driven deformations [23,24]. More recently, deep learning approaches, primarily 2D and 3D U-Nets, have been used to fully automate the segmentation [25–30], with some hybrid models and clustering techniques [28,29,31–34]. Yet, these efforts largely focus on a limited set of ocular structures (e.g., lens, vitreous humor (VH), sclera, cornea), with rare inclusion of the optic nerve [24,26] and minimal attention to key orbital components like rectus muscles (RM) or orbital fat (i.e., intraconal and extraconal), therefore limiting the development of a comprehensive eye-orbit model. Some studies include the segmentation of tumors, usually retinoblastoma or uveal melanoma [24,27,31,32], while our study focuses on healthy adult eye and orbit structures. Moreover, most studies used small datasets (typically 24–4 annotated subjects) and often relied on multi-contrast MRI, restricting generalizability. Importantly, despite the known influence of image quality on automated neuroimaging analyses [35–39], quality control is rarely integrated into MR-Eye pipelines, with only a few exceptions [24,32]39.

Yet, MR-Eye is progressively advancing towards a deeper understanding and early interception of ophthalmic diseases. Several key ophthalmic morphometry biomarkers—such as axial length (AL) [40–42], which is relevant in refractive errors, myopia, hyperopia, glaucoma, and retinal detachment, and volumetric measurements [43–46], which are valuable in assessing eye growth abnormalities, glaucoma, macular degeneration, and orbital tumors—can be extracted from MR images. However, such extractions are performed manually and remain time-consuming for clinicians. While the automated extraction of such biomarkers from MR-Eye could benefit clinics and research in terms of time and performance, no existing tools support automated estimation of AL (apart from [47], in Japanese) nor volumetric values from 3D MRI. Current volumetric studies are in fact limited. In [43,44], the authors reported only the total orbital volume (~27.5 cm³), while [45] analyzed the orbital muscle fraction relative to total orbital volume in patients with Graves’ orbitopathy, also known as endocrine orbitopathy, with volumetry provided only for a single example case. In [46], two radiologists manually measured the anterior chamber (between the cornea and iris) and the whole eyeball (globe, lens, and anterior chamber combined), calculating volumes by tracing freehand contours and summing areas across slices multiplied by section thickness. Despite these manual efforts, there remains no ophthalmic technology, even beyond MR-Eye, capable of delivering volumetric estimations of the eye and its substructures at millimeter resolution.

Furthermore, to further enhance the usability of MR-Eye-based assessment, an eye atlas is needed. Such a tool enables spatial navigation, colocalization, and the quantitative analysis of eye morphology. In other branches, atlases could serve as essential spatial references, supporting the interpretation of anatomical variability and facilitating consistent measurements across populations. In neuroimaging, for example, anatomical and probabilistic atlases have long been foundational [48–51], providing standardized templates for spatial normalization and cross-subject analysis, and enabling investigations into structural brain variation, function, and pathology. Yet, comparable tools are notably absent in ophthalmic imaging. A recent study [52] has initiated the creation of an unbiased MRI-based eye atlas, made available through the HuBMAP project [53], using a sample of 100 images across multiple MRI contrasts: T1-weighted (T1w) pre- and post-contrast, T2w TSE, and T2w FLAIR). While this represents a significant first step forward, there remains the need for a large-scale, population-representative, eye atlas capable of including sex-specific versions. This need is particularly pressing given the growing evidence that sex differences influence disease presentation and progression in ocular conditions such as endocrine orbitopathy [54–57].

The contributions of this work are three-fold. First, we present a comprehensive and accurate 3D segmentation framework [58] for healthy adult human eye and orbit structures—including the lens, globe, optic nerve, rectus muscles, and orbital fat—using T1w MRI data from 1,245 healthy subjects. Second, building on this framework, we enable automated large-scale extraction of key ocular biomarkers, namely axial length and structure-specific volumetry, across the full cohort. Third, we provide the first unbiased, large-scale T1w MR-Eye atlases—stratified by sex (594 males, 616 females) and combined (1,210 subjects)—with detailed labels of eye and orbital anatomy. These atlases are made publicly available [59] in standard volumetric coordinate spaces (VCS) [50,51], offering a foundational spatial reference for MR-Eye research and clinical applications. In support of these contributions, we also introduce a dedicated MR-Eye quality control (QC) protocol tailored to ocular imaging, overcoming the limitations of existing brain QC tools.

## Results

Our work presents the automated 3D segmentation of eye and orbital structures, along with automated extraction of key morphometry biomarkers such as AL and volumetric measurements. Leveraging the extensive scale of our database, we introduce a large-scale atlas of the eye in MRI (N>>100).

### Automated segmentation

**Fig 1:**
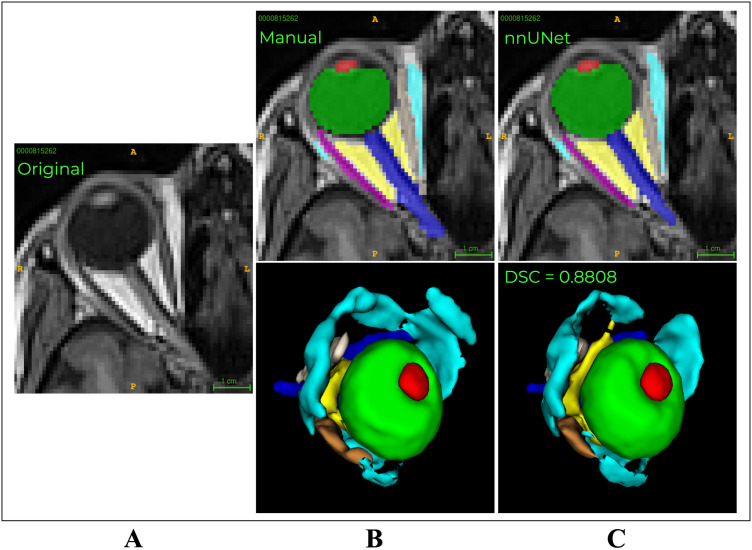
Visual comparison of manual and automated segmentation. (A) Original T1w image. (B) Manual segmentation on 9 ROI: lens (red), globe (green), optic nerve (dark blue), intraconal fat (yellow), extraconal fat (cyan), lateral rectus muscle (magenta), medial rectus muscle (ivory), inferior rectus muscle (blue), and superior rectus muscle (brown). (C) nnU-Net segmentation. We provide preliminary overall DSC (averaged across all structures) for nnU-Net compared to the manual segmentation (ground truth).

Fig 1 displays a visual representation of the obtained segmentation. To quantitatively assess the performance of our algorithm in delineating the eye structures anatomically as compared to manual expert segmentation (referred to as ground truth, or more correctly as surrogate truth or reference standard), we used a set of complementary similarity metrics (Dice score – DSC, Hausdorff distance – HD, and volume difference – VD) on a test set of 43 subjects. These 43 subjects (age 38–77, 28 females and 15 males) had non-excluded MR-Eye image quality, i.e., rating scores above 1; the images did not contain major classic artefacts, as rated by MR-Eye experts (see Materials and Methods section).

**Fig 2:**
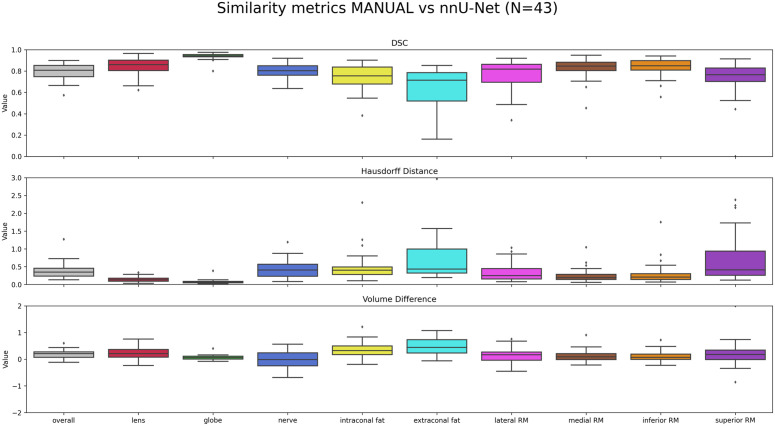
Similarity metrics on 43 subjects. On the y-axis, the similarity metrics’ scale (three plots, from top to bottom DSC, HD, VD), and on the x-axis, the different eye structures.

We show that the proposed model produces accurate results in delineating all eye structures (average score across structures: DSC = 0.80 ± 0.07, HD = 0.37 ± 0.20 mm, and VD = 0.18 ± 0.14mm3) as compared to the ground truth (scores detailed in Fig 2 and in Table 1). As expected, lower performance was encountered in more anatomically variable structures (fat, superior RM). Consistent with these metrics, in S1 Fig, we observe strong relationships between them across all regions. DSC is negatively correlated with HD and VD, indicating that higher overlap corresponds to better contour and volume agreement, while HD and VD are positively correlated, showing that larger boundary errors tend to be associated with larger volume differences. Weaker correlations are found in the optic nerve and rectus muscles, probably due to their variable shape across subjects. All correlations are statistically significant (p < 0.05).

**Table 1:** Descriptive statistics of similarity metrics per structure on N = 43 subjects. For each metric, both the mean ± standard deviation (with 95% confidence interval) and the median [interquartile range] (with 95% confidence interval from bootstrap resampling) are reported.

Structure	DSC	HD	VD
Average	0.80 ± 0.07 (CI: [0.78–0.82]), 0.81 [0.75–0.85] (CI: [0.76–0.83])	0.37 ± 0.20 (CI: [0.31–0.43]), 0.35 [0.23–0.44] (CI: [0.30–0.40])	0.18 ± 0.14 (CI: [0.14–0.22]), 0.19 [0.07–0.27] (CI: [0.12–0.24])
Lens	0.84 ± 0.09 (CI: [0.81–0.87]), 0.86 [0.80–0.90] (CI: [0.84–0.89])	0.14 ± 0.07 (CI: [0.12–0.16]), 0.13 [0.09–0.18] (CI: [0.11–0.15])	0.24 ± 0.23 (CI: [0.17–0.31]), 0.20 [0.07–0.35] (CI: [0.15–0.29])
Globe	0.94 ± 0.03 (CI: [0.93–0.95]), 0.94 [0.93–0.95] (CI: [0.94–0.95])	0.07 ± 0.05 (CI: [0.05–0.09]), 0.07 [0.05–0.08] (CI: [0.05–0.08])	0.06 ± 0.08 (CI: [0.04–0.08]), 0.06 [0.01–0.10] (CI: [0.02–0.08])
Optic nerve	0.80 ± 0.07 (CI: [0.78–0.82]), 0.80 [0.76–0.85] (CI: [0.77–0.82])	0.41 ± 0.25 (CI: [0.34–0.48]), 0.39 [0.23–0.57] (CI: [0.26–0.50])	−0.00 ± 0.28 (CI: [−0.08–0.08]), −0.01 [−0.25–0.24] (CI: [−0.16–0.14])
Intraconal fat	0.75 ± 0.11 (CI: [0.72–0.78]), 0.76 [0.68–0.84] (CI: [0.70–0.83])	0.46 ± 0.37 (CI: [0.35–0.57]), 0.40 [0.28–0.49] (CI: [0.32–0.47])	0.34 ± 0.27 (CI: [0.26–0.42]), 0.32 [0.16–0.49] (CI: [0.23–0.44])
Extraconal fat	0.66 ± 0.17 (CI: [0.61–0.71]), 0.73 [0.53–0.79] (CI: [0.61–0.77])	0.74 ± 0.82 (CI: [0.49–0.99]), 0.42 [0.32–0.93] (CI: [0.35–0.66])	0.47 ± 0.33 (CI: [0.37–0.57]), 0.42 [0.22–0.70] (CI: [0.31–0.57])
Lateral RM	0.76 ± 0.14 (CI: [0.72–0.80]), 0.82 [0.69–0.86] (CI: [0.75–0.85])	0.32 ± 0.23 (CI: [0.25–0.39]), 0.24 [0.15–0.45] (CI: [0.17–0.34])	0.15 ± 0.26 (CI: [0.07–0.23]), 0.17 [−0.01–0.27] (CI: [0.08–0.24])
Medial RM	0.83 ± 0.09 (CI: [0.80–0.86]), 0.85 [0.80–0.88] (CI: [0.81–0.88])	0.24 ± 0.18 (CI: [0.19–0.29]), 0.19 [0.14–0.28] (CI: [0.16–0.25])	0.11 ± 0.20 (CI: [0.05–0.17]), 0.09 [−0.02–0.22] (CI: [0.02–0.15])
Inferior RM	0.84 ± 0.08 (CI: [0.82–0.86]), 0.85 [0.81–0.90] (CI: [0.82–0.87])	0.27 ± 0.28 (CI: [0.18–0.36]), 0.19 [0.14–0.31] (CI: [0.15–0.23])	0.09 ± 0.19 (CI: [0.03–0.15]), 0.07 [−0.01–0.20] (CI: [0.01–0.13])
Superior RM	0.75 ± 0.10 (CI: [0.72–0.78]), 0.77 [0.71–0.83] (CI: [0.73–0.80])	0.65 ± 0.59 (CI: [0.47–0.83]), 0.40 [0.26–0.85] (CI: [0.32–0.65])	0.15 ± 0.32 (CI: [0.05–0.25]), 0.17 [−0.02–0.31] (CI: [0.10–0.25])

To facilitate comparison with previous work [20–34], we provide in Table 2 a summary of prior studies, including reference, model used, dataset size, pulse sequence, and reported performance (DSC). While this contextualizes our results within the literature, a direct comparison is limited by differences in imaging protocols (multi-contrast MRI vs. single-modality acquisitions), segmentation targets (e.g., sclera, VH, lens, or tumor), and methodological approaches (e.g., ASM, 2D/3D U-Net).

**Table 2:** Comparison of our study with prior work on eye and orbit segmentation from MRI. The table summarizes reference, model used, dataset size, pulse sequence, and reported performance (DSC). Note that differences in imaging protocols, segmentation targets, and methodologies limit the possibility of direct performance comparisons.

Reference	Model used	Dataset	Pulse	Performance (DSC)											
				Sclera	VH	Lens	Tumor	ON	LR	MR	IR	SR	SO	IF	EF
Current	nnU-Net (3D)	3D-MR, 74 healthy subjects	T1	–	0.94	0.86	–	0.80	0.82	0.85	0.85	0.77	–	0.76	0.73
Yang	2D-3D UNet	2D-MR, 49 healthy, 32 glaucoma, 38 strabismus subjects	T2	>0.95*	–	–	–	–	–	–	–	–	–	–	–
Qureshi 2023	2D-3D UNet, DeepLabV3 + , ConResNet	2D-MR, 18 healthy, 20 with superior oblique muscle palsy subjects	T1, T2	–	–	–	–	–	0.82	0.92	0.90	0.80	0.79	–	–
Strijbis 2021	MV-CNN	2D multiview (3 planes) MR, 29 RB, 17 healthy eyes	T1c, T1, T2, FIESTA	0.84	0.93	0.91	0.84	–	–	–	–	–	–	–	–
Nguyen 2019	2D U-Net + ASM / CRF	3D-MR, 24 UM	T1, T2	–	–	–	0.84	–	–	–	–	–	–	–	–
Nguyen 2018	3D ASM	3D-MR, 7 UM, 30 healthy eyes	T1	0.95*	0.92	0.88	–	0.82	–	–	–	–	–	–	–
Nguyen 2018	3D U-Net	3D-MR, 32 RB, 40 healthy eyes^+^	T1, T2	0.95*	–	0.87	0.59	0.79	–	–	–	–	–	–	–
Ciller 2017	3DASM+3DCNN	3D-MR, 16 RB eyes	3D T1c, T1, T2	0.95*	0.95	0.86	0.62	–	–	–	–	–	–	–	–
Ciller 2015	3D ASM	3D-MR, 24 healthy eyes	3D T1c	0.95*	0.95	0.85	–	–	–	–	–	–	–	–	–

In addition, to evaluate the robustness of the nnU-Net, we computed the correlation between eye-quality scores and segmentation performance (DSC) across the 43 test images. The correlation analysis showed weak associations between DSC and subjective image quality across all structures (all |r| ≤ 0.25; see S2 Fig) and no statistically significant correlations (all p > 0.05), indicating no clear monotonic relationship within the test set and suggesting that any potential association is likely weak within the observed quality range. Due to the limited sample size (N = 43), this analysis may be underpowered and should be interpreted with caution and may not generalize to the full cohort.

### Extraction of biomarkers at large scale

After automatically delineating the anatomy of eye structures, we developed an automated procedure to compute key ophthalmic morphometry biomarkers, including millimeter-scale volumetry of eye structures and AL. This automation allowed us to extract these measurements also from the large-scale non-manually segmented dataset of 1,157 subjects, after Quality Control (QC) steps (see Quality Control Protocol in the Materials and Methods section).

**Fig 3:**
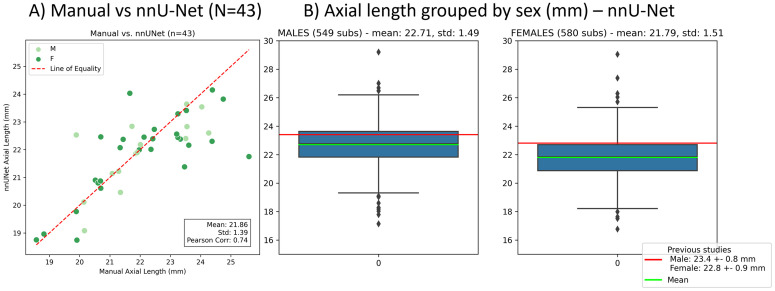
Axial length grouped by sex. A) Correlation curve and coefficient on N = 43 with respect to the subject-wise manual annotations, which serve as ground truth. The plot shows moderate to high correlation between both sets. B) Boxplots of the obtained AL grouped by sex computed on NR = 1129. The plots show values close to the references.

Our findings show that automated measurements of AL from MRI are in line with the reference manual measures [40]. Fig 3 shows the correlation plot of the extracted values, grouped by sex as in [22,40], on the manually annotated cohort of 43 testing subjects. The average AL from nnU-Net segmentation was closer to the GT values which were extracted using the same AL extraction method on the manual segmentation. For the large-scale cohort of 1157 subjects, the mean values and standard deviations are close to the manual reference as well:

- nnU-Net (N=1129): 22.71±1.49 mm (549 M) and 21.79±1.51 mm (580 F)
- Previous studies [40]: 23.4 ± 0.8 mm (1059 M) and 22.8 ± 0.9 mm (867 F)

However, in 28/1157 (2.42%) cases, after manual inspection, the AL could not be computed due to: (1) 20 missing lens segmentation, typically caused by poor visibility or absence of the lens on the T1w images; (2) 7 instances where the left eye was segmented instead of the right; and (3) 1 case where the lens centroid did not fall within any lens voxel, due to disconnected components in the segmentation (no post-processing applied after inference). To evaluate potential bias introduced by the remaining 28 cases with missing AL measurements, we analyzed their demographic and image-quality characteristics. This subset included 19 males and 8 females, with a mean age of 63.5 ± 11.8 years and a mean BMI of 30.5 ± 4.4 kg/m². Image quality was generally lower than average, often showing left–right phase-encoding artifacts near the eyes. Overall, these characteristics are comparable to those of the complete cohort, indicating that the small number of missing AL cases is unlikely to affect the overall volumetric or atlas results. Lens detection showed a high success rate (1137/1157, 98.3%). These findings indicate that most AL extraction failures were driven by image limitations rather than instability of the segmentation model.

**Fig 4:**
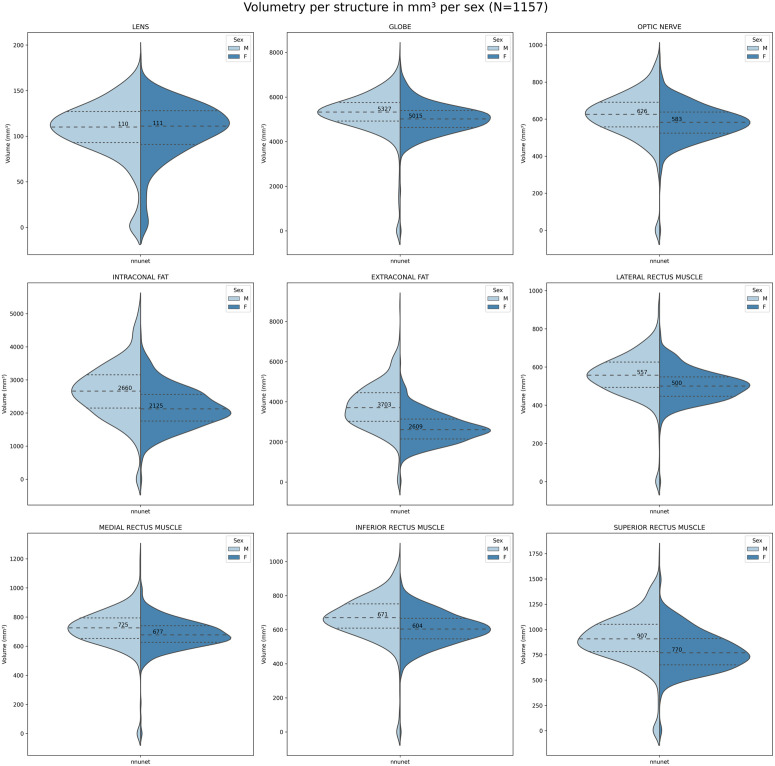
Volumetry per method for each eye structure per sex (568 males and 589 females). Median values in mm^3^ are provided on each plot.

In terms of volumetry extraction, we provide the first large-scale benchmark MR-Eye volumetry of all eye structures. Descriptive statistics, including mean ± standard deviation, median with interquartile range, coefficients of variation (CV%), and 95% confidence intervals, are reported in Table 3. Unadjusted analyses showed significantly larger volumes in males compared to females for all structures except the lens (all FDR-corrected p < 0.05), with small-to-moderate effect sizes (see S3 Table). After adjustment for body height (and age), several sex differences remained significant, particularly for intraconal and extraconal fat, whereas others were attenuated, indicating that part of the observed differences is explained by anthropometric variability (see S4 Table). Fig 4 illustrates the distribution of volumetric measurements per structure, grouped by sex, using violin plots from the large-scale cohort of 1,157 subjects.

**Table 3:** Volumetric measurements of orbital structures by sex (568 males and 589 females). Values are reported as mean±standard deviation (SD), median with interquartile range (IQR), 95% confidence intervals (CI), and coefficient of variation (CV).

Structure	Sex	Mean ± SD (95% CI mean)	Median [IQR] (95% CI median)	CV (%)
Lens	Male	106.16 ± 33.38 (103.41, 108.91)	110.00 [93.00–127.00] (107.00, 113.00)	31.44
Lens	Female	105.72 ± 31.95 (103.13, 108.31)	111.00 [91.00–128.00] (109.00, 113.00)	30.22
Globe	Male	5218.60 ± 1086.09 (5129.09, 5308.11)	5327.50 [4922.25–5752.75] (5263.50, 5395.00)	20.81
Globe	Female	5020.03 ± 704.02 (4963.06, 5077.01)	5015.00 [4637.00–5398.00] (4969.00, 5094.00)	14.02
Optic nerve	Male	616.00 ± 136.44 (604.76, 627.25)	626.00 [558.00–691.25] (617.00, 639.00)	22.15
Optic nerve	Female	581.57 ± 95.46 (573.84, 589.29)	583.00 [524.00–638.00] (574.00, 592.00)	16.42
Intraconal fat	Male	2657.43 ± 839.44 (2588.24, 2726.61)	2660.00 [2149.75–3152.75] (2576.00, 2746.00)	31.59
Intraconal fat	Female	2164.31 ± 612.88 (2114.71, 2213.91)	2125.00 [1756.00–2559.00] (2040.00, 2188.00)	28.32
Extraconal fat	Male	3751.62 ± 1134.92 (3658.09, 3845.15)	3703.00 [3026.75–4451.50] (3602.00, 3832.00)	30.25
Extraconal fat	Female	2687.78 ± 752.32 (2626.89, 2748.66)	2609.00 [2146.00–3138.00] (2552.00, 2678.00)	27.99
Lateral RM	Male	553.20 ± 123.65 (543.01, 563.39)	557.00 [493.50–626.00] (548.00, 567.00)	22.35
Lateral RM	Female	502.53 ± 82.95 (495.82, 509.24)	500.00 [447.00–548.00] (494.00, 508.02)	16.51
Medial RM	Male	719.08 ± 143.29 (707.27, 730.89)	725.50 [653.00–794.00] (714.00, 738.00)	19.93
Medial RM	Female	679.70 ± 104.06 (671.28, 688.13)	677.00 [626.00–740.00] (667.00, 686.00)	15.31
Inferior RM	Male	667.28 ± 139.46 (655.79, 678.77)	671.00 [609.00–751.25] (662.00, 680.50)	20.89
Inferior RM	Female	604.96 ± 97.51 (597.07, 612.86)	604.00 [546.00–667.00] (595.00, 615.00)	16.12
Superior RM	Male	904.34 ± 255.13 (883.31, 925.36)	907.50 [782.00–1052.00] (884.00, 935.00)	28.21
Superior RM	Female	787.05 ± 193.12 (771.42, 802.68)	770.00 [651.00–910.00] (751.00, 792.00)	24.54

**Fig 5:**
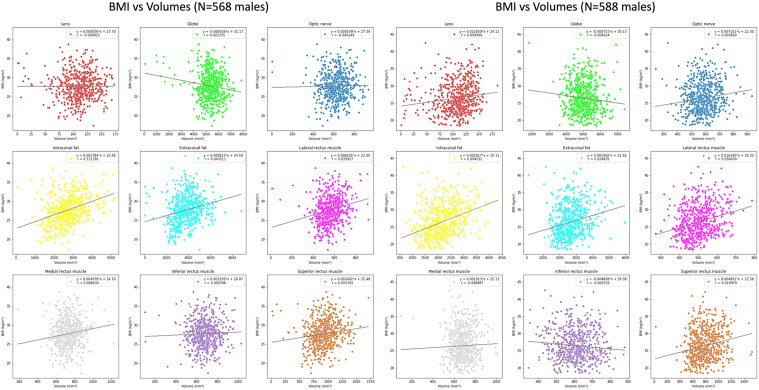
Correlation of volumetry per structure and BMI grouped by sex. There is no existing correlation between BMI and volumetry per structure based on the Huber R2 scores for any of both sex cases in any of the eye structures (the scores are lower than 0.3, indicating the lack of correlation).

Interestingly, robust linear regression (Huber) showed low coefficients of determination (R^2^ < 0.1 in most cases), indicating limited explanatory power of BMI. Fig 5 illustrates these relationships with scatter plots and Huber regression lines, grouped by sex. To account for potential confounding factors, we further performed adjusted robust regression analyses including age and body height as covariates. BMI remained significantly associated with most orbital structure volumes in both sexes (all FDR-corrected p < 0.05, except for lens volume in males), although effect sizes remained modest (see S5 Table). Consistent with these findings, Pearson correlation analysis revealed small to moderate effect sizes (r = 0.11–0.38, all p < 0.001 after Benjamini–Hochberg correction), corresponding to low explained variance (R^2^ < 0.15 across all structures, see S6 Table). These results indicate that BMI has an independent but limited contribution to the variability of orbital tissue volumes.

### Validation on left eyes

To evaluate model performance on left eyes, we conducted dedicated validation using the same cohort.

Regarding AL, the proportion of cases where it could not be computed for left eyes (22/1157 = 1.9%) was comparable to that observed for right eyes (28/1157 = 2.42%). Most failures were again due to missing lens visibility in the T1-weighted images (20/22 cases), with the remaining two cases occurring when the lens centroid fell on a non-lens voxel. Among the cases with missing lenses, seven subjects overlapped between left and right eyes, indicating that in these images the lenses were poorly depicted in both eyes. In total, 33 subjects presented missing lenses in at least one eye. After excluding missing values, nnU-Net-derived AL measurements for left eyes (N = 1135) were 22.43 ± 1.47 mm in males (N = 557) and 21.51 ± 1.75 mm in females (N = 578). The within-subject correlation of AL between left and right eyes remained moderate but statistically significant (Pearson’s r = 0.73, p < 10 ⁻ ¹⁰). Quantitative comparisons showed minimal inter-eye differences (mean difference −0.16 mm, mean absolute difference 0.65 mm, relative asymmetry −0.81%), indicating strong bilateral symmetry despite the moderate correlation.

To further assess bilateral consistency, we compared left and right eye volumetry within subjects. Total orbital volumes (i.e., the combined volume of all segmented structures) showed a very high correlation (N = 1157, Pearson’s r = 0.96, p < 10 ⁻ ¹⁰). To evaluate whether the observed Pearson correlation between left- and right-eye measurements could be explained by random pairing, we performed a permutation test. The right-eye measurements were randomly permuted across subjects 5000 times, thereby disrupting the original within-subject correspondence while preserving the marginal distribution of the data. For each permutation, Pearson’s correlation coefficient was recalculated to generate a null distribution under the hypothesis of no within-subject association. As expected, the null distribution was centered near zero. None of the permuted correlations was as large as the observed correlation, yielding an empirical permutation p-value of 2 × 10 − 4. These findings indicate that the observed inter-eye correlation is unlikely to have arisen by chance and support the presence of a genuine bilateral association within subjects. Beyond correlation, quantitative comparisons demonstrated low mean absolute differences and small relative asymmetry between eyes across structures, with no relevant systematic bias at the global level. In particular, total orbital volume exhibited minimal inter-eye differences, with a mean absolute difference of 465 mm^3^ (0.465 mL) and a relative asymmetry of −1.17% (SD = 4.40%), supporting a high level of bilateral consistency. While small but statistically significant differences were observed for several individual structures, these remained limited in magnitude (typically < 3%) and are likely attributable to a combination of subtle physiological asymmetry and segmentation variability in smaller or less well-defined tissues, such as the lens. Larger structures showed negligible asymmetry and no significant bias, further supporting the robustness of the segmentation. These results are provided in S7 Table.

Additionally, a qualitative evaluation of the segmentation results was also performed on a randomly selected subset of 10 T1w images from the full cohort (N = 1210). A trained ophthalmologist (author AKL) visually assessed all structures for the left eye segmentations. Across all subjects and structures, the mean score was 3.42/4 (0.86), indicating overall good segmentation quality. Most structures received scores between good and excellent, with the highest average score observed for the globe (3.8). The lowest average score corresponded to the extraconal fat (2.6), which reflects the more diffuse boundaries of this structure in MRI. In one case, the lens segmentation was missing due to the absence of a clearly visible lens in the corresponding T1w image. These results are reported in S8 Table.

### Atlas of the eye

**Fig 6:**
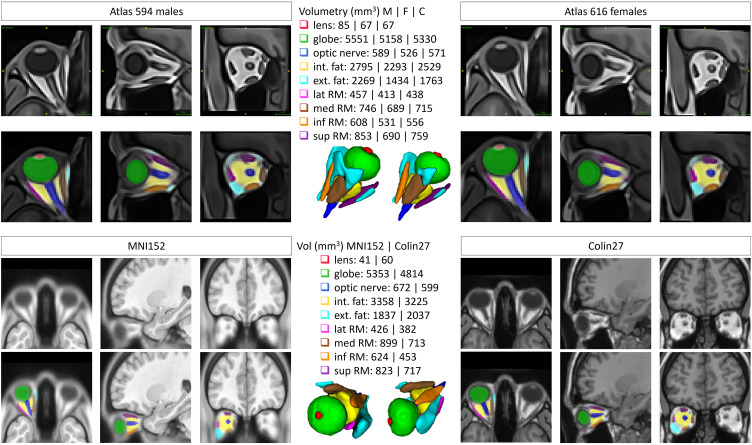
Eye atlases. Male and female atlases of the eye (above). At the top for each sex case, the three views of the T1w atlas made, below them, the probability maps of the labels projected onto the atlas' space, and at the center, the 3D-rendered maximum probability maps of these labels along with the volumetry per structure, male (M), female (F), and combined (C), in order. Eye labels projected onto T1w MNI152 and Colin27 VCS (below). Captures of the axial, sagittal and coronal views, and 3D render of the eye structures with their volumetry. MNI152 and Colin27 VCS shown on the left and right, respectively.

We present the first large-scale unbiased eye atlases in MRI. The male, female, and combined eye templates come with their corresponding probability maps of the different labels, which are publicly released [59]. Fig 6 shows male and female cases. The volumes of these maps indicate similar structure sizes for both sexes, except for the fat, which is larger in males. We also provide accurate eye labels onto MNI152 and Colin27 VCS. Fig 6 shows the resulting labels projected onto these common reference spaces. Using the male atlas reference for Colin27 and the combined atlas reference for MNI152, the volumes from Colin27 are generally close to their references, while those from MNI152 are generally larger. For both cases, lenses and intraconal fat were notably different.

The atlas validation indicated overall good agreement between the automatically generated labels and the manual atlas segmentation (see S9 Table). Similarity metrics (DSC, HD and VD) were computed for all structures. The results demonstrated high agreement for well-defined anatomical structures, including the globe (DSC = 0.92, HD = 0.10) and extraocular muscles (DSC = 0.56–0.84), with moderate agreement for more anatomically variable regions such as extraconal fat.

## Discussion

MR-Eye has increasingly gathered interest in the ophthalmic and radiology communities [10], due to the tissue contrast that it can achieve in a non-invasive way. Furthermore, and unlike most ophthalmic tools which evaluate the anatomy or the visual performance of the eyes (OCT [5,6], biometry [60], microperimetry [61], eye-tracking, contrast sensitivity), MR-Eye can investigate several pathologies behind the globe, involving nerve paralysis, lesions, tumors, and inflammation [7,11,62], while exploring the 3D complexity of the eye-shape. In fact, 3T and 1.5T MR-Eye clinical protocols are used regularly in the case of tumor—retinoblastoma [63,64] or uveal melanoma [65–67]—or ocular inflammations [10,40,65,68], or pathologies with suspected link to the brain [62], and constitute the current state of the art of clinical practice. Very recent technical advancements propose new ways to deal with the presence of motion artefacts during MR-Eye acquisition [69–74] or at ultra-high field (7T) [15,75], increasing the usability and reproducibility of MR-Eye in ophthalmology.

In this rapidly growing field, it is crucial to enable clinicians to extract measurements from MR-Eye and benchmark new metrics, providing them with tools not available before. To address this need, we propose a comprehensive automated pipeline. The pipeline is fully automatic and does not require manual cropping of the eye region. Moreover, thanks to the nnU-Net framework, the model can handle input images of varying sizes and resolutions, so the inputs do not need to match the training dimensions exactly. This flexibility allows the pipeline to be applied directly to diverse MRI datasets without additional user intervention. This is benchmarked on a large-scale MR-Eye database of post-QC 1,157 subjects and introduces a methodology for automated 3D segmentation (Fig 1) of all eye structures using a deep-learning algorithm (nnU-Net). It enables extraction of key ophthalmic biomarkers, such as AL and volumetry, and allows us to build the first large-scale comprehensive eye atlas for both males and females, as well as the joint one, with their corresponding probability maps. For further applicability, these large-scale atlases were also projected onto common VCS.

Our automated 3D segmentation algorithm via DL-CNN (nnU-Net) of all eye structures, once compared with manual segmentations performed by expert ophthalmologists on 43 testing subjects, is optimal with respect to classic image segmentation metrics, namely DSC, HD, and VD. We previously reported results on the same cohort, comparing them with a baseline (atlas-based) segmentation method in our preliminary work [76], with statistical analysis. The results obtained in this study with nnU-Net are in line with previous reported values of segmentation performance for lens, globe, and optic nerve [23,24,26,27,31,32], despite the fact that they relied on multi-contrast MRI, and healthy and non-healthy eyes, including tumors such as retinoblastoma [26,27,31] and uveal melanoma [24,32]. A comparison table of the performances of these previous methods can be found in Table 2, with DSC ranges for lens of [0.77, 0.91], for globe (referred to as VH) of [0.92, 0.95], and for sclera (in some cases including the VH) of [0.84, 0.95], with few reports of DSC values of the optic nerve [0.79, 0.82], rectus muscles or fat. While the validation cohort of 43 subjects is modest compared to the overall dataset, the automated segmentations applied to the remaining 1,157 subjects produced axial length distributions consistent with state-of-the-art manual measurements reported in the ophthalmological literature [22,40], supporting the robustness of our findings within this dataset. However, the relatively small number of manually annotated cases used for training and the absence of external validation across different scanners, field strengths, or populations remain important limitations. Therefore, the nnU-Net results should be interpreted as an internal proof-of-concept rather than evidence of full generalizability. In this context, the proposed web platform provides a practical framework for future validation by enabling the application and evaluation of the model on data acquired with different scanners, contrasts, and imaging protocols, thereby supporting the assessment of generalizability in multi-center settings.

Additionally, it would also be valuable to assess inter-rater variability by including multiple independent manual annotations, as in [20], where inter-rater agreement was quantified via ICC. However, in our study only a single manual segmentation per subject was available, which prevented such an analysis. We acknowledge this limitation; however, prior studies on MRI-based orbital segmentation report moderate-to-good inter-rater agreement (ICC ≈ 0.5–0.8), particularly for structures with less well-defined boundaries such as extraocular muscles and orbital fat [77,78]. Therefore, the reported segmentation performance (DSC = 0.80 ± 0.07) should be interpreted as agreement with this expert-defined reference rather than as a direct comparison to human inter-rater variability. We acknowledge this as a limitation and consider it an important direction for future work to further contextualize the robustness of automated segmentation relative to human variability.

Another limitation is that only right eyes were manually annotated in our dataset. However, we demonstrated that the trained model can be successfully applied to left eyes by reorienting images to a common space, cropping the left-eye region, and mapping the resulting segmentations back to the original space. Quantitative comparisons showed close correspondence between left and right eyes for axial length and total orbital volume, with only small inter-eye differences. These differences were limited in magnitude and likely reflect a combination of subtle physiological asymmetry and segmentation variability, particularly for smaller or less well-defined structures. Consistent with prior cadaveric [79] and CT-based [80] studies reporting high bilateral similarity of orbital volumes, our results support a high degree of left–right correspondence, with only minor differences in healthy individuals and without evidence of a systematic directional bias (i.e., one eye being consistently larger than the other). Additionally, a qualitative evaluation performed by an expert ophthalmologist on a subset of 10 subjects yielded high segmentation quality scores, further supporting the robustness of the segmentation pipeline for both eyes.

Our study reports the anatomical delineation (e.g., volumetry) of structures such as orbital fat and rectus muscles directly extracted in 3D—RM segmentation has so far been presented only in 2D [27]. Moreover, our automated segmentation completes in less than one minute per eye (speed depends on the GPU). With its high accuracy, it could be seamlessly integrated into MRI console analysis, potentially saving clinicians 10–20 minutes (according to SL, senior radiologist) they currently spend on manual segmentation. Additionally, we aim to adapt our segmentation to handle variations in contrast and spacing, aligning with the current state-of-the-art MR-Eye protocols, which include T1w imaging, fat-suppressed T1w and T2w imaging, and contrast injections [7,8,11,62]. Incorporating uncertainty quantification for automated predictions can be beneficial to such scopes [81].

To ensure the removal of low-quality images that could compromise the results, we introduced QC protocols at multiple stages of the segmentation pipeline. Inspired by the state-of-the-art method MRI-QC [38], we observed a mismatch between low-quality images identified by the MRI-QC toolbox and those identified by our MR-Eye experts. This suggests that QC in MR-Eye requires different metrics and criteria compared to brain imaging. The moderate-to-good inter-rater agreement obtained for the QC ratings supports the consistency of the proposed visual QC protocol. Nevertheless, future work should aim to develop objective quantitative metrics tailored specifically to orbital MRI, incorporating non-tissue metrics and extending scrutiny to the periorbital region to further standardize image quality assessment.

To further evaluate the pipeline, we implemented an automated method to estimate AL from the segmented MR-Eye volumes. The automated measurements showed good agreement with reference AL values reported in the literature and with manual measurements obtained in the test set of 43 subjects. In the large-scale cohort, AL could not be computed in 2% of cases (28/1157), primarily due to missing lens segmentation caused by poor visibility or absence of the lens in the T1w images, occasional laterality selection errors, or disconnected segmentation components. These failures suggest that most limitations were related to image characteristics rather than instability of the segmentation model. Potential mitigation strategies include quadrant eye segmentation (as we do for the left eyes), connected-component post-processing, and stricter QC procedures to exclude images with insufficient lens visibility.

From a clinical perspective, several ocular conditions are unlikely to substantially degrade MRI-based lens detection. Cataract-related lens opacification is defined by optical transparency and does not necessarily reduce MRI contrast; previous studies have even reported increased lenticular MRI signal intensity with age [82], which may facilitate lens delineation. Likewise, in post-surgical eyes such as pseudophakia, the interface between the implant or residual capsule and the vitreous body typically remains sufficiently visible to define lens boundaries. However, severe anatomical alterations, such as lens dislocation, major orbital trauma, or vitreous substitutes (e.g., gas or silicone oil), may still pose challenges for automated segmentation and warrant further investigation. Nevertheless, these cases often present as acute clinical emergencies where MRI is not the primary diagnostic modality. Finally, the cornea is a very thin structure (~550 µm), below the spatial resolution of typical MRI acquisitions (~1 mm), which explains why its detection can be sensitive to acquisition conditions and is facilitated when images are acquired with the eyes closed.

MRI-based AL measurements are intended as a robust anatomical proxy of partial coherence interferometry (optical biometry), not as a replacement. While optical methods are the clinical gold standard for refractive measurements, MRI-based AL provides comparable accuracy (millimeter versus micro-meter precision) in cases with dense cataract changes and silicone oil filling the vitreous cavity [83], in highly myopic patients [84], or for research purposes in studies that do not have biometry to study neurodegenerative changes and to control for differing eye size across individuals [41]. It may also provide valuable information in specific clinical scenarios, such as patients with rhegmatogenous retinal detachments with macular detachment or vitreous and submacular hemorrhage, where preoperative axial length is often underestimated [85]. Methodologically, our definition (posterior cornea to posterior globe boundary) follows established imaging standards where the corneal and retinal thicknesses approximately cancel each other out, minimizing the net error in clinical MRI sequences [83]. Unlike optical biometry, which can be affected by opacities or reflections in staphylomatous eyes, MRI offers an investigator-independent, reproducible measurement that is directly transferable to other cross-sectional modalities like CT or ultrasound. For clinical applications requiring the fusion of different modalities, previous studies have already demonstrated the feasibility of registering MRI datasets with ultrasound or biometric data to ensure comparability [86].

We also provide large-scale benchmarks for volumetry of all eye structures at a millimeter scale. Compared with previous studies reporting volumetric measurements for selected structures in cm³ [43–45], our work extends these findings by providing a comprehensive and detailed characterization of all major orbital compartments in a large cohort. While unadjusted analyses showed generally larger volumes in males compared to females across most structures, these differences were attenuated after adjustment for body size, indicating that they are partly explained by anthropometric variability. Nevertheless, several structures—particularly intra- and extraconal fat and the globe—remained significantly different after adjustment, suggesting that both body size and intrinsic anatomical differences contribute to sex-related variability. Importantly, effect sizes were modest, indicating that sex alone explains only a limited proportion of volumetric variability. This sex-wise differentiation in eye structure volumetry could have relevant implications for understanding sex-specific ophthalmological conditions and tailoring more personalized medical treatments, particularly as such differentiation is increasingly needed for better health care [56,57]. The reported measurements also highlight differences in variability across structures, with higher coefficients of variation observed for fat tissues compared to more compact structures such as the globe or optic nerve. This reflects the diffuse anatomical nature of orbital fat and its greater inter-individual variability. From a clinical perspective, the volumetric values reported here should be interpreted as reference distributions rather than diagnostic thresholds. Absolute quantification of certain tissues, particularly orbital fat, may be less informative than relative changes, asymmetry, or patterns of tissue expansion within the orbital cavity. Future work could apply the proposed automated segmentation framework to patient cohorts and compare volumetric measurements with healthy controls, which may be particularly relevant for conditions such as Graves’ orbitopathy and other orbital pathologies.

Interestingly, although previous work [40] reported a significant association between exophthalmometry (defined as the perpendicular distance between the interzygomatic line and the posterior surface of the cornea) and both axial length and BMI (p < 0.001), our investigation revealed only weak—albeit statistically significant—associations between BMI and eye structure volumes. This suggests that BMI explains only a limited proportion of the variability in orbital tissue volumes. One possible explanation for this discrepancy lies in the different phenotypes being measured. Exophthalmometry quantifies the anterior displacement of the globe relative to the lateral orbital rim and is therefore strongly influenced by the capacity and geometry of the bony orbit. In contrast, our MRI-based volumetric analysis directly measures intraorbital soft tissues. As a result, increases in orbital fat volume (e.g., Graves’ disease) may be partially accommodated by orbital architecture and not necessarily translate into measurable proptosis. By focusing on tissue volume rather than globe position, our approach captures a distinct anatomical phenotype, which may explain the weaker associations observed. We further accounted for potential confounding factors by including age and body height in adjusted regression models, which confirmed that BMI has an independent but modest association with orbital volumes. Although head or orbital size could also influence these relationships, we did not include a head-size covariate in the final models. Automated MRI-derived head masks showed variability across subjects (e.g., due to differences in field-of-view and head segmentation consistency), limiting their reliability as a covariate. Moreover, global head size represents only an indirect proxy for orbital capacity. Future work incorporating direct measures of orbital or craniofacial anatomy may help further clarify these relationships.

Our study introduces a novel method for automated biomarker extraction, paving the way for benchmarking MR-Eye-derived measurements of the adult human eye. The implications of these findings are several and open the way to a broader use of MRI in ophthalmology, potentially enhancing diagnostic precision, informing surgical planning, improving our understanding of eye anatomy across different populations, and saving clinicians’ time. Future research should aim to further validate these methods in pathological eyes and explore additional biomarkers. For instance, evaluating changes in RMs is key in pathologies such as strabismus [69,87], or open to the evaluation of new elements such as cerebrospinal fluid (CSF), whose deposit in the optic nerve plays a crucial role in pathologies such as papilledema and glaucoma [88,89].

The SHIP cohort is representative of the population in Northeastern Germany and consists of more than 99% individuals of Caucasian descent. While this provides a robust anatomical reference for European populations, it limits the generalizability of our findings to other ethnic groups. Previous studies have reported clinically relevant ethnic differences in ocular biometry, including axial length, orbital volume, and the prevalence of myopia, particularly in East Asian populations [90]. These differences may influence both absolute volumetric measurements and the relative proportions of orbital structures. At the same time, studies based on external eye morphology derived from photographic analyses have suggested that several global shape descriptors (e.g., eye height, length, and ellipticity) are relatively consistent across ethnicities, with variability often dominated by inter-individual differences rather than ethnic group effects [91]. However, such findings primarily reflect surface anatomical features and may not directly translate to internal volumetric characteristics assessed by MRI. Therefore, the atlas presented in this work should be interpreted as reflecting a predominantly Caucasian phenotype. Its applicability to other populations remains to be established and warrants validation in multi-ethnic cohorts.

To further improve the usability of MR-Eye in clinics and research, we present pioneering male, female and combined eye MRI atlases, along with their detailed labels, estimated on a large-scale cohort. Atlases are crucial in research as reference tools for registration and segmentation in population imaging studies. In clinical practice, they can facilitate the diagnosis and treatment of a wide range of ocular diseases, help to reveal abnormal structural changes, enhance surgical planning, and improve our understanding of sex-specific variations in eye anatomy and physiology [48]. These atlases offer a valuable resource for advancing the study of ocular anatomy and can significantly support the accuracy of eye-related research and clinical applications, as has been largely demonstrated for brain studies [48–50,52,92]. Furthermore, the sex-based differences observed emphasize the relevance of separate male and female atlases capturing anatomical nuances. Regarding common VCS, similar volumes were found in MNI152 and Colin27 with respect to their references highlighting the applicability of the proposed atlases to further studies.

Although the atlas labels were derived from automated segmentations, the aggregation of a large cohort through majority voting is expected to reduce the impact of isolated segmentation errors. The additional manual atlas validation further supports the anatomical plausibility and overall consistency of the resulting atlas representation. Importantly, the similarity metrics extracted from that validation are not directly comparable to the subject-level nnU-Net evaluation (N = 43), as the atlas manual segmentation follows a slightly different annotation protocol. In particular, the manual atlas included a more extended optic nerve, a larger and fully connected extraconal fat compartment, and an anterior globe region surrounding the lens, leading to systematic volumetric differences and reduced overlap metrics. Despite these differences, the observed spatial agreement remains consistent with expected anatomical variability, supporting the validity and anatomical coherence of the atlas labels derived from large-cohort aggregation. To promote transparency and reproducibility, the manual atlas segmentation is now provided in the supplementary materials and will be made publicly available through the existing dataset on Zenodo [59].

MR-Eye has been thus far indispensable when other ophthalmologic imaging modalities fail [7–10], but recent studies, aiming at improving its usability, showcase the interest of using MRI in ophthalmology. In the context of these recent advancements, we demonstrated the feasibility and accuracy of large-scale automated segmentation and biomarker extraction, proposing a ready-to-use solution which promotes the adoption of MR-Eye in the clinical and research setting.

## Materials and methods

### Experimental design

To rigorously assess MR-Eye, we first validated a deep learning–based automated segmentation method on manually segmented subjects using similarity metrics (surface overlap, volume error, and distance-based error). Building on this, we extracted key ophthalmic biomarkers—volumetry of eye structures and axial length—across the large-scale cohort to enable reproducible and clinically relevant measurements, including correlations with BMI stratified by sex. Dedicated eye-quality control checks, described later, ensured robustness and mitigated imaging artifacts. Together, these components form an integrated pipeline, with each step supporting reliable and generalizable MR-Eye analysis.

### Dataset

The cohort was originally acquired as part of the Study of Health in Pomerania (SHIP) [40,93–95] and reused for the present study. Whole-body MRI data was obtained from 3030 adult participants drawn from the SHIP-2 and SHIP-Trend cohorts. The SHIP study is a population-based cohort from Northeastern Germany and is demographically representative of this region, with more than 99% of participants of Caucasian (European) descent [94].

Based on DICOM metadata provided with the dataset, the MRI examinations used in the present study were performed between 03/06/2008 and 21/11/2012 on a 1.5T Magnetom Avanto scanner (Siemens Medical Solutions, Erlangen, Germany) without contrast agent. For all MRI measurements, the image bisecting the eyeball in the axial plane and containing both the corneal apex and the optic nerve head was selected. Participants were excluded if such a plane was not available, if their viewing direction deviated laterally, or if image quality was insufficient (e.g., motion artefacts or technical issues). Due to these exclusion criteria, summarized as “low image quality,” 549 subjects were excluded. In some cases, only one eye was evaluable, or only axial length measurement was possible, leading to the exclusion of an additional 555 participants. Following further SHIP quality control in 2023, 681 subjects were removed due to insufficient quality. The final dataset included 1245 subjects (age range 28–89 years, mean 56 ± 13).

T1w images of the head were acquired using a 12-channel head coil (176 slices per volume, 1 mm slice thickness, 256 mm field of view, 1 mm³ voxel size, TR = 1900 ms, TI = 1100 ms, and TE = 3.37 ms). During MRI acquisition, subjects rested their eyes naturally without specific instructions regarding gaze or eyelid position.

All imaging procedures in SHIP were approved by the Medical Ethics Committee of the University of Greifswald, and all participants provided written informed consent. The data used in this study were accessed as anonymized records.

VCS datasets: MNI152 T1w (152 participants) [50], and Colin27 T1w (1 male scanned 27 times) [51].

#### Manual segmentation protocol.

Manual annotations on a total of 74 subjects were done, using ITK Snap software [95], by two ophthalmologists: one senior (20 years of experience) and one junior (1 year of experience). The first batch of 35 subjects was annotated by PS, and the remaining 39 by AKL. The senior ophthalmologist (OL), reviewed all annotations and corrected them when necessary, ensuring consistency and quality control across the dataset. These manual annotations included 9 regions of interest (ROIs) for the right eye: lens, globe, optic nerve, intraconal and extraconal fats, and the four rectus muscles (lateral, medial, inferior, and superior), see Fig 1B.

#### Subjective quality evaluation.

**Fig 7:**
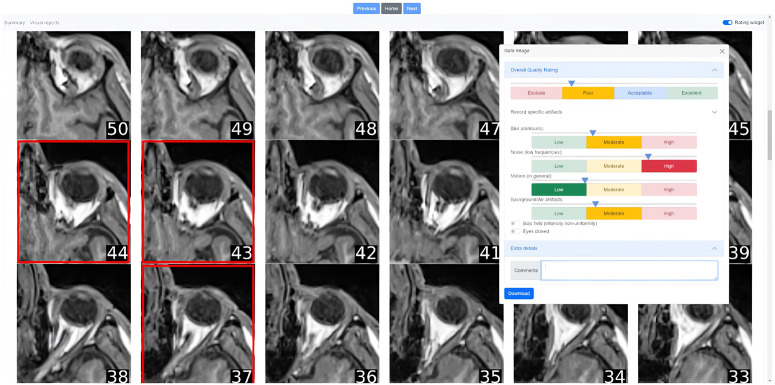
Example of MR-Eye QC report with rating widget. To assess the quality of the eyes of the MR images, we created an HTML-based report for each of them: a series of axial slices centered and cropped on the right eye. The rating widget on the right is composed of several sliders regarding overall quality [0-4], blur, noise, motion, and background artifacts. Also, it includes two toggle buttons for bias field and eyes closed/open and a text box for further comments. Additionally, it is possible to select specific slices where heavy artifacts are present (red squares will appear).

To obtain the subjective eye-quality of the 43 images in the test set, two engineer experts in ophthalmic MR image analysis (20 and 5 years of experience) independently evaluated image quality using a structured visual quality control (QC) protocol adapted from MRIQC reports [38]. These reports consist of an HTML file per subject presenting multiple axial thumbnails as well as sagittal and coronal views to assist visual inspection. A rating widget was provided including several components to evaluate specific image artefacts such as blur, noise, motion, and background air artefacts. We modified the original MRIQC reports to better suit orbital imaging by centering the thumbnails on the right eye and adding eye-specific aspects to the rating interface, such as eye open/closed status (Fig 7).

The evaluation followed a guided workflow in which raters first assessed individual image artefacts using dedicated sliders and then assigned an overall quality score on a 0–4 scale (0 = excluded, 1 = poor, 2 = acceptable, 3 = good, 4 = excellent). The final rating therefore reflected both the presence of artefacts and the visibility of relevant orbital structures, including external structures (globe and lens) and internal structures (optic nerve and extraocular muscles), evaluated across the three orthogonal views of the 3D MRI volume. Inter-rater reliability for the overall QC rating showed moderate-to-good agreement between raters (ICC(2,1) = 0.71, 95% CI: 0.54–0.81). Detailed QC annotation guidelines used by the raters are provided in S10 File to improve transparency and reproducibility of the evaluation protocol.

### Automated segmentation method: nnU-Net

NnU-Net [58] is the state-of-the-art supervised deep learning-based segmentation approach in which data augmentation is extensively used and the hyperparameters are automatically optimized. It has never been evaluated for MR-Eye, but with OCT [96]. We split the manual annotated dataset into 31 for training and 43 for testing. The split reflected data availability. Initially, 35 subjects were available, 31 were used for training / validation, and 4 for testing. After receiving 39 additional segmentations, the 4 initial test cases were added to this larger cohort to increase the test set (43 in total).

All hyperparameters were determined using the default nnU-Net experiment planning pipeline without manual tuning. In particular, the patch size [128, 112, 160], batch size (2), and network topology were automatically derived from the image spacing (1 mm isotropic), median image size (176 x 256 x 176 voxels), and available GPU memory. The resulting configuration consisted of a single 3D full-resolution stage with five-fold cross-validation during training. Training was performed using an initial learning rate of 0.001 with a ReduceLROnPlateau scheduler, the ADAM optimizer, and deep supervision with a combined cross-entropy and Dice loss function. Data augmentation followed the default nnU-Net strategy, including scaling and rotation within anatomically plausible ranges to improve generalization while preserving the structural integrity of fine orbital anatomy. Kaiming-He (0.01) weight initialization was used, and no postprocessing was applied after inference. Training was performed for up to 1000 epochs, with an elapsed time of approximately 140–170 seconds per epoch. The model included 10 classes (9 ROIs plus background). Computations were carried out on an HPC (High Performance Computing) SLURM-based cluster using GPUs (RTX 2080 and RTX 3090), 10 CPUs per fold, and 64 GB RAM, within Docker containers accessed via Singularity, using PyTorch and Python 3.8. The total training time for the five folds was approximately 208 h 20 min. The inference time was approximately 1 minute per image. Processing the full non-labeled dataset (1157 subjects) required 66,185.53 seconds (18 h 23 min 05 s) using an RTX 3060 Ti GPU. Training curves are provided in S11 Fig. to further support the observed stability of the training process.

To segment left eyes, all images were first reoriented to a common RAS (Right-Anterior-Superior) space and the quadrant containing the left eye was cropped prior to inference. The model, trained on right-eye anatomy, was then applied to these cropped left-eye images. The resulting segmentations were mapped back to the original image space via inverse cropping. The total processing time per image is around 15 s. This approach enabled consistent segmentation of left eyes and allowed merging of left and right eye segmentations into a single volume per subject for downstream volumetric and biometric analyses.

#### Evaluation: segmentation similarity metrics.

To adequately assess the performance of the segmentation method, we computed complementary similarity and error metrics between the ground truth (manual segmentation) and the method’s outputs on the right eye. Based on [97], appropriate metrics to evaluate semantic segmentation of biomedical images are:

• Dice Similarity Coefficient (DSC): it is defined as twice the number of elements common to both sets divided by the sum of the number of elements in each set. The DSC ranges between 0 (indicating no overlap) and 1 (indicating perfect overlap). It is negatively biased by small structures. $DSC=\frac{\text{2}|A\cap \hat{A}|}{\left|A|+|\hat{A}\right|}$, where *A* represents the ground truth and *Â* the predicted area.
• Hausdorff Distance (HD): it measures how far two subsets of a metric space are from each other. It is the greatest of all the distances from a point in one set to the closest point in the other set. It does have units, which are the same as the units of the coordinate space in which the points are defined, mm in our case. The HD can range from 0 to infinity (no overlap between the objects). In Fig 2, this is limited to [0, 3].$d_{H}(X,Y)=\text{max}\{\text{sup}d(x\in X,Y),\text{sup}d(X,y\in Y)\}$.
• Volume Difference (VD): it refers to the difference in the amount of three-dimensional space occupied by two objects. The VD can range from −2 (if the second volume is larger) to +2 (if the first volume is larger). In our case, the first volume is the ground truth (manual segmentation) and the second is the nnU-Net segmentation volume. Hence, having a positive VD means that the manual volume is larger than the corresponding method one, and a negative VD means that the method volume is larger than the manual.$VD\;=\frac{\text{2}*(v\text{1}-v\text{2})}{v\text{1}+v\text{2}}$.

#### Evaluation: qualitative validation on left eyes.

To qualitatively assess the segmentation performance for both eyes, a subset of 10 T1w images was randomly selected from the full cohort (N = 1210). For each subject, the left-eye, right-eye, and merged (left–right) segmentations produced by the model were visually evaluated by one of our ophthalmologist coauthors (AKL). Each segmented structure (lens, globe, optic nerve, intraconal fat, extraconal fat, superior rectus, inferior rectus, lateral rectus, and medial rectus) was scored using a 5-point ordinal scale: 0 = exclude, 1 = poor, 2 = acceptable, 3 = good, and 4 = excellent. The reviewer was also invited to provide comments regarding segmentation quality or potential artifacts. The evaluation focused on anatomical plausibility, boundary accuracy, and consistency with the underlying MRI appearance.

### Biomarkers extraction

#### Metadata.

We extracted metadata (sex, age, height, weight) from the original DICOM files and computed BMI (kg/m^2^) per subject.

#### Axial length.

**Fig 8:**
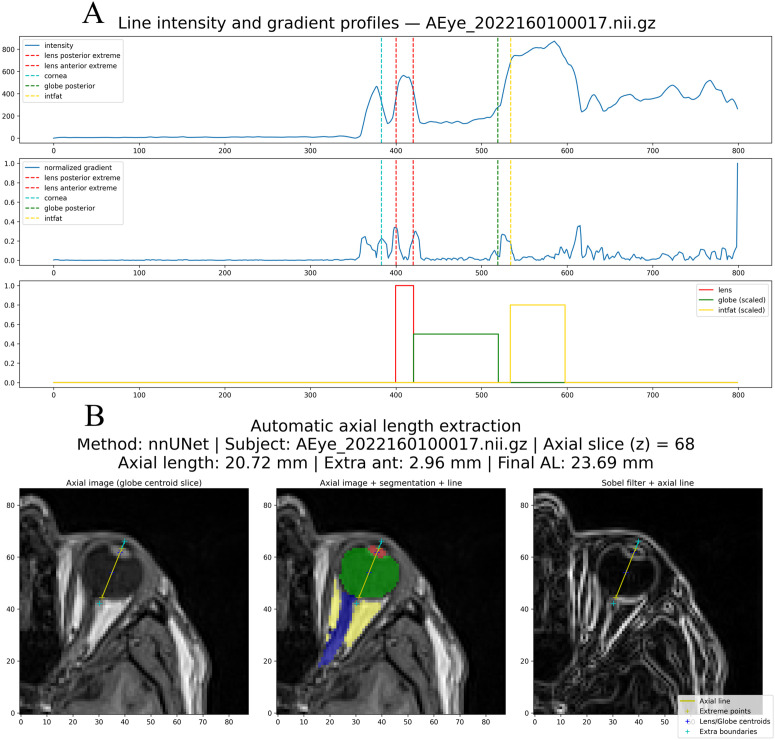
Example of AL extraction in T1w MRI. A) The intensity and gradient profiles of the line crossing the image. The intersection points of the line with the different structures are shown in the plot with different colors. The cornea is detected as the second brightness peak in the gradient profile. B) Different representations of the automatic extraction using the segmented structures and the T1w image; on the right, gradient image visual aid. The cornea, in the gradient image, can be seen as the bright area between the eyelid and the lens. The selected axial slice corresponds to the centroid of the globe, and the line and other intersection points are projected onto this slice.

We developed an algorithm to automatically extract the AL, defined in [40] as the distance from the posterior surface of the cornea to the posterior pole of the ocular bulb, at the boundary with orbital fat (the image had to include the corneal apex as well as the optic nerve head), and illustrated in Fig 8. The method inputs both the automated segmented labels and T1w images. First, we determine the line connecting the centroids of the lens and the globe and identify its extreme intersection points with these segmented structures. To estimate the anterior corneal boundary—since manual cornea segmentation is unavailable—we analyze the intensity gradient along the same line. The first peak typically corresponds to the eyelid, and the second to the cornea (the third to the lens) in images with eyes closed. When the cornea could not be detected (147/1210 ≈ 12.1% of right eyes and 104/1210 ≈ 8.6% of left eyes), the anterior corneal distance was defined as the median value observed in subjects with eyes closed. Based on visual inspection of representative failure cases, these non-detections were mainly associated with limited visibility of the anterior eye region in the T1w images, for example when the eyes were open or not fully closed, when the lens was poorly visible or missing, or when image quality around the lens–cornea transition was insufficient for reliable boundary identification. This reflects the limited MRI visibility of the cornea, which is a thin structure (~0.55 mm) below the spatial resolution of the images used in this study, rather than instability of the segmentation model itself. The total axial length is then defined as the distance from the cornea to the posterior pole of the globe.

#### Volumetry.

The volumetry of the different segmented eye structures in mm^3^ was estimated based on the number of voxels per structure, each voxel of 1 mm^3^.

#### Correlation between volumetry and BMI.

We fit the volumes and BMIs per structure through a Huber regressor, a linear model robust to outliers. We used scikit-learn library (version 1.1.2). We obtained the slope, the intercept, and the R^2^ score.

### Atlas of the eye

**Fig 9:**
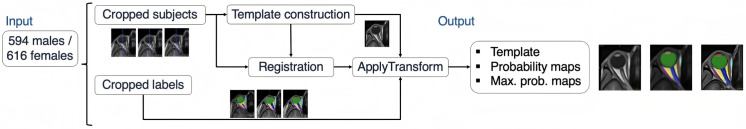
Scheme of unbiased template construction of the MR-Eye atlases and generation of the labels. The cropped images serve to construct the template, which then is registered to the individuals’ images to transpose their labels into its space.

Fig 9 presents the block diagram for the development of this section.

#### Template construction.

We performed metric-based registration, consisting of rigid, affine, and then deformable registration, with ANTs toolkit [98,99] to iteratively create an average mapping of the subjects grouped by sex (594 males and 616 females). We made use of the multivariate template construction tool, using as input images the right-eye-cropped ones obtained from the segmentation method (nn-UNet). Therefore, they were much smaller than the initial ones (that included the whole head). The maximum size of these right-eye-cropped images for the three axes was 61 x 70 x 68 and 77 x 95 x 94 voxels for the male and female case, respectively, and the size of the original images was 176 x 256 x 176 voxels. The size of the voxels remained 1mm^3^. For the deformable registration, we chose the SyN registration algorithm with the similarity metric of cross-correlation. We chose four resolution levels (8, 4, 2, 1), and iterated over each level for 80, 60, 40, and 10 iterations, respectively. Considering the reduced size of the images, we set the iteration limit (the number of iterations of the template construction) to 15, as we wanted to allow enough iterations for the template to converge and capture the variations present in our dataset. We used an 11th Gen Intel® Core™ i9-11900K × 16 processor with 64GB of RAM. The time spent to construct both atlases was 16h 15m 45s and 32h 16m 45s for the male and female cases, respectively.

#### Labels generation.

To generate the atlas labels, we first registered each subject to the corresponding atlas space (male or female) and projected the nnU-Net segmentations accordingly. The overall process required approximately 25 minutes for the male cohort and 39 minutes for the female cohort. We then constructed a maximum-probability atlas using majority voting across subjects. In addition, we explicitly represented uncertainty in the atlas labels through probability maps, which we provide alongside the atlas. These maps encode the voxel-wise frequency of label occurrence across the cohort after spatial normalization, thereby reflecting the degree of consensus (or uncertainty) for each anatomical structure. This probabilistic representation complements the maximum-probability labels and allows identification of regions with higher uncertainty, such as diffuse anatomical boundaries (e.g., extraconal fat). For visualization purposes, we color-coded the probability maps by modulating label-specific intensities according to voxel-wise probabilities, such that lower-probability regions appear less saturated. The eye atlases can be downloaded at [59].

#### Registration to common VCS.

We first cropped the eye region of the templates [50,51] using their right-eye masks that we extracted by a modified version of the antsBrainExtraction. Then, we registered them to the combined eye atlas, projected its labels onto the cropped spaces, and finally transposed them back into the original spaces (inverse cropping).

#### Atlas validation.

To evaluate the reliability of the atlas labels, the combined atlas was manually reviewed and segmented by one of our ophthalmologist coauthors (AKL). Similarity metrics between the manual atlas segmentation and the automatically generated atlas labels were computed for each structure using DSC, HD and VD.

### Quality control protocol

**Fig 10:**
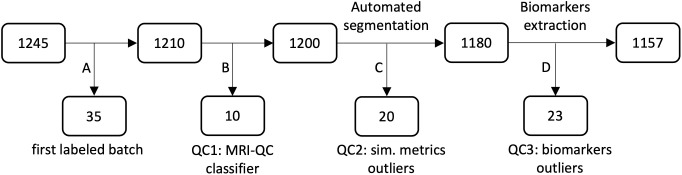
QA/QC integration within a simplified scheme of the A-Eye project’s pipeline. **(A)** The first batch of 35 manually annotated subjects are removed from the QC protocol as they all have included quality. **(B)** Subjects excluded from MRIQC classifier. **(C)** Subjects excluded from similarity metrics outliers between nnU-Net and the baseline [76] segmentation results. **(D)** Subjects excluded from biomarkers outliers (AL and volumetry). In total, 53 subjects were excluded because of their image quality for our application, with 1157 subjects remaining.

Fig. 10 shows a block diagram of this quality control process throughout the pipeline. We passed QC checks at different points of the pipeline (described below) to capture possible excluded-quality subjects, and then manually review those cases, using the previously mentioned reports, to ensure which of them were really excluded. The exclusion criteria for our application are twofold: first, the quality of the image must be acceptable in terms of noise, blur, motion, and not include heavy artifacts on the area of evaluation (the eyes); and second, all structures intended for segmentation must be visible (i.e., if an image presents no visible lens, it would be removed). We did not follow further inclusion/exclusion criteria presented in [40], such as including only the images in which the corneal apex and the head of the optic nerve were in the same axial plane or excluding images where there was a lateral deviation of the subject’s viewing direction. Their application [40] was focused on imaging analysis (AL and exophthalmos) whereas ours was mostly focused on image segmentation (followed by imaging analysis).

The QA/QC checks we performed were:

1. Before image segmentation: we ran MRIQC (38), to extract no-reference IQMs, and MRIQC classifier, trained and tested on ABIDE and DS030 datasets, respectively, with updated scikit-learn and NumPy Python libraries, to extract candidates as possible excluded-quality images. From 1210 subjects (the first batch of 35 manually annotated subjects was not included in the QA/QC protocols, as they had included quality to be manually segmented in the first place), 29 were flagged by the classifier for exclusion, and, after manual review, 10 were ultimately excluded regarding our criteria.
2. After segmentation: we computed the already mentioned similarity metrics but this time between the results of the nnU-Net and the baseline (atlas-based) [76] methods, to then extract the outliers using the interquartile approach, as the sets do not follow a normal distribution. The values below and above the lower (Q1-1.5*IQR) and upper (Q3 + 1.5*IQR) bounds, respectively, were selected as outliers. In total we had 102 outliers, which we manually reviewed, and excluded 20 of them, regarding our criteria.
3. After biomarker extraction: we extracted the outliers following the same method as before in both AL and volumetry cases. From AL, there were 45 and 150 outliers for atlas-based and nnU-Net methods, respectively, some of them shared between the two. After manual revision, 21 were excluded in total. From volumetry, 25 and 53 subjects popped up as outliers for atlas-based and nnU-Net methods, respectively. Again, some of them were shared between the two. After manual revision, only 2 subjects were excluded. In total, in this third step, we removed 23 subjects. The nnU-Net method produced more outliers, particularly for AL, because when the lens is not visible in the T1w image, the model cannot segment it, resulting in an AL value of zero. In contrast, the atlas-based method always includes a lens, even if it is not visible in the original image, since it relies on image registration where the reference atlas contains a lens that is transposed to the subject. For volumetry, it follows the same reasoning, the atlas-based method would always transpose the structures, unlike the DL method, which could sometimes fail to even segment a single voxel of a specific structure (i.e., the lens).

In total, 53/1210 subjects (4.38%) were excluded, leaving 1,157 quality-controlled subjects.

### Declaration of generative AI and AI-assisted technologies

We used generative AI to create code segments based on task descriptions, as well as to debug, edit, and autocomplete code. Additionally, generative AI technologies have been employed to assist in structuring sentences and performing grammatical checks. The conceptualization, ideation, and all prompts provided to the AI originate entirely from the authors’ creative and intellectual efforts. We take accountability for the review of all content generated by AI in this work.

## Supporting information

S1 Fig Pairwise correlations between segmentation metrics across regions. Heatmaps show Pearson correlations between DSC (3D overlap, higher is better), HD (boundary distance, lower is better), and VD (volume difference, closer to 0 is better). Negative DSC–HD and DSC–VD correlations indicate that better overlap corresponds to better contour and volume agreement, while positive HD–VD correlations indicate that larger boundary errors are associated with larger volume differences. Weaker correlations are found in the optic nerve and rectus muscles, probably due to their variable shape across subjects. All correlations are significant (p < 0.05).
(TIF)

S2 Fig Subjective ratings and DSC agreement for N = 43 non-excluded subjects. In each plot, the x-axis represents the subjective rating (0 = excluded, 4 = excellent), and the y-axis represents the DSC. The average DSC plot shows no clear monotonic relationship between subjective image quality and segmentation performance (low correlation). Scatter plots for individual structures are also shown, with greater variability observed in the fat compartments, particularly the extraconal fat, likely reflecting their higher anatomical variability in shape and size.
(TIF)

S3 Table Unadjusted comparisons between males and females for orbital structure volumes. Mean difference is reported as male minus female.
(CSV)

S4 Table Adjusted regression analysis of sex differences in orbital volumes, including body height and age as covariates. Sex coefficient (βsex) represents the effect of male relative to female.
(CSV)

S5 Table Adjusted robust regression results for BMI and orbital volumes. (CSV)

S6 Table Pearson correlation between BMI and orbital structure volumes. (CSV)

S7 Table Bilateral consistency analysis of orbital structures. For each structure, Pearson correlation (r), agreement metrics (mean difference, mean absolute difference, and relative asymmetry), and paired statistical tests are reported. A permutation analysis (shuffled left–right eyes pairing) was performed for total orbital volume to assess whether the observed correlation reflects true within-subject correspondence.
(CSV)

S8 Table Qualitative evaluation of left-eye segmentation quality. Mean qualitative scores (0–4 scale) assigned by an ophthalmologist across 10 subjects for each segmented structure. Higher values indicate better segmentation quality; extraconal fat showed the lowest average score due to its diffuse MRI boundaries.
(XLSX)

S9 Table Similarity metrics between manual atlas segmentation and automatically generated atlas labels. DSC: Dice Similarity Coefficient, HD: average Hausdorff distance, VD: volume difference. Differences reflect variations in annotation protocol between atlas and subject-level manual segmentations.
(CSV)

S10 File MR-Eye Quality Control Annotation Guidelines. Guidelines used by raters for the subjective quality control (QC) evaluation of eye MRI images, including rating criteria, artefact assessment workflow, and instructions for assigning overall image quality scores.
(DOCX)

S11 Fig Training and validation loss curves (blue and red, respectively) and validation Dice score (green dashed line) across epochs for nnU-Net training. The curves show stable convergence, with decreasing loss and consistent improvement of the Dice score, indicating no evident overfitting.
(TIF)

S12 File Supplementary materials. The zip file contains results from the study.
(ZIP)
