# Supplementary figures and images for: A-eye: Automated 3D MRI segmentation and morphometric feature extraction for eye and orbit atlas construction
Source: PLoS One. 2026 Jul 2;21(7):e0352257. doi: 10.1371/journal.pone.0352257 (PMC13327317; doi:10.1371/journal.pone.0352257)

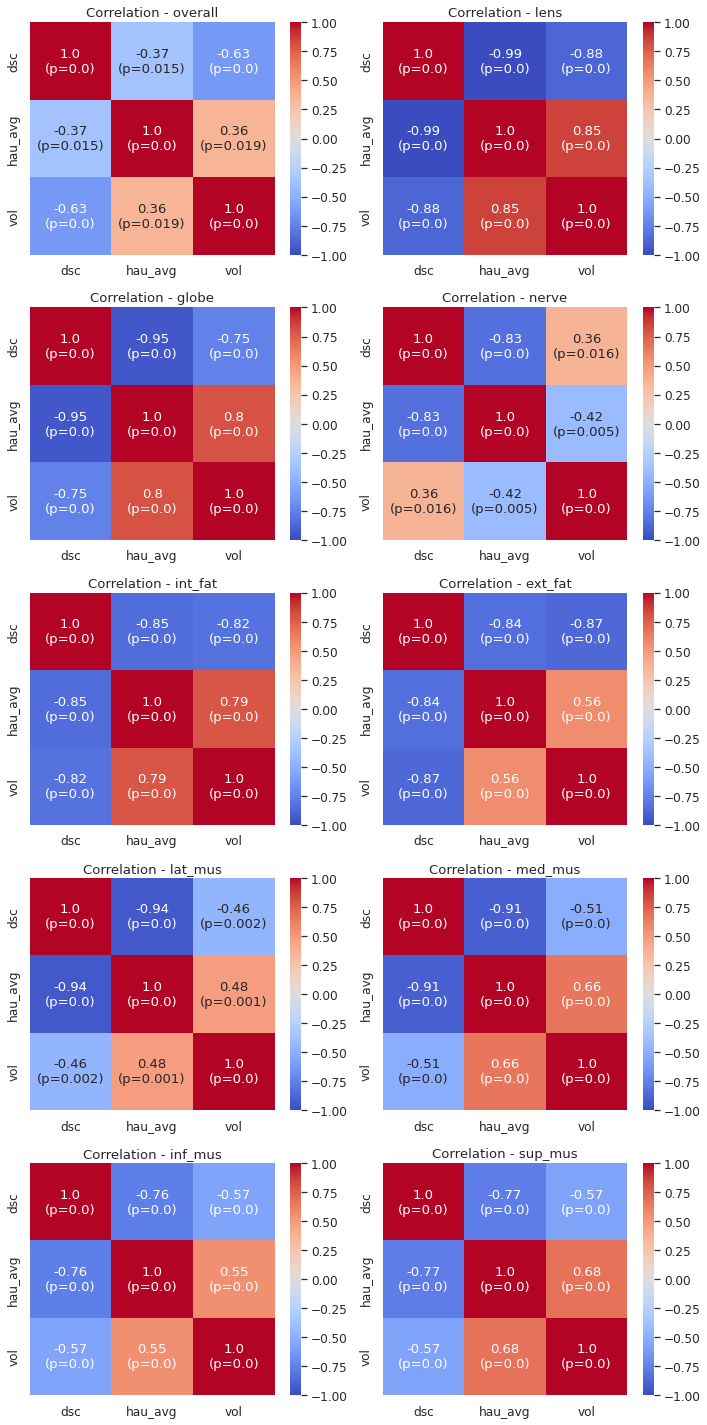

Supplement: S1 Fig — Heatmaps show Pearson correlations between DSC (3D overlap, higher is better), HD (boundary distance, lower is better), and VD (volume difference, closer to 0 is better). Negative DSC–HD and DSC–VD correlations indicate that better overlap corresponds to better contour and volume agreement, while positive HD–VD correlations indicate that larger boundary errors are associated with larger volume differences. Weaker correlations are found in the optic nerve and rectus muscles, probably due to their variable shape across subjects. All correlations are significant (p < 0.05). (TIF) [file pone.0352257.s001.tif]

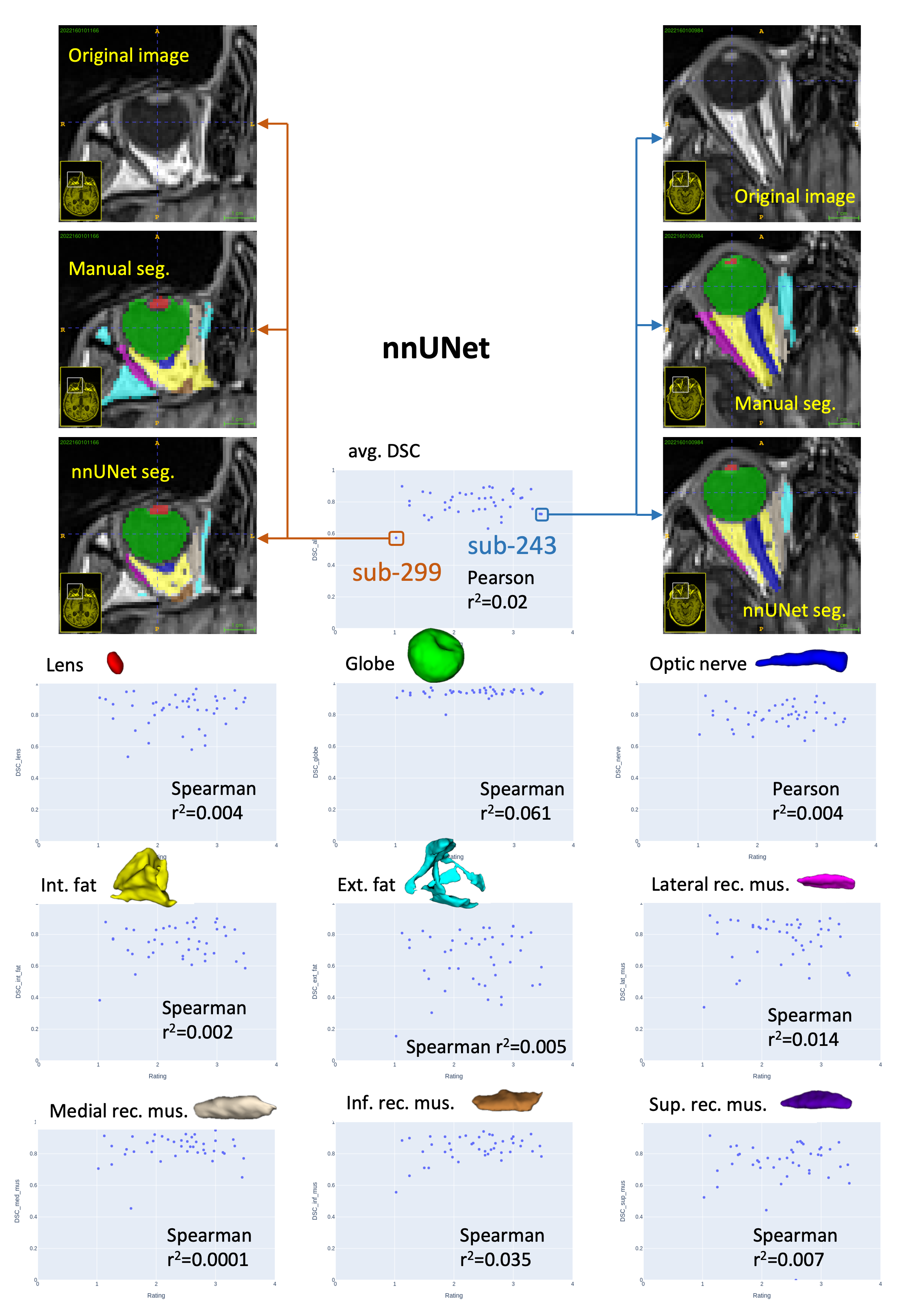

Supplement: S2 Fig — In each plot, the x-axis represents the subjective rating (0 = excluded, 4 = excellent), and the y-axis represents the DSC. The average DSC plot shows no clear monotonic relationship between subjective image quality and segmentation performance (low correlation). Scatter plots for individual structures are also shown, with greater variability observed in the fat compartments, particularly the extraconal fat, likely reflecting their higher anatomical variability in shape and size. (TIF) [file pone.0352257.s002.tif]

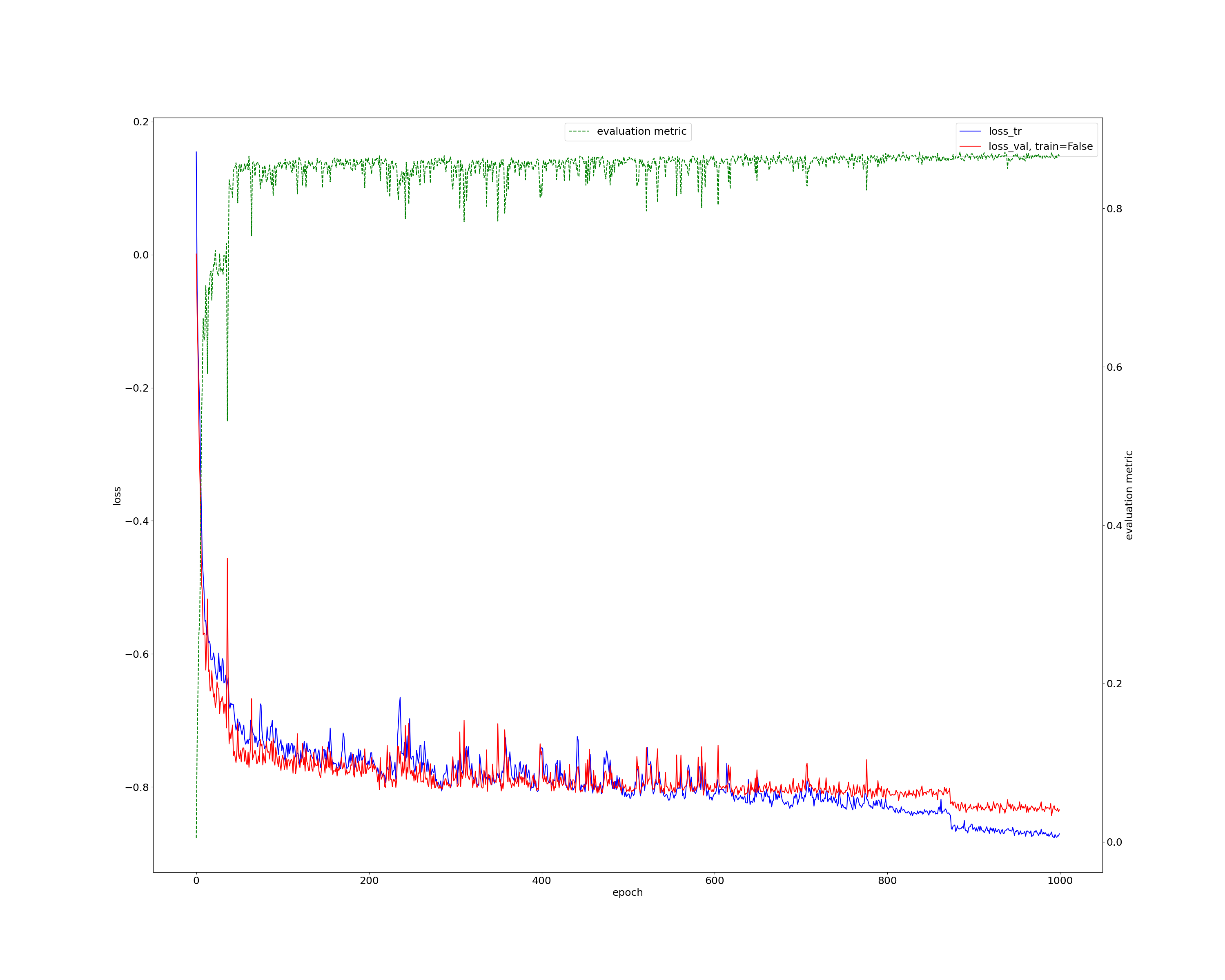

Supplement: S11 Fig — The curves show stable convergence, with decreasing loss and consistent improvement of the Dice score, indicating no evident overfitting. (TIF) [file pone.0352257.s011.tif]

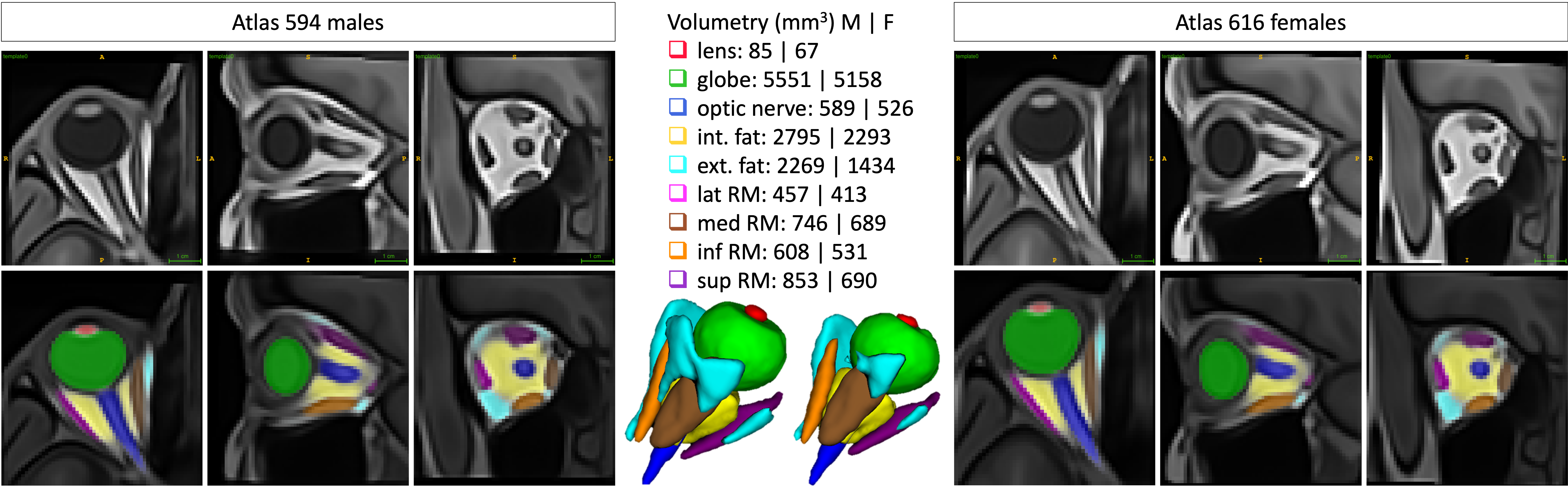

Supplement: S12 File — The zip file contains results from the study. (ZIP) [file pone.0352257.s012.zip › S12_file/eye_atlases/A_eye_atlases_male_female.png]

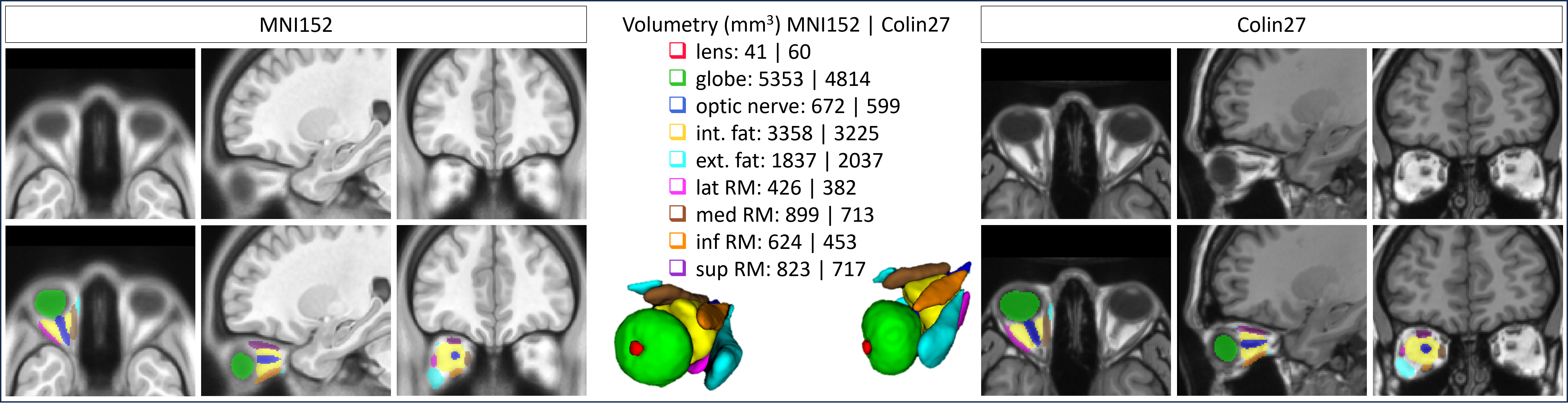

Supplement: S12 File — The zip file contains results from the study. (ZIP) [file pone.0352257.s012.zip › S12_file/eye_atlases/A_mni_colin_eye_labels.png]

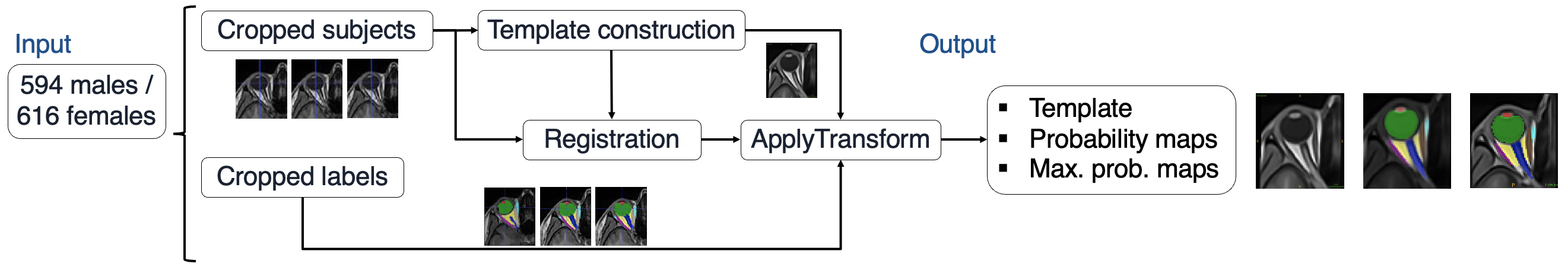

Supplement: S12 File — The zip file contains results from the study. (ZIP) [file pone.0352257.s012.zip › S12_file/eye_atlases/A_scheme.png]
