# Supplementary material for: A-eye: Automated 3D MRI segmentation and morphometric feature extraction for eye and orbit atlas construction
Source: PLoS One. 2026 Jul 2;21(7):e0352257. doi: 10.1371/journal.pone.0352257 (PMC13327317; doi:10.1371/journal.pone.0352257)
Supplement: S10 File — Guidelines used by raters for the subjective quality control (QC) evaluation of eye MRI images, including rating criteria, artefact assessment workflow, and instructions for assigning overall image quality scores. (DOCX) [file pone.0352257.s010.docx]

CIBM.CH

Meritxell Bach Cuadra, Benedetta Franceschiello and Jaime Barranco

CIBM SP CHUV-UNIL, Department of Radiology

Eye MRI Quality Annotation Guidelines

Lausanne, 25/11/24

1. Research context

These guidelines are written in the context of the **Gelbert Foundation project** named **A-eye**. Our project aims at developing machine learning methods for the study of large-scale ocular Magnetic Resonance Images, with the goal to provide researchers with a web-platform where the developed image analysis methods (eye and orbit structures’ segmentation and biomarkers extraction) are integrated.

Quality control (QC) of input images is a crucial step in any image analysis pipeline. It is very well known that **bad quality data can strongly bias the extracted imaging biomarkers**. In the context of our A-eye web-platform QC is also very important to double-check prior to any analysis the minimum input quality for the predictions. However, existing MR quality tools neglect the eye area. In fact, the eye is often removed from the image to analyze its quality. Furthermore, head or brain image quality cannot always directly be extrapolated to the eye area (1).

The guidelines here below are strongly inspired from previous works on fetal and adult brain MRI quality control (2, 3).

1. General remarks

We aim at **standardizing the QC manual annotation** procedure (as to reduce inter-rater variability) for the evaluation of QC **in eye MRI, that is, the eye and orbit structures, namely lens, globe, optic nerve, and rectus muscles**.

The general question in mind while annotating image quality is: **can I manually delineate with confidence ALL the structures of the eye?** We may have a tendency in evaluating the quality of the image based on the lens and globe, but as we’re navigating a new field, we need to treat every single structure as separate and report an average of all structures in the end.

We suggest **considering two different macro areas**:

1) the external part of the orbit (globe and lens) and,

2) the internal part of the orbit, composed of the optic nerve and muscles.

We need to explore images as 3D volumes, observing quality as visible in the three different Axial, Sagittal and Coronal views. We should not penalize the image rating if the optic nerve and the lens are not onto the same plane as our automated tools will work on the 3D volume, not on 2D planes.

We suggest **rating specific image artefacts first** (e.g. blur, motion, bias, etc.) and **at the end only provide the global rating**.

1. Annotation reports

Manual annotations are executed through a browser using html widget adopted from (3).

Some further comments:

- Use Chrome preferably (Safari has some bugs).
- Load index.html.
- Scroll all thumbnail images, note that sagittal and coronal views are at the bottom.
- You can select thumbnails (red square appear) to indicate heavy artefact is present.
- You can then turn on the rating widget (top right).
- You can proceed with your evaluation; first rate each artefact considering the ensemble of orbit structures and then rate the global metric.
- When opening the widget, if you don’t visualize it properly or it is too big or too small, you can change the zoom of the webpage. The images will remain the same size.
- Download report (ensure you know where they are saved).
- If you plan on doing the evaluation not all at once, it’s better to save the progress by clicking on “Save progress config” at “Home” page when you finish the subset -- This will download a json file that you can later upload to the “Home” page by clicking on “Choose File” button to recover the progress (those in green are done).
- If you forgot to save the progress, you could always check your last downloaded json file name.

1. Specific image artefacts

Blurring

We observe blurred contours of the main eye structures, along the lens, globe and muscles.


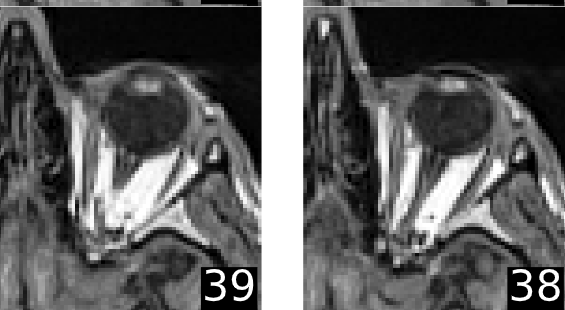


Noise

Grey intensity texture appearing inside the eye globe, as well in the brain tissues (white matter). When strong noise it looks like salt/pepper appearance.


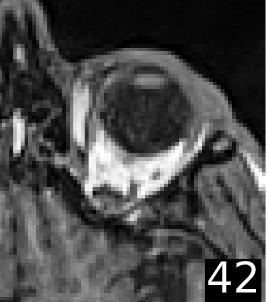


Motion

It can appear as disrupted geometry of basic shapes (meaning that spherical, elliptical forms are not maintained, as for instance in the vitreous of the example below). It can also appear in the form of “lines” (horizontal), due to recon artefacts due to head motion.


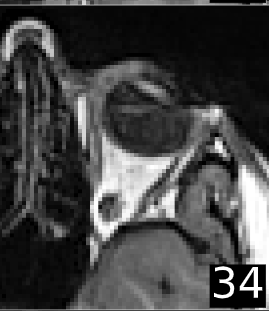


Background/Air Artifact

We can observe light gray bands crossing horizontally the front part of the head across the eye. In the case below, we observe also noise in the background. This can occur both in eyes open and closed.


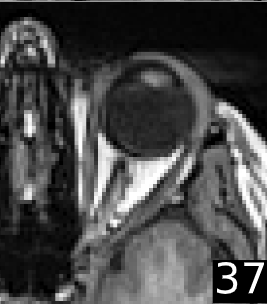


This image also contains noise and blurry contours on the right side.

Eyes open/closed - This is not an artefact per se, but we would like to annotate if the eye is closed or open

In the example below, left eye is open, right eye is closed. We can observe that the eyelid is imaged on the right, and it appears as a light grey cover of the lens. The Left picture shows blurring, air artefacts and it appears more noisy than right eye.


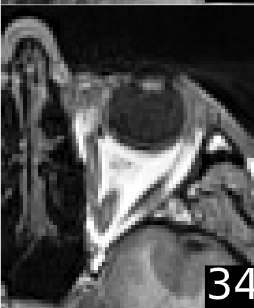

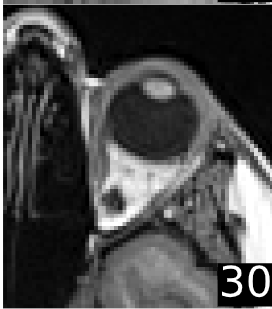


1. Examples of quality ratings

Bad quality (Poor at the border of exclusion)

- This eye presents the lens in a very blurry way, there is also quite a lot of noise within the vitreous.
- I would attribute a poor quality at the limit of exclusion, I suspect the lens cannot be well segmented, still I am not excluding it.


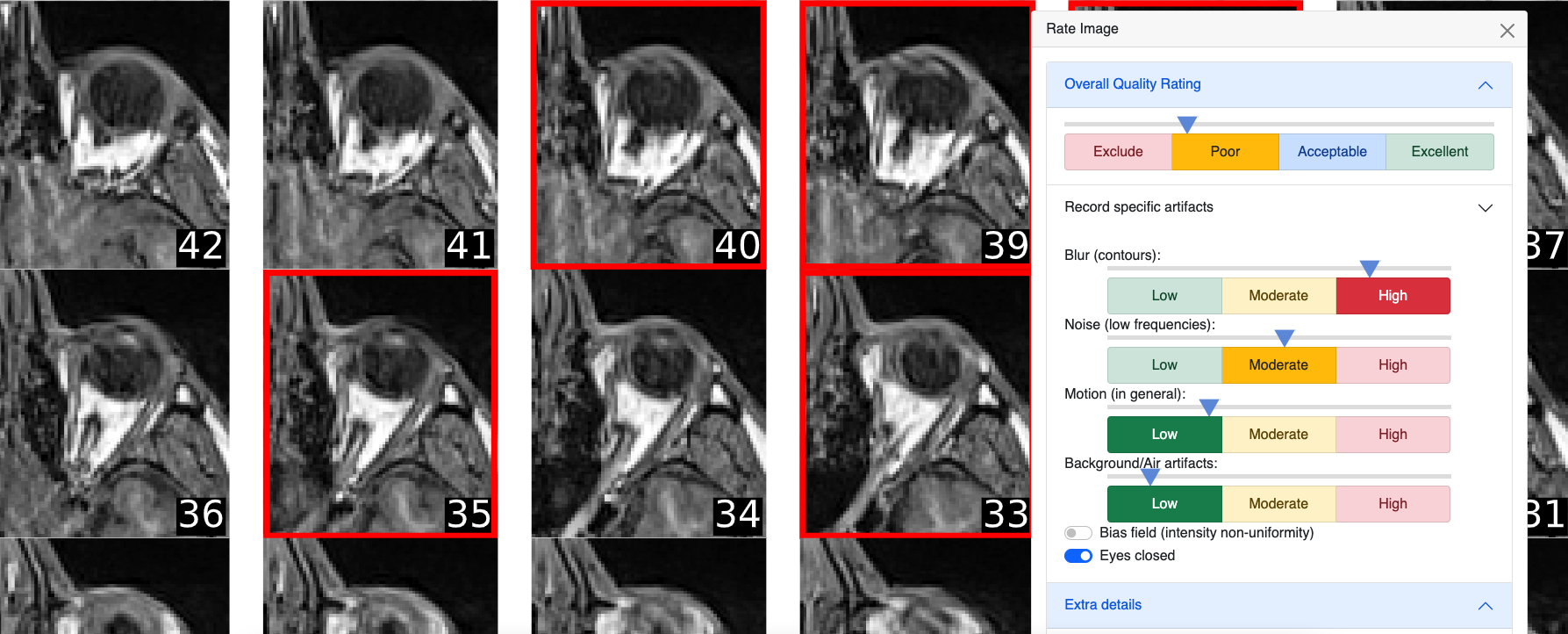


Bad quality (Poor)

- The eye presents blur.
- The eye presents motion (geometrical distortion), particularly in slice 34-32
- The eye presents noise. No air artefacts. Shapes are not easy to identify


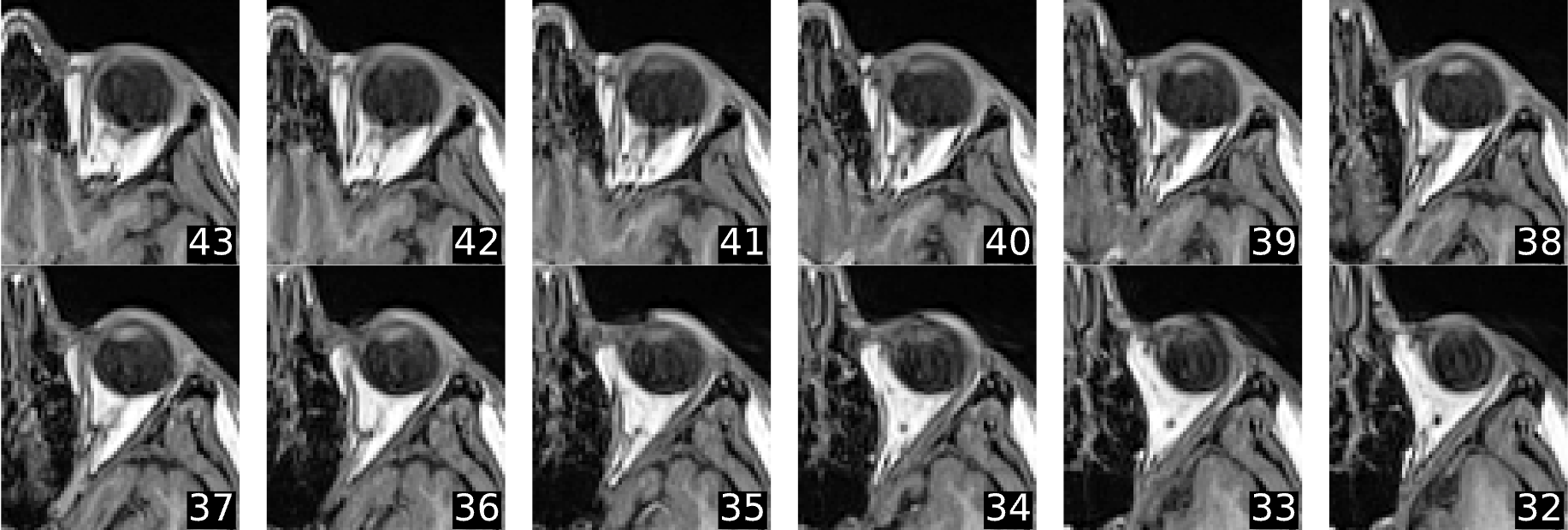


Bad quality (Middle Poor):

- This image is distorted by motion artifacts, air artifacts, noise.
- We can still somehow distinguish the structures, so we could in principle manually segment the eye substructures.
- The combination of 1) and 2) make us include the image, despite its poor quality.


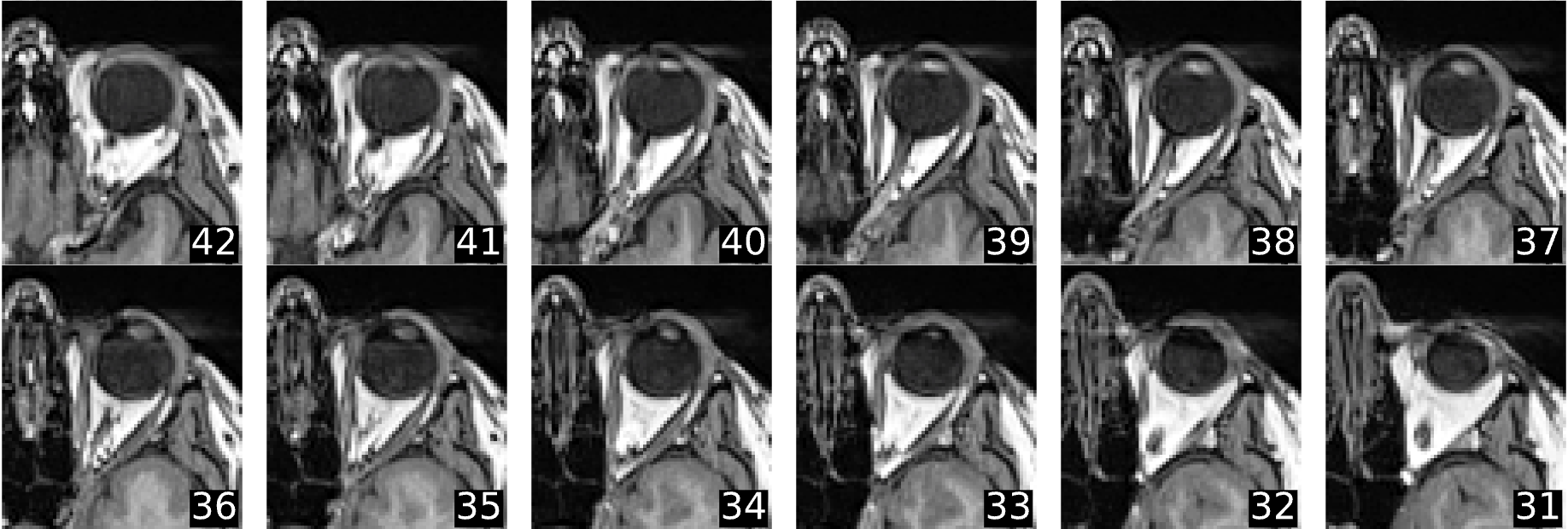


Bad quality (Poor towards acceptable)

- This eye presents the lens not so blurry as the previous but still contours are very blurry, there is also quite a lot of noise within the vitreous.
- This image shows more profoundly the background/air artifacts (horizontal band)
- It seems eyes are open for this subject.


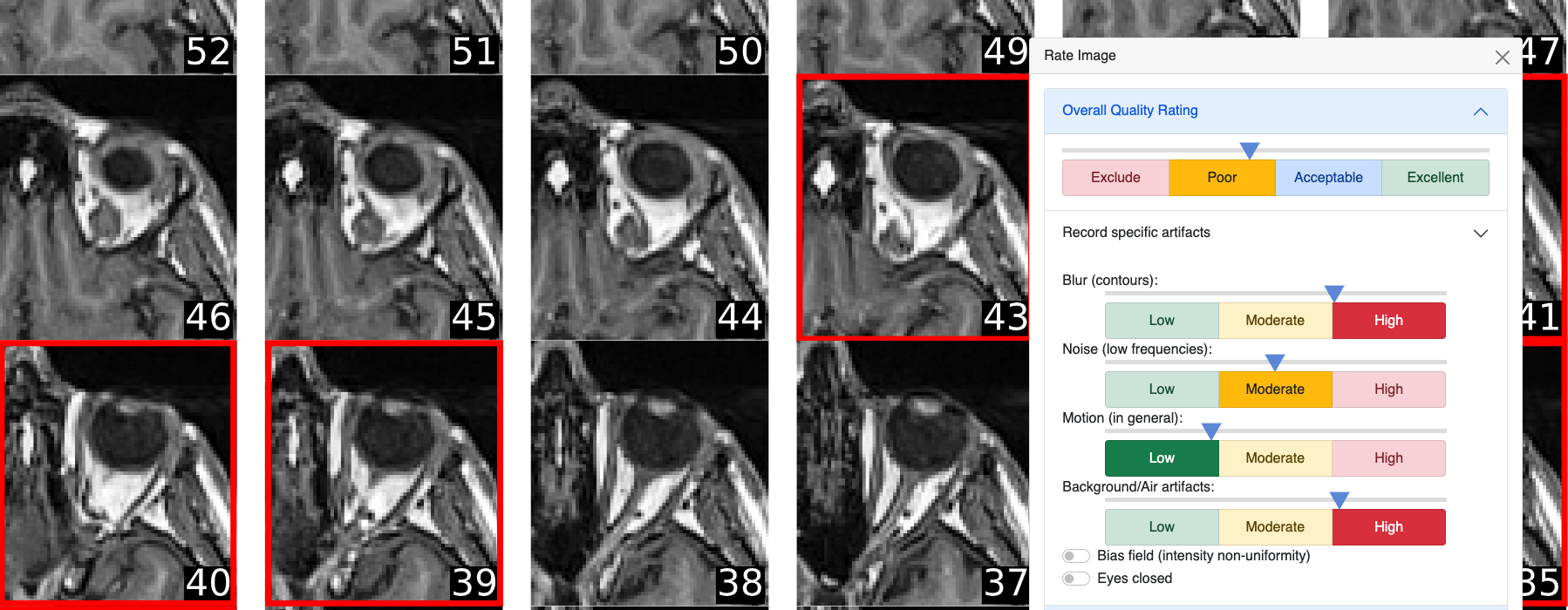


Average quality (Acceptable)

- We can distinguish the eye structures, but we observe background noise and blurring in the contours.
- Slight air artefacts are present (slides 41,40)
- Presence of motion (slide 42,43), subject has probably the eyes closed.


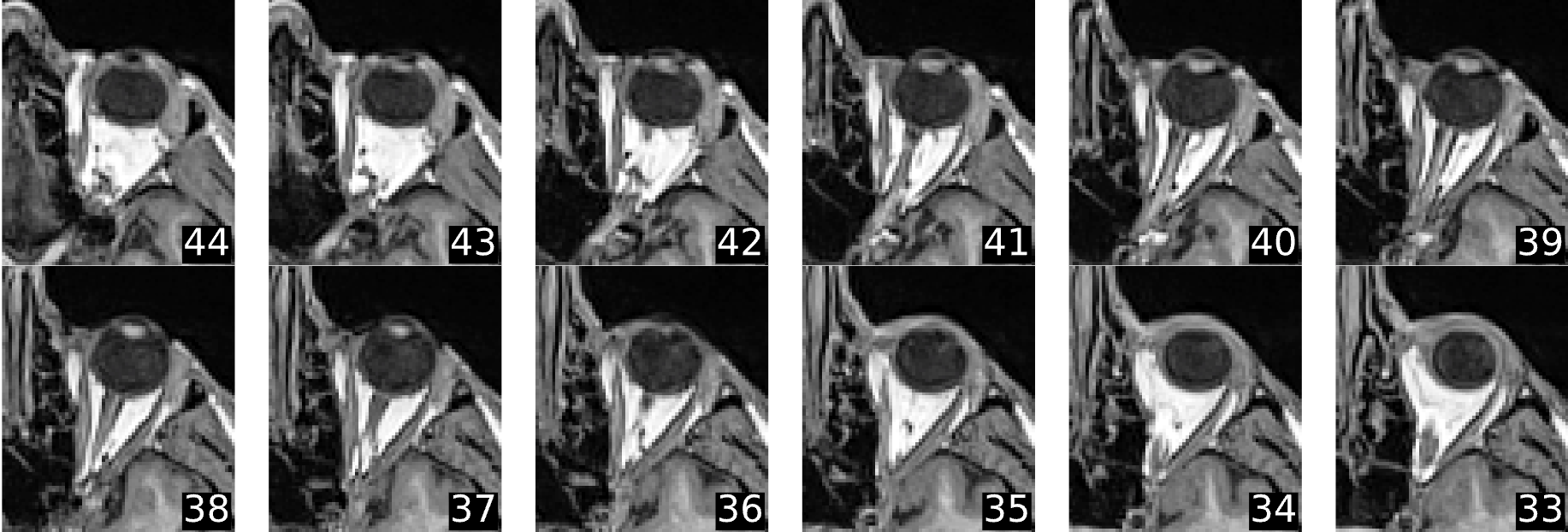


Good quality (Acceptable up)

- The eyes present the structure in an ok sharp manner.
- We observe the presence of background noise, and some air artefacts.
- Eyes seem closed for this subject.


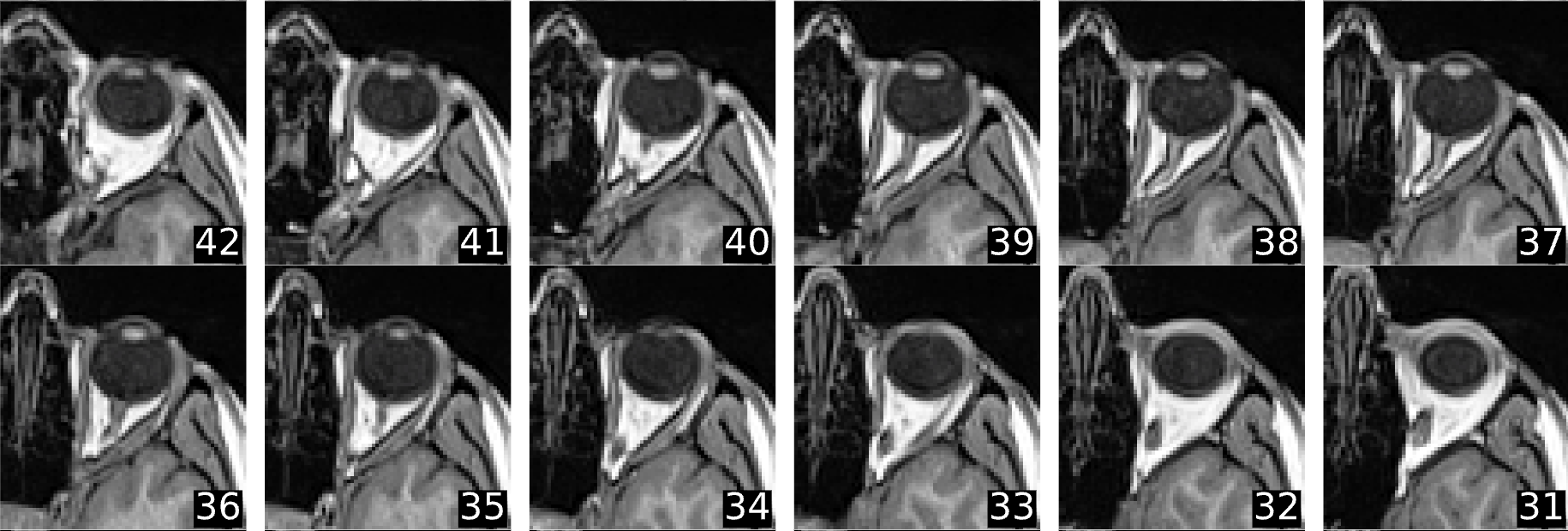


Good quality (Acceptable towards Excellent)

- This eye presents the lens in a very sharp manner
- This image shows slightly a background noise, but some weird noisy stuff within the vitreous combined with very dark areas in the vitreous.
- It seems eyes are open for this subject.


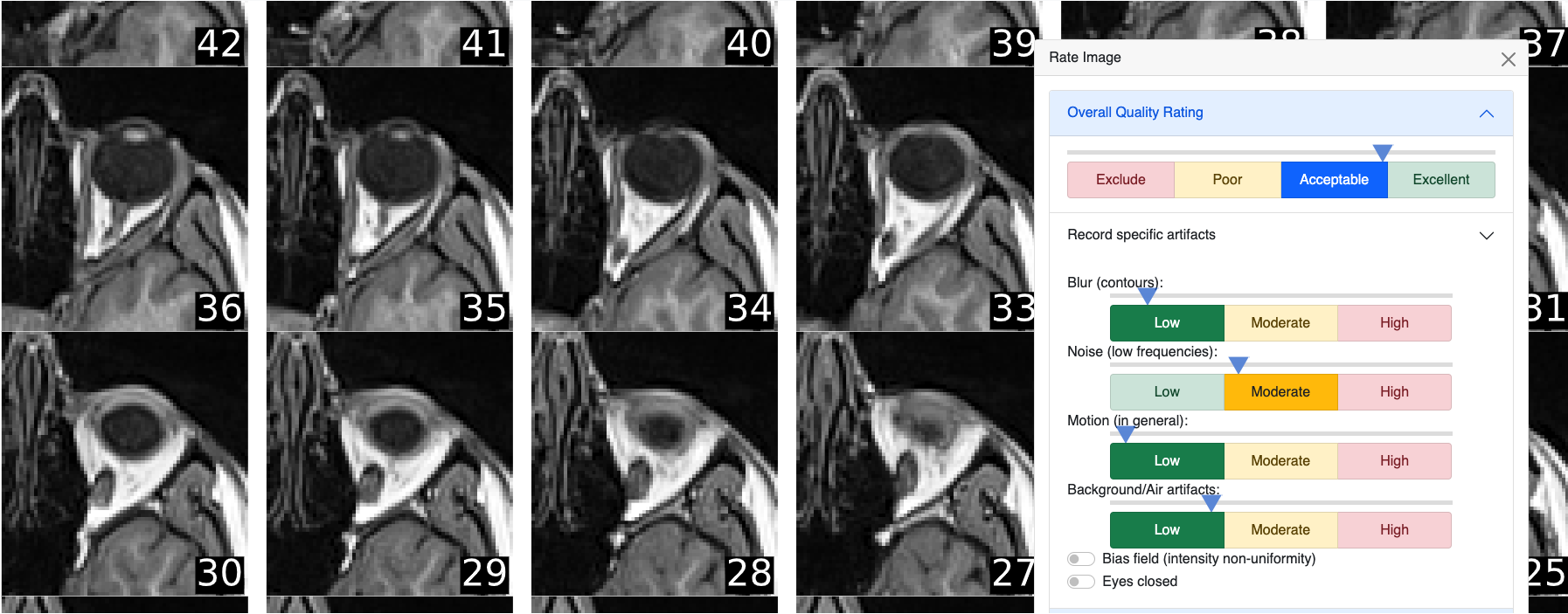


Excellent quality

- This eye presents the eye structures in a very sharp manner
- There is no background artifact, but still mild heterogeneity within the vitreous (background noise)
- It seems eyes are closed for this subject
- Unfortunately, the optic nerve head is not in the same plane as the lens. We identify this as a potential problem for automated axial length extraction.


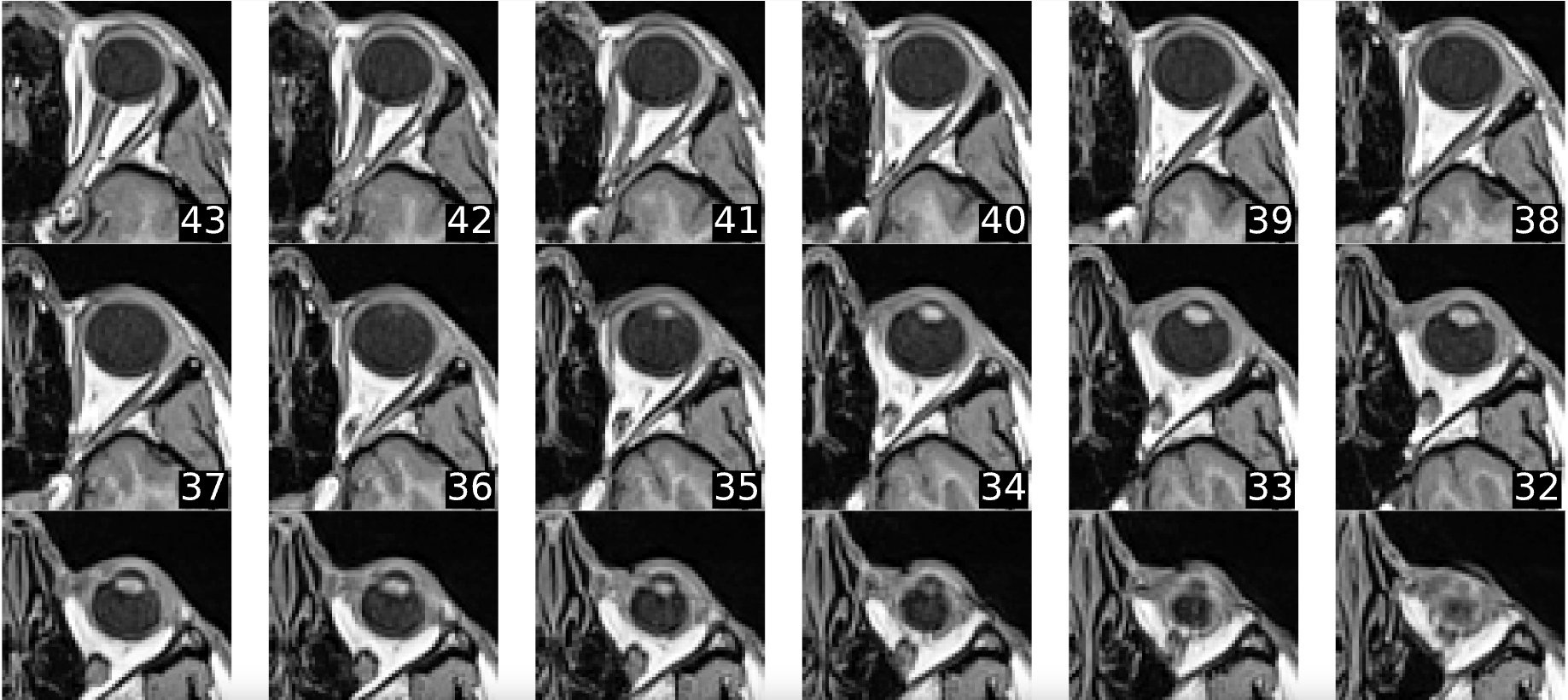


Important example: internal orbital structures (muscles, nerves) are fine, whereas external structures (lens, globe) are deformed by motion.


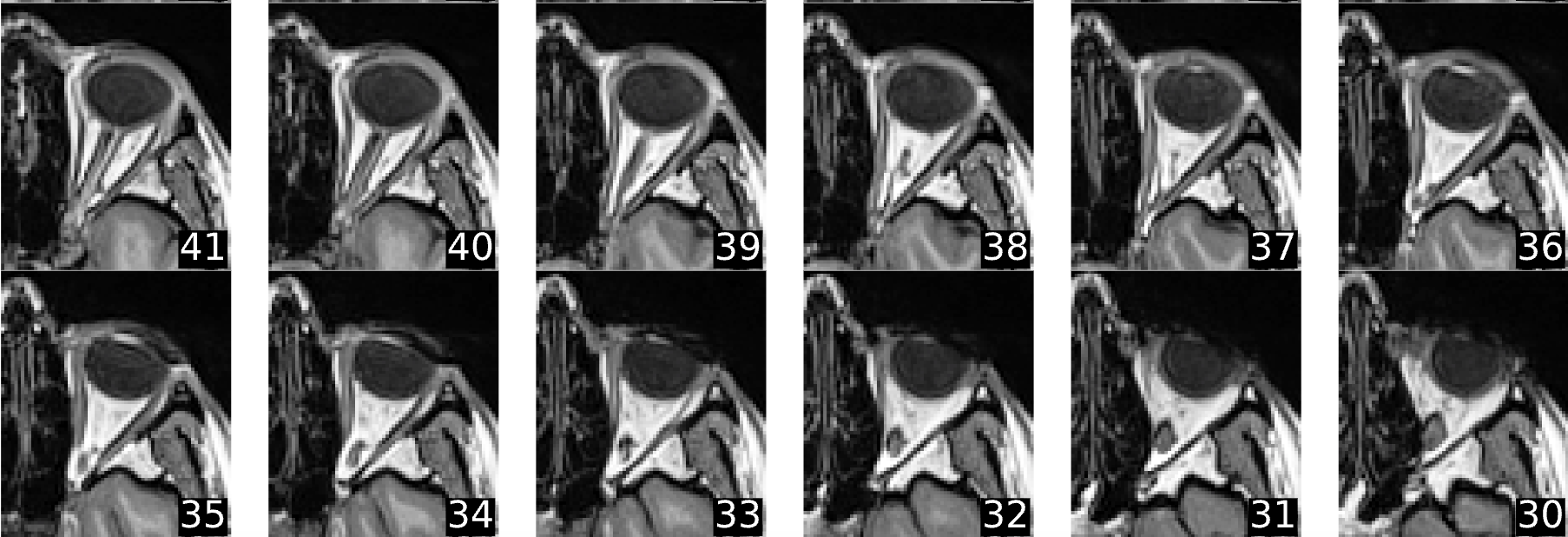


1. References
2. Barranco J., Kebiri H., Esteban O., Sznitman R., Stachs O., Stachs P., Langner S., Franceschiello B., Bach Cuadra M., A-Eye: quality control and deep learning segmentation of the complete eye in MRI, ISMRM abstract (2024).
3. Sanchez, T., Esteban, O., Gomez, Y., Pron, A., Koob, M., Dunet, V., Girard, N., Jakab, A., Eixarch, E., Auzias, G., & Bach Cuadra, M. (2024). FetMRQC: A robust quality control system for multi-centric fetal brain MRI. *Medical Image Analysis*, *97*, 103282. https://doi.org/10.1016/j.media.2024.103282
4. Esteban, O., Birman, D., Schaer, M., Koyejo, O. O., Poldrack, R. A., & Gorgolewski, K. J. (2017). MRIQC: Advancing the automatic prediction of image quality in MRI from unseen sites. *PLOS ONE*, *12*(9), e0184661. https://doi.org/10.1371/journal.pone.0184661
