# Supplementary material for: A-eye: Automated 3D MRI segmentation and morphometric feature extraction for eye and orbit atlas construction
Source: PLoS One. 2026 Jul 2;21(7):e0352257. doi: 10.1371/journal.pone.0352257 (PMC13327317; doi:10.1371/journal.pone.0352257)
Supplement: Attachment — Submitted filename: Rebuttal_letter.pdf [file pone.0352257.s014.pdf]

Authors’ Response to Reviews of

J. Barranco et al. – “A-eye: Automated 3D MRI Segmentation and Morphometric Feature Extraction for Eye and Orbit Atlas Construction”

AR: Authors’ Response

We thank the reviewers for their thoughtful comments. We are pleased to submit a revised version of the manuscript, having considered all the received feedback.

In this response, we reproduce all changes made to our submission, marked up with the *Edit* keyword. Page and line numbers of the “Original” text cross-reference the submitted version **without** markup of tracked changes. Page and line numbers of the “Revised” text cross-reference the new document **with** markup of tracked changes. For example:

Edit: **Original (p. N, ll. aaa–bbb):**

> Text in the ~~submitted~~ manuscript—version with~~out~~ track-changes markup,—with ~~deletions marked up by a strike-through line~~.

Revised (p. M, ll. yyy–zzz):

> Text in the revised manuscript—version with track-changes markup,—with additions marked up in blue-color text.

Reviewer #1

Comment #1:

In this study, the authors present an automated 3D segmentation method for MR images of the eye (MReye dataset). The method is based on a deep-learning approach (nnU-Net) to extract various regions of interest (ROIs) from MR images of 1245 subjects. A smaller subset of the data (n = 43 test + 31 train) was manually segmented by two human experts—a senior and a junior radiologist—and used to train and evaluate the performance of the deep learning model. The trained model was subsequently applied to segment the remainder of the dataset and generate a standard anatomical atlas of the eye across male and female subjects. The authors also demonstrate the method’s applicability in clinical settings by extracting a biomarker across the cohort.

This is a well-executed and well-written study with strong potential to contribute meaningfully to the field. While the segmentation model itself is not novel, its application to build a sex-specific anatomical eye atlas is both useful and timely. With the addition of some clarifications and statistical analysis, the paper will be significantly strengthened.

Inclusion of Sex-Based Atlas: The inclusion of a sex-specific atlas is a valuable contribution and

highlights an important dimension of anatomical variability that is often overlooked.

AR: We thank the reviewer for the thorough assessment and resonate with these general comments.

Comment #2:

Scientific Contribution and Novelty #1
While the study leverages an existing segmentation model (nnU-Net), its primary focus is on applying this established deep learning framework to build a standardized eye atlas, rather than developing a novel segmentation method. The use of nnU-Net in the context of the MReye dataset may be novel; however, deep learning–based eye segmentation has been explored previously, including:

- *Tahir, Waqar Ahmad, et al. "Extracting Eye Models from MRI Scans Using U-Net-Based Deep Learning Framework." Journal of Computer and Communications 12.11 (2024): 95–107.*
- *Qureshi, Amad, et al. "Deep-learning-based segmentation of extraocular muscles from magnetic resonance images." Bioengineering 10.6 (2023): 699.*
- *Yang, Jin-Ju, et al. "Fully Automated Segmentation of Human Eyeball Using Three-Dimensional U-Net in T2 Magnetic Resonance Imaging." TVST 12.11 (2023): 22–22.*

Other relevant studies using non–deep learning methods include:

- *Zhang, Haiyang, et al. "TOM500: A Multi-Organ Annotated Orbital MRI Dataset for Thyroid Eye Disease." Scientific Data 12.1 (2025): 60.*
- *Zhang, Haiyang, et al. "Artificial intelligence in thyroid eye disease imaging: A systematic review." Survey of Ophthalmology (2025).*

AR: We thank the reviewer for the suggestions to cover the state-of-the-art more comprehensively, complementing references [20–40]. In the edited version, we have moved the second citation ([29] Qureshi, et al. (2023)), which appeared in the discussion, into the Introduction. We have also included the other four references:

Edit: **Original (p. 2, ll. 55–65):**

~~Accurate clinical analysis requires fully automated, robust segmentation of eye and orbit structures.~~ Early semi-automated methods used parametric shape-based models with spheres and ellipsoids ~~[20, 21, 22]~~, but these lacked flexibility for anatomical variability. Active shape models using machine learning improved this by enabling data-driven deformations ~~[23, 24]~~. More recently, deep learning approaches, primarily 2D and 3D U-Nets, have been used to fully automate segmentation ~~[25, 26, 27], with some hybrid models and clustering techniques also explored [28, 29, 30].~~ Yet, these efforts largely focus on a limited set of ocular structures (e.g., lens, globe, sclera, cornea), with rare inclusion of the optic nerve ~~(only [24, 25])~~ and minimal attention to key orbital components like rectus muscles (RM) or orbital fat, therefore limiting the development of a comprehensive eye-orbit model. ~~Furthermore,~~ most studies used small datasets (typically 24 to 40 annotated subjects) and often relied on multi-contrast MRI, restricting generalizability. Importantly, despite the known influence of image quality

on automated neuroimaging analyses ~~[31, 32, 33, 34, 35]~~, quality control is rarely integrated in MR-Eye pipelines, with only a few exceptions ~~[24, 26, 29]~~.

Revised (p. 2, ll. 55–69):

Manual segmentation has traditionally been the reference standard for delineating ocular and orbital structures and tumors [10, 20], but it is labor-intensive, operator-dependent, and not scalable for large studies. Early semi-automated methods used parametric shape-based models with spheres and ellipsoids [20, 22, 23]. Active shape models using machine learning added flexibility for anatomical variability by enabling data-driven deformations [24, 25]. More recently, deep learning approaches, primarily 2D and 3D U-Nets, have been used to fully automate the segmentation [26, 27, 28, 29, 30, 31], with some hybrid models and clustering techniques [29, 30, 32, 33, 34, 36]. Yet, these efforts largely focus on a limited set of ocular structures (e.g., lens, globe, sclera, cornea), with rare inclusion of the optic nerve [25, 27] and minimal attention to key orbital components like rectus muscles (RM) or orbital fat (i.e., intraconal and extraconal), therefore limiting the development of a comprehensive eye-orbit model. Some studies include the segmentation of tumors, usually retinoblastoma or uveal melanoma [25, 28, 32, 33], while our study focuses on healthy adult eye and orbit structures. Moreover, most studies used small datasets (typically 24 to 49 annotated subjects) and often relied on multi-contrast MRI, restricting generalizability. Importantly, despite the known influence of image quality on automated neuroimaging analyses [35, 37, 38, 39, 40], quality control is rarely integrated in MR-Eye pipelines, with only a few exceptions [25, 27, 33].

Comment #3:

Scientific Contribution and Novelty #2
While the manual segmentation phase could have been more rigorously designed, the inclusion of a senior radiologist for review provides reasonable assurance of accuracy. A similar strategy was used in Zhang et al. (2025), where three junior radiologists independently performed segmentation, which was later unified using an EM-based method and reviewed by an expert.

AR: We thank the reviewer for this observation. As described in the manuscript, manual segmentations were performed independently by a senior and a junior ophthalmologist, with the senior expert reviewing and correcting all annotations. This two-step review process ensured consistency and accuracy comparable to multi-rater aggregation schemes such as the EM-based consensus strategy proposed by Zhang et al. (2025).

Comment #4:

Technical Clarifications and Missing Analyses #1
In the Dice Similarity Coefficient (DSC) equation, the terms A and A_hat should be explicitly defined as the ground truth and predicted areas, respectively.

AR: We thank the reviewer for noticing this oversight and have addressed the issue accordingly:

Edit: **Original (p. 7, ll. 331):**

It is negatively biased by small structures.

$$DSC = \frac{2|A \cap \hat{A}|}{|A| + |\hat{A}|}$$

Revised (p. 10 ll. 416):

It is negatively biased by small structures.

$$DSC = \frac{2|A \cap \hat{A}|}{|A| + |\hat{A}|},$$

where A represents the ground truth and $\hat{A}$ the predicted area.

Comment #5:

Technical Clarifications and Missing Analyses #2
Statistical significance testing is missing in Table 2. While the reported DSC values (typically >80%) suggest good performance, without formal statistical testing, the robustness of the method cannot be confidently assessed. One suggestion is to report p-values for correlations among metrics such as DSC vs. DH, DSC vs. VD, and DH vs. VD. These correlations could indicate whether model performance degrades in anatomically complex or ambiguous ROIs, thus validating sensitivity to segmentation difficulty.
Alternatively, variability across segmentations done by independent radiologists could be measured and correlated with nnU-Net performance [similar to the ICC-based approach used in Zhang et al. (2025)]. This would demonstrate whether the model performance reflects expected levels of variability in the data.

AR: We thank the reviewer for this suggestion. We computed correlations between the metrics (DSC, HD, VD), which are summarized in Supplementary Figure S2. As expected, DSC is negatively correlated with HD and VD, and HD and VD are positively correlated, indicating that better overlap corresponds to lower boundary and volume errors. All correlations are statistically significant ($p < 0.05$).

Linked to comment #11, We now report both the mean ± standard deviation with 95% confidence intervals and the median [interquartile range] with 95% confidence intervals obtained via bootstrap resampling.

Edit: **Original (p. 3, ll. 125–130):**

Revised (p. 3, ll. 129–134):

As expected, lower performance was encountered in more anatomically variable structures(fat, superior RM). Consistent with these metrics, in S2 Figure we observe strong relationships between them across all regions. DSC is negatively correlated with HD and VD, indicating that higher overlap corresponds to better contour and volume agreement, while HD and VD are positively correlated, showing that larger boundary errors tend to be associated with larger volume differences. Weaker correlations are found in optic nerve and rectus muscles, probably

due to their variable shape across subjects. All correlations are statistically significant ($p < 0.05$).

S2 Figure. Pairwise correlations between segmentation metrics across regions. Heatmaps show Pearson correlations between DSC (3D overlap, higher is better), HD (boundary distance, lower is better), and VD (volume difference, closer to 0 is better). Negative DSC–HD and DSC–VD correlations indicate that better overlap corresponds to better contour and volume agreement, while positive HD–VD correlations indicate that larger boundary errors are associated with larger volume differences. All correlations are significant ($p < 0.05$).

AR: Regarding the variability across segmentations, unfortunately, in our dataset we only received one manual segmentation per subject, so it is not possible to compute inter-rater variability or ICC as done in Zhang et al. (2025). We agree that such an analysis would provide important context, and we highlight this as an avenue for future work in the discussion section.

Edit: **Original (p. 4, ll. 199–202):**

> A comparison table of the performances of these previous methods can be found in [28], with DSC ranges for lens of [0.77, 0.91], for globe (referred as vitreous humor) of [0.92, 0.95], and for sclera (in some cases including the vitreous humor) of [0.84, 0.95], with few reports of DSC values of the optic nerve [0.79, 0.82], rectus muscles or fat.

Revised (p. 7, ll. 252–259):

> A comparison table of the performances of these previous methods can be found in [28], with DSC ranges for lens of [0.77, 0.91], for globe (referred as vitreous humor) of [0.92, 0.95], and for sclera (in some cases including the vitreous humor) of [0.84, 0.95], with few reports of DSC values of the optic nerve [0.79, 0.82], rectus muscles or fat. It would also be valuable to assess inter-rater variability by including multiple independent manual annotations, as in [20], where inter-rater agreement was quantified via ICC. However, in our study, only a single manual segmentation per subject was available, which prevented such an analysis. We acknowledge this as a limitation and consider it an important direction for future work to further contextualize the robustness of automated segmentation relative to human variability.

Comment #6:

Technical Clarifications and Missing Analyses #2
Figure 3 appears to evaluate model performance against variability in image quality. While the figure is insightful, it does not directly support the claim of high segmentation performance. The DSC values remain consistently high and appear weakly correlated with image quality scores. This may suggest that even low-quality images (score = 0) are sufficient for the model to perform well. I suggest moving this figure to the supplementary section, as it does not substantiate the main performance claims.

AR: We thank the reviewer for this observation. We agree that Figure 3 primarily illustrates robustness to (included) image quality (rating scores above 1) rather than directly supporting the main performance claims. Following the reviewer's suggestion, we have moved this figure to the Supplementary Information (now S1 Figure) and adjusted the text accordingly.

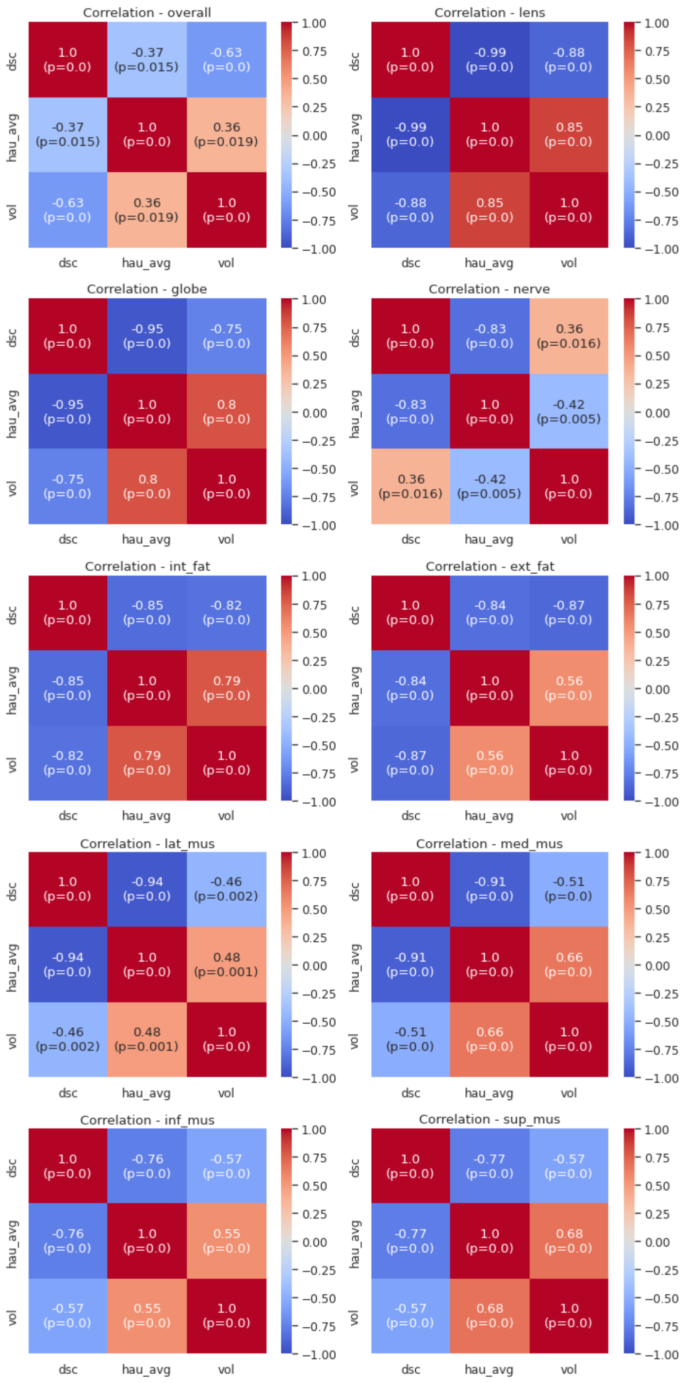

Figure 1: S2 Fig

Edit: **Original (p. 3, ll. 132–134):**

> In addition, to evaluate the robustness of the nnU-Net, we computed correlation between the eye-quality ~~and the nnU-Net's performance of the mentioned 43 images, by means of the DSC. It performed well even in low-quality images, see Fig 3.~~

Revised (p. 4, ll. 155–157):

> In addition, to evaluate the robustness of the nnU-Net, we computed the correlation between eye-quality scores and segmentation performance (DSC) across the 43 test images. The correlations were near zero, suggesting that the model performance was not strongly influenced by image quality (see S1 Figure).

Reviewer #2

Comment #7:

Barranco et al. present an innovative and potentially impactful study, introducing an automated MRI-based ocular segmentation framework and a large-scale atlas of ocular anatomy. The work addresses an important gap in extracting ocular biomarkers from MRI and offers a valuable resource for the research and clinical community. However, several aspects of the manuscript require revision before the findings can be fully justified and reproducible.

AR: We thank the reviewer for the positive feedback on the relevance and potential impact of our work. We also appreciate the constructive suggestions provided and have revised the manuscript accordingly to improve clarity, reproducibility, and justification of our findings.

Comment #8:

Several claims of "to the best of our knowledge" are made throughout the manuscript with only some supporting citations. These statements should be rephrased more cautiously or substantiated with a broader review of recent work.

AR: We thank the reviewer for this important point. We have rephrased the "to the best of our knowledge" statements to be more cautious and have substantially expanded the literature review and added additional recent citations throughout the manuscript.

Edit: **Original (p. 2, ll. 72–74):**

> While the automated extraction of such biomarkers from MR-Eye could benefit clinics and research in terms of time and performance, ~~to the best of our knowledge,~~ no existing tools support automated estimation of AL nor volumetry from 3D MRI.

Revised (p. 2, ll. 76–78):

> While the automated extraction of such biomarkers from MR-Eye could benefit clinics and research in terms of time and performance, no existing tools support automated estimation of AL (apart from [48], in Japanese) nor volumetric values from 3D MRI.

Edit: **Original (p. 2, ll. 88–89):**

> Yet, comparable tools are—~~to the best of our knowledge~~—notably absent in ophthalmic imaging.

Revised (p. 2, ll. 92–93):

> Yet, comparable tools are notably absent in ophthalmic imaging.

Edit: **Original (p. 3, ll. 112–113):**

> ~~Leveraging the extensive scale of our database,~~ we introduce, ~~for the first time to our knowledge,~~ a large-scale atlas of the eye in MRI.

Revised (p. 3, ll. 112–113):

> We introduce a large-scale (N≫100) atlas of the eye in MRI.

Edit: **Original (p. 5, ll. 204–205):**

> ~~To our knowledge,~~ our study reports ~~for the first time~~ the anatomical delineation of structures such as fat and rectus muscles in 3D; RMs segmentation was ~~only~~ presented so far in 2D [72].

Revised (p. 7, ll. 268–269):

> Our study reports the anatomical delineation (e.g. volumetry) of structures such as orbital fat and rectus muscles directly extracted in 3D–RMs segmentation was presented so far only in 2D [28].

Comment #9:

The Introduction is comprehensive but somewhat lengthy. Consider condensing descriptions of segmentation methods and prior studies. Clearly highlighting the specific research gap earlier would improve reader engagement.

AR: We thank the reviewer for this suggestion. The Introduction has been streamlined by condensing the descriptions of prior segmentation methods and studies (see comment #2) and contributions (see comment #10).

Comment #10:

Line 96: The contributions paragraph summarizes the study's novelty but mixes details that might be better placed in the Methods section.

AR: We thank the reviewer for this helpful suggestion. We have revised the contributions paragraph to focus on the high-level novelty of our work, while moving methodological details (e.g., choice of segmentation method, evaluation metrics, and annotated subset size) to the Materials and Methods and Results sections. We believe this improves the clarity and readability of the manuscript.

Edit: **Original (p. 3, ll. 96–109):**

> The contributions of this work are three-fold. First, we present a comprehensive and accurate 3D segmentation framework ~~for healthy adult human eye and orbit structures—including the lens, vitreous humor (VH), optic nerve, rectus muscles (RM), and orbital fat—using T1-weighted MRI data from 1,245 healthy subjects. We employed the state-of-the-art deep learning segmentation method, nnU-Net [59], chosen for its superior performance in medical image segmentation tasks, including MR-Eye. Its performance was evaluated on a manually annotated subset (N=74) using classic segmentation similarity quality metrics such as Dice Similarity Coefficient (DSC), Hausdorff Distance (HD), and Volume Difference (VD).~~ Second, ~~this segmentation pipeline enabled~~ automated large-scale extraction of key ocular biomarkers, namely axial length and structure-specific volumetry, across the full ~~large-scale~~ cohort. Third, ~~we introduce~~ the first unbiased, large-scale T1w MR-Eye atlases—stratified by sex (594 males, 616 females) and combined (1,210 subjects)—with detailed labels of eye and orbital anatomy. These atlases are ~~provided in~~ standard volumetric coordinate spaces (VCS) [51, 52], and are publicly available [60], offering a foundational spatial reference for MR-Eye ~~clinics and research.~~ To support these contributions, we developed a dedicated MR-Eye quality control (QC) protocol, ~~addressing the limitations of existing brain QC tools, which failed to adequately assess ocular image quality.~~

Revised (p. 3, ll. 100–109):

> The contributions of this work are three-fold. First, we present a comprehensive and accurate 3D segmentation framework [59] for healthy adult human eye and orbit structures—including the lens, globe, optic nerve, rectus muscles, and orbital fat—using T1-weighted MRI data from 1,245 healthy subjects. Second, building on this framework, we enable automated large-scale extraction of key ocular biomarkers, namely axial length and structure-specific volumetry, across the full cohort. Third, we provide the first unbiased, large-scale T1w MR-Eye atlases—stratified by sex (594 males, 616 females) and combined (1,210 subjects)—with detailed labels of eye and orbital anatomy. These atlases are made publicly available [60] in standard volumetric coordinate spaces (VCS) [51, 52], offering a foundational spatial reference for MR-Eye research and clinical applications. In support of these contributions, we also introduce a dedicated MR-Eye quality control (QC) protocol tailored to ocular imaging, overcoming the limitations of existing brain QC tools.

Comment #11:

In Results, DSC, HD, and VD values are reported, but the absence of confidence intervals, p-values, or effect sizes limits interpretability. The single reference to [24] provides only partial benchmarking. A table summarizing dataset size, MRI protocol, and segmentation targets across prior studies would clarify performance comparisons.

AR: We thank the reviewer for this helpful suggestion. We have updated Table 1 to provide more comprehensive descriptive statistics for each structure on N = 43 subjects. For each metric (DSC, HD, and VD), we now report both the mean ± standard deviation with 95% confidence intervals and the median [interquartile range] with 95% confidence intervals obtained via bootstrap resampling. No inferential statistical comparisons between metrics were performed, as the purpose of this table is to summarize segmentation performance descriptively.

We have also created a table comparing our study with prior work (extending the one in [27]) that

summarizes reference, model used, dataset size, pulse sequence, and reported performance (DSC). This allows for a clearer overview of related studies. However, as noted in the manuscript, a direct performance comparison remains challenging due to substantial differences in imaging protocols (e.g., multi-contrast MRI vs. single-modality acquisitions such as T2w), segmentation targets (e.g., sclera, VH, lens, or tumor), and methodologies (e.g., ASM, 2D/3D U-Net).

Edit: **Original (p. 16, ll. 750–752):**

Table 1: Table 1. Similarity metrics' median and standard deviation values per structure on $N = 43$ subjects.

Structure	DSC	HD	VD
Average	0.81±0.07	0.35±0.20	0.19±0.14
Lens	0.86±0.08	0.13±0.07	0.20±0.23
Globe	0.94±0.03	0.07±0.05	0.06±0.08
Optic nerve	0.80±0.07	0.41±0.24	-0.01±0.28
Intraconal fat	0.76±0.11	0.40±0.37	0.32±0.27
Extraconal fat	0.73±0.17	0.44±0.82	0.42±0.33
Lateral RM	0.82±0.14	0.25±0.22	0.17±0.26
Medial RM	0.85±0.09	0.19±0.17	0.09±0.20
Inferior RM	0.85±0.08	0.21±0.28	0.07±0.19
Superior RM	0.77±0.10	0.40±0.59	0.17±0.32

Revised (p. 3, ll. 138–142):

Edit: **Original (p. 3, ll. 126–130):**

~~While a direct comparison with previous studies [20, 21, 22, 23, 24, 25, 26, 27, 28, 29, 30] is jeopardized by differences in imaging protocols (e.g., multi-contrast MRI), segmentation targets, and methodologies (e.g., ASM, 2D/3D U-Net), one related study [24] using T1-weighted images and 3D U-Net architecture in healthy adults reported DSC values of 0.88 and 0.92 for the lens and VH, respectively—providing a rough reference point.~~

Revised (p. 4, ll. 143–147):

To facilitate comparison with previous work [20-34], we provide in Table 2 a summary of prior studies, including reference, model used, dataset size, pulse sequence, and reported performance (DSC). While this contextualizes our results within the literature, a direct comparison is limited by differences in imaging protocols (multi-contrast MRI vs. single-modality acquisitions), segmentation targets (e.g., sclera, VH, lens, or tumor), and methodological approaches (e.g., ASM, 2D/3D U-Net).

Edit: **Original ():**

Revised (p. 4, ll. 149–153):

Table 2: Table 1. Descriptive statistics of similarity metrics per structure on $N = 43$ subjects. For each metric, both the mean ± standard deviation (with 95% confidence interval) and the median [interquartile range] (with 95% confidence interval from bootstrap resampling) are reported. No statistical comparisons between metrics were performed.

**Structure**	**DSC**	**HD**	**VD**
Average	0.81±0.07 (mean 0.80 ± 0.07, CI: [0.78–0.82]; median 0.81 [0.75–0.85], CI: [0.76–0.83])	0.35±0.20 (mean 0.37 ± 0.20, CI: [0.31–0.43]; median 0.35 [0.23–0.44], CI: [0.30–0.40])	0.19±0.14 (mean 0.18 ± 0.14, CI: [0.14–0.22]; median 0.19 [0.07–0.27], CI: [0.12–0.24])
Lens	0.86±0.08 (mean 0.84 ± 0.09, CI: [0.81–0.87]; median 0.86 [0.80–0.90], CI: [0.84–0.89])	0.13±0.07 (mean 0.14 ± 0.07, CI: [0.12–0.16]; median 0.13 [0.09–0.18], CI: [0.11–0.15])	0.20±0.23 (mean 0.24 ± 0.23, CI: [0.17–0.31]; median 0.20 [0.07–0.35], CI: [0.15–0.29])
Globe	0.94±0.03 (mean 0.94 ± 0.03, CI: [0.93–0.95]; median 0.94 [0.93–0.95], CI: [0.94–0.95])	0.07±0.05 (mean 0.07 ± 0.05, CI: [0.05–0.09]; median 0.07 [0.05–0.08], CI: [0.05–0.08])	0.06±0.08 (mean 0.06 ± 0.08, CI: [0.04–0.08]; median 0.06 [0.01–0.10], CI: [0.02–0.08])
Optic nerve	0.80±0.07 (mean 0.80 ± 0.07, CI: [0.78–0.82]; median 0.80 [0.76–0.85], CI: [0.77–0.82])	0.41±0.24 (mean 0.41 ± 0.25, CI: [0.34–0.48]; median 0.39 [0.23–0.57], CI: [0.26–0.50])	-0.01±0.28 (mean -0.00 ± 0.28, CI: [-0.08–0.08]; median -0.01 [-0.25–0.24], CI: [-0.16–0.14])
Intraconal fat	0.76±0.11 (mean 0.75 ± 0.11, CI: [0.72–0.78]; median 0.76 [0.68–0.84], CI: [0.70–0.83])	0.40±0.37 (mean 0.46 ± 0.37, CI: [0.35–0.57]; median 0.40 [0.28–0.49], CI: [0.32–0.47])	0.32±0.27 (mean 0.34 ± 0.27, CI: [0.26–0.42]; median 0.32 [0.16–0.49], CI: [0.23–0.44])
Extraconal fat	0.73±0.17 (mean 0.66 ± 0.17, CI: [0.61–0.71]; median 0.73 [0.53–0.79], CI: [0.61–0.77])	0.44±0.82 (mean 0.74 ± 0.82, CI: [0.49–0.99]; median 0.42 [0.32–0.93], CI: [0.35–0.66])	0.42±0.33 (mean 0.47 ± 0.33, CI: [0.37–0.57]; median 0.42 [0.22–0.70], CI: [0.31–0.57])
Lateral RM	0.82±0.14 (mean 0.76 ± 0.14, CI: [0.72–0.80]; median 0.82 [0.69–0.86], CI: [0.75–0.85])	0.25±0.22 (mean 0.32 ± 0.23, CI: [0.25–0.39]; median 0.24 [0.15–0.45], CI: [0.17–0.34])	0.17±0.26 (mean 0.15 ± 0.26, CI: [0.07–0.23]; median 0.17 [-0.01–0.27], CI: [0.08–0.24])
Medial RM	0.85±0.09 (mean 0.83 ± 0.09, CI: [0.80–0.86]; median 0.85 [0.80–0.88], CI: [0.81–0.88])	0.19±0.17 (mean 0.24 ± 0.18, CI: [0.19–0.29]; median 0.19 [0.14–0.28], CI: [0.16–0.25])	0.09±0.20 (mean 0.11 ± 0.20, CI: [0.05–0.17]; median 0.09 [-0.02–0.22], CI: [0.02–0.15])
Inferior RM	0.85±0.08 (mean 0.84 ± 0.08, CI: [0.82–0.86]; median 0.85 [0.81–0.90], CI: [0.82–0.87])	0.21±0.28 (mean 0.27 ± 0.28, CI: [0.18–0.36]; median 0.19 [0.14–0.31], CI: [0.15–0.23])	0.07±0.19 (mean 0.09 ± 0.19, CI: [0.03–0.15]; median 0.07 [-0.01–0.20], CI: [0.01–0.13])
Superior RM	0.77±0.10 (mean 0.75 ± 0.10, CI: [0.72–0.78]; median 0.77 [0.71–0.83], CI: [0.73–0.80])	0.40±0.59 (mean 0.65 ± 0.59, CI: [0.47–0.83]; median 0.40 [0.26–0.85], CI: [0.32–0.65])	0.17±0.32 (mean 0.15 ± 0.32, CI: [0.05–0.25]; median 0.17 [-0.02–0.31], CI: [0.10–0.25])

Table 3: Table 2. Comparison of our study with prior work on eye and orbit segmentation from MRI. The table summarizes reference, model used, dataset size, pulse sequence, and reported performance (DSC). Note that differences in imaging protocols, segmentation targets, and methodologies limit the possibility of direct performance comparisons.

Ref.	Model	Dataset	Pulse	Scl.	VH	Lens	Tum.	ON	LR	MR	IR	SR	SO	IF	EF
Current	nnU-Net (3D)	3D-MR, 74 healthy	T1	–	0.94	0.86	–	0.80	0.82	0.85	0.85	0.77	–	0.76	0.73
Yang	2D/3D U-Net	2D-MR, 119 mixed	T2	>0.95*	–	–	–	–	–	–	–	–	–	–	–
Qureshi (2023)	U-Net, DeepLabV3+, ConResNet	2D-MR, 38 subj.	T1, T2	–	–	–	–	–	0.82	0.92	0.90	0.80	0.79	–	–
Strijbis (2021)	MV-CNN	2D-MR, 46 eyes (RB, healthy)	T1c, T1, T2, FIESTA	0.84	0.93	0.91	0.84	–	–	–	–	–	–	–	–
Nguyen (2019)	2D U-Net + ASM/CRF	3D-MR, 24 UM	T1, T2	–	–	–	0.84	–	–	–	–	–	–	–	–
Nguyen (2018a)	3D ASM	3D-MR, 7 UM, 30 healthy	T1	0.95*	0.92	0.88	–	0.82	–	–	–	–	–	–	–
Nguyen (2018b)	3D U-Net	3D-MR, 72 eyes (RB, healthy)	T1, T2	0.95*	–	0.87	0.59	0.79	–	–	–	–	–	–	–
Ciller (2017)	3D ASM + CNN	3D-MR, 16 RB eyes	3D T1c, T1, T2	0.95*	0.95	0.86	0.62	–	–	–	–	–	–	–	–
Ciller (2015)	3D ASM	3D-MR, 24 healthy	3D T1c	0.95*	0.95	0.85	–	–	–	–	–	–	–	–	–

Comment #12:

Line 133: The claim that the model "performed well even in low-quality images" is not fully supported. Figure 3 shows near-zero correlations between quality ratings and DSC, but no direct statistical comparison (mean ± SD, p-values) across quality tiers.

AR: We thank the reviewer for this comment. We agree that the original phrasing overstates the results. We have addressed this question in Comment #6.

Comment #13:

Line 151: The inability to compute AL in 107/1157 cases is acknowledged, but there is no analysis of whether these missing cases differ (e.g., sex, age, image quality) from the analyzed cohort. This could bias volumetric and atlas results.

AR: We thank the reviewer for this insightful comment. As several reviewers (comments 13, 15, 22, 31, and 32) pointed out limitations in our previous axial length (AL) estimation approach, we have now implemented a new and more robust 3D method to compute AL. In the revised approach, AL is computed in 3D rather than from a single 2D slice, leveraging the available 3D segmentations of ocular structures (explained below).

This improved method allowed us to compute AL in nearly all subjects, reducing the number of missing cases from 107 (9%) to only 28 (2%). The remaining 28 cases could not be processed due to: (1) 20 missing lens segmentations, typically caused by poorly depicted lenses in the T1-weighted images; (2) 7 instances where the left eye was segmented instead of the right; and (3) 1 case where the lens centroid did not fall within any lens voxel, due to disconnected components in the segmentation (no post-processing applied after inference).

To assess whether the 28 subjects with missing AL values could bias the results, we examined their demographics and image quality. This subset included 19 males and 8 females, with a mean age of

63.5 ± 11.8 years and mean BMI of 30.5 ± 4.4 kg/m^2. Most of these cases had lower image quality, frequently showing left–right phase-encoding artifacts in the orbital region. Despite the low quality observed, these characteristics are consistent with those of the analyzed cohort, suggesting that the small number of missing cases is unlikely to introduce systematic bias in the volumetric or atlas analyses.

This refined 3D approach thus substantially improves AL estimation robustness and mitigates potential bias in the volumetric and atlas analyses. Although the resulting mean values have decreased slightly for both males and females, the standard deviations have also decreased, which is a positive sign. Furthermore, this method allows us to include 79 additional subjects compared to the previous analysis (1129 vs. 1050), where AL couldn't be computed due to the methodological constraints of having segmented structures (lens, globe, and optic nerve) on the same 2D axial plane.

Edit: **Original (p. 7, ll. 349–359):**

> We developed an algorithm to automatically extract the AL ~~(as defined in [36] and illustrated in Fig 4). The method inputs both the automated segmented labels and T1w images. First, we computed the centroid of the lens and an orthogonal line to the globe in the 2D axial slice, measuring the distance between the extreme intersection points of this line with the lens and the globe. Additionally, we calculated two extra distances: from the globe to the intraconal fat boundary, and from the lens boundary to the cornea. To address the lack of cornea segmentation, we determined the second distance by detecting low-intensity voxels (as the cornea appears black in T1w images) and identifying a significant intensity increase with the help of Sobel filter. This process was applied to multiple slices from the same subject where required segmented structures to compute the measurement were present (lens, globe, optic nerve and intraconal fat). If that was not the case, the AL would be saved as 0 mm. The final AL for a subject was determined by selecting the slice with the highest lens-to-optic nerve ratio (and whose AL was non-zero), indicating the best alignment of these structures to compute the measurement.~~

Revised (p. 10, ll. 434–443):

> We developed an algorithm to automatically extract the AL, defined in [40] as the distance from the posterior surface of the cornea to the posterior pole of the ocular bulb, at the boundary with orbital fat (the image had to include the corneal apex as well as the optic nerve head), and illustrated in Fig. 3. The method inputs both the automated segmented labels and T1w images. First, we determine the line connecting the centroids of the lens and the globe, and identify its extreme intersection points with these segmented structures. To estimate the anterior corneal boundary—since manual cornea segmentation is unavailable—we analyze the intensity gradient along the same line. The first peak typically corresponds to the eyelid, and the second to the cornea (the third to the lens) in images with eyes closed. When the cornea could not be detected (198/1157 cases, 17%), usually due to open eyes, the anterior corneal distance was defined as the median value observed in subjects with eyes closed (83%). The total AL is then defined as the distance from the cornea to the posterior pole of the globe.
>
> Fig 9. Example of AL extraction in T1w MRI. A) The intensity and gradient profiles of the line crossing the image. The intersection points of the line with the different structures are shown in the plot with different colors. The cornea is detected as the second brightness peak in the gradient profile. B) Different representations of the automatic extraction using the segmented

structures and the T1w image; on the right, gradient image visual aid. The cornea, in the gradient image, can be seen as the bright area between the eyelid and the lens. The selected axial slice corresponds to the centroid of the globe, and the line and other intersection points are projected onto this slice.

Edit: **Original (p. 3, ll. 142–149):**

Our findings show that automated measurements of AL from MRI are in line with the reference manual measures ~~[36]~~. ~~Fig 4~~ shows the correlation plot of the extracted values, grouped by sex as in ~~[22]~~, on the manually annotated cohort of 43 testing subjects~~, resulting in 41 after removing zero values~~. The average AL from nnU-Net segmentation was closer to the ~~ground truth values~~ which were extracted using the same AL extraction method on the manual segmentations. For the large-scale cohort of 1157 subjects, the mean values and standard deviations~~, after removing zero values,~~ show close values to the manual reference as well:

- ~~NnU-Net: 23.8±1.7 mm (524 M) and 22.9±1.6 mm (526 F)~~
- Previous studies ~~[36]~~: 23.4±0.8 mm (1059 M) and 22.8±0.9 mm (867 F)

~~Fig 4. Axial length (posterior surface of the cornea to the posterior pole of the ocular bulb, at the boundary with orbital fat grouped by sex. (A) Example of AL extraction in MRI: on the left, manual extraction as in [36]; in the middle, automatic extraction using the segmented structures and the T1w image; and on the right, Sobel-filtered image visual aid. (B) Correlation curve and coefficient on N=41 (non-zero values) with respect to the subject-wise manual annotations, which serve as ground truth. (C) Boxplots of the obtained AL per sex computed on the large-scale dataset (N=1157).~~

~~However, in 107/1157 cases, the AL could not be computed due to methodological constraints (e.g., absence of clearly depicted lens in 79 subjects, see Materials and Methods section).~~

Revised (p. 5, ll. 164–170):

Our findings show that automated measurements of AL from MRI are in line with the reference manual measures [40]. Fig 3 shows the correlation plot of the extracted values, grouped by sex as in [22, 40], on the manually annotated cohort of 43 testing subjects. The average AL from nnU-Net segmentation was closer to the GT values which were extracted using the same AL extraction method on the manual segmentations. For the large-scale cohort of 1157 subjects, the mean values and standard deviations show close values to the manual reference as well:

- nnU-Net: 22.71±1.49 mm (549 M) and 21.79±1.51 mm (580 F)
- Previous studies [40]: 23.4±0.8 mm (1059 M) and 22.8±0.9 mm (867 F)

Fig 3. Axial length grouped by sex. A) Correlation curve and coefficient on N=43 with respect to the subject-wise manual annotations, which serve as ground truth. The plot shows moderate to high correlation between both sets. B) Boxplots of the obtained AL grouped by sex computed on N=1129. The plots show values close to the references.

However, in 28/1157 (2%) cases, the AL could not be computed due to: 1) 20 missing lens segmentations, typically caused by poorly depicted lenses in the T1-weighted images; 2)

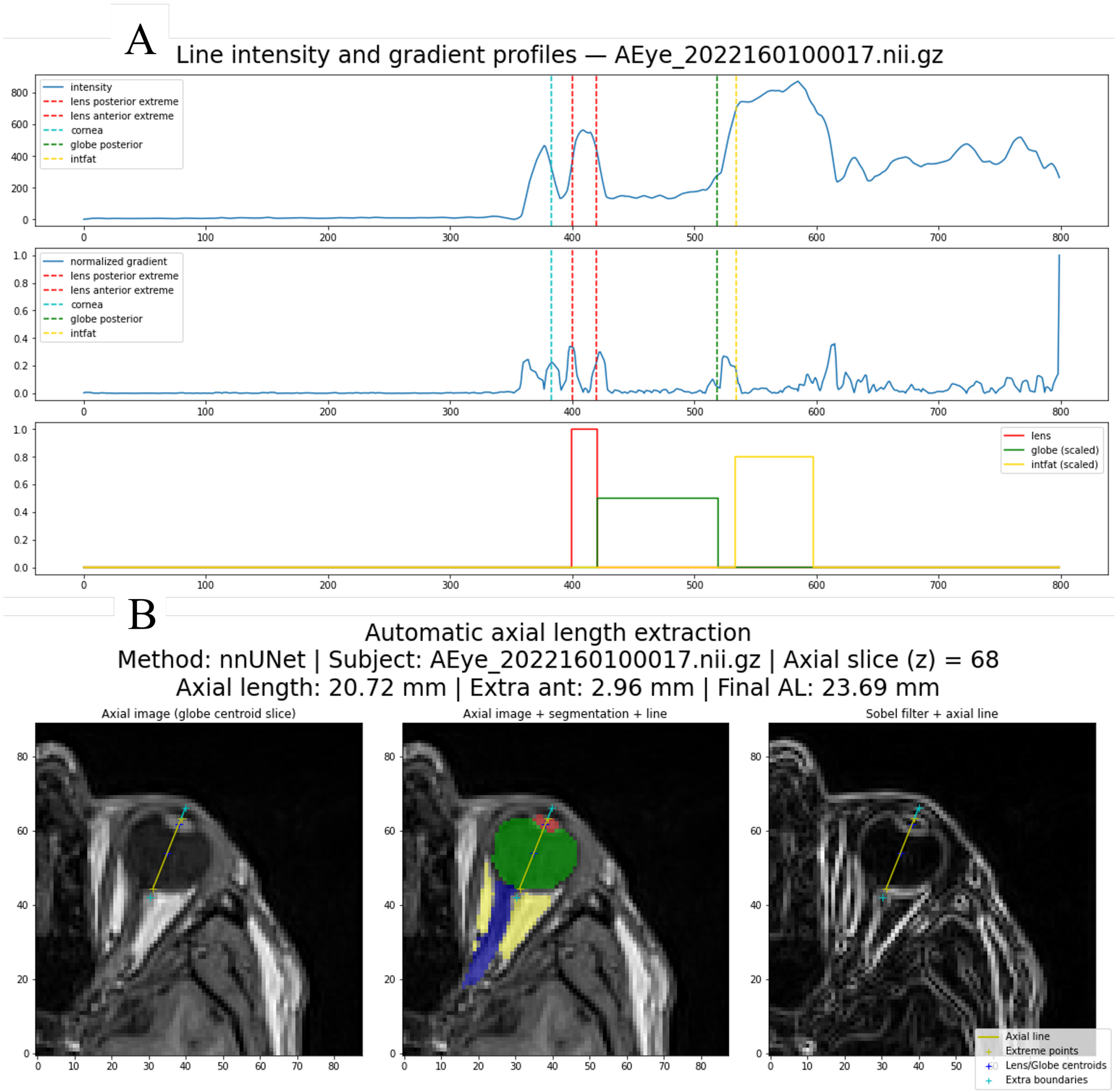

Figure 2: Fig 9

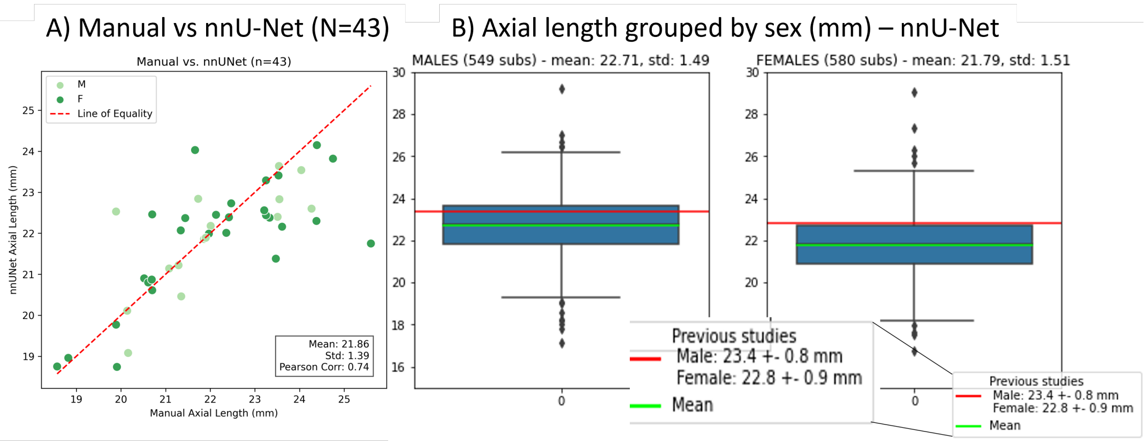

Figure 3: Fig 3

7 instances where the left eye was segmented instead of the right; and 3) 1 case where the lens centroid did not fall within any lens voxel, due to disconnected components in the segmentation (no post-processing applied after inference). To evaluate potential bias introduced by the remaining 28 cases with missing AL measurements, we analyzed their demographic and image-quality characteristics. This subset included 19 males and 8 females, with a mean age of 63.5 ± 11.8 years and a mean BMI of 30.5 ± 4.4 kg/m^2. Image quality was generally lower than average, often showing left–right phase-encoding artifacts near the eyes. Overall, these characteristics are comparable to those of the complete cohort, indicating that the small number of excluded cases is unlikely to affect the overall volumetric or atlas results.

Comment #14:

Line 206: The claim that the method could save "10–20 minutes per eye" for clinicians is compelling but should be supported by citing the source of that time estimate.

AR: We thank the reviewer for pointing this out. The estimate of "10–20 minutes per eye" has now been supported by explicitly attributing it to the experience of our senior radiologist (SL). The text has been revised accordingly.

Edit: **Original (p. 5, ll. 206–208):**

With its high accuracy, it could be seamlessly integrated into MRI console analysis, potentially saving clinicians up to 10 to 20 minutes they currently spend on manual segmentation.

Revised (p. 7, ll. 269–271):

With its high accuracy, it could be seamlessly integrated into MRI console analysis, potentially saving clinicians up to 10 to 20 minutes (according to SL, senior radiologist) they currently spend on manual segmentation.

Comment #15:

Line 220: The zero AL values due to absent lens segmentation show a failure mode of the method. Quantify the frequency of such cases in the test set and discuss mitigation strategies.

AR: We thank the reviewer for raising this point. As noted in comment #13, using the revised 3D AL computation, only 28/1157 cases (2%) could not be measured due to missing lens segmentations (20), segmentation on the left eye (7), or disconnected lens components (1) due to the absence of any post-processing after the inference. Mitigation strategies could include the use of symmetric segmentation and the use of connected components as post-processing techniques, and a more strict QC to discard images without clearly depicted lens in the T1w image.

Edit: **Original (p. 5, ll. 220–228):**

> To further validate our pipeline, we introduce an automated method to measure AL from segmented MR-Eye volumes. ~~Our automated results closely match the reference manual measures of AL [36, 37, 38], performed by expert ophthalmologists, on a testing set of 41 subjects with GT and a large-scale database of 1,157 subjects.~~ This reinforces the reliability of our automated approaches for both eye structure segmentation and AL extraction. ~~For most of the cases where the computed AL was zero, the lens (a critical tissue to extract this biomarker in our algorithm) was not present, or not present clearly, on the original T1w image, hence the failure of the nn-UNet to segment a non-existing structure.~~ This method could be valuable in certain clinical cases, such as in patients with rhegmatogenous retinal detachments with macular detachment or vitreous and submacular hemorrhage, where preoperative axial length is often underestimated ~~[74]~~.

Revised (p. 7, ll. 283–294):

> To further validate our pipeline, we introduce an automated method to measure AL from segmented MR-Eye volumes. Our automated results, both the testing set of 43 subjects with GT and the large-scale database of 1,157 subjects, closely match the reference measures of AL [40, 41, 42], performed manually by expert ophthalmologists. This reinforces the reliability of our automated approaches for both eye structure segmentation and AL extraction. In 2% of cases (28/1157), the computed AL was zero. Among these, 20 cases were due to the absence of the lens in the T1w image, which prevented nnU-Net from segmenting this structure; in 7 cases, the model segmented the structures on the left eye; and in 1 case, the centroid fell in a voxel where there was no lens segmentation due to the absence of any post-processing after the inference. Mitigation strategies could include the use of symmetric segmentation and the use of connected components as post-processing techniques, and a more strict QC to discard images without clearly depicted lens in the T1w image. Nevertheless, this automated method could be valuable in specific clinical scenarios, such as patients with rhegmatogenous retinal detachments with macular detachment or vitreous and submacular hemorrhage, where preoperative axial length is often underestimated [78].

Comment #16:

Line 232: The observation of sex differences in eye structure volumes is interesting, but the manuscript should comment on possible confounders (head size, body height) beyond the mention

> *of fat volume in line 260.*

AR: We thank the reviewer for raising this important point. We have revised the manuscript to acknowledge the potential influence of additional confounders such as head size and body height on the observed sex differences in eye structure volumes.

Edit: **Original (p. 5, ll. 239–240):**

> This finding suggests that variations in eye structure volumes may not be associated with BMI.

Revised (p. 7, ll. 305–309):

> This finding suggests that variations in eye structure volumes may not be associated with BMI. Nevertheless, other potential confounding factors, such as head size or body height, could partly explain the observed sex differences. While these parameters were not explicitly accounted for in our analysis, future work incorporating them may help disentangle whether the differences observed are primarily attributable to sex-specific anatomy or to broader anthropometric variability.

Comment #17:

> *Line 249: The claim that ethnic homogeneity does not compromise generalizability is too strong, especially since it is supported by a single reference. Consider softening the statement to reflect possible limitations, or add multiple sources covering diverse populations.*

AR: We thank the reviewer for this valuable comment. We have revised the text to soften the claim regarding ethnic homogeneity and to acknowledge the potential limitation in generalizability.

Edit: **Original (p. 5, ll. 249–251):**

> ~~Although~~ the participants of this cohort predominantly share a similar ethnic background, ~~this does not compromise the generalizability of the findings to other populations, as key ocular anatomical features have been shown to be largely comparable across ethnicities, with only minor differences reported [79].~~

Revised (p. 8, ll. 318–321):

> The participants of this cohort predominantly share a similar ethnic background, which may limit the direct generalizability of the findings to other populations. Nevertheless, previous studies have reported that key ocular anatomical features are largely comparable across ethnicities, with only minor differences observed [83].

Comment #18:

> *The validation cohort for segmentation and axial length is small relative to the overall dataset. While the results are strong, the authors should acknowledge this limitation in representativeness.*

AR: We appreciate the reviewer's observation regarding the size of the validation cohort. We agree that

the size of the manually annotated validation set is modest relative to the overall cohort, and we have now explicitly acknowledged this as a limitation in the manuscript, while emphasizing that:

- The validation against manual references (N=43) shows strong accuracy.
- The large-scale application (N=1,157) produces consistent results with ophthalmological reference standards, addressing representativeness concerns.

Edit: **Original ():**

Revised (p. 7, ll. 261–265):

In addition, while the validation cohort of 43 subjects is modest compared to the overall dataset, the automated segmentations applied to the remaining 1,157 subjects produced axial length distributions consistent with state-of-the-art manual measurements reported in the ophthalmological literature, supporting the generalizability of our findings beyond the directly validated subset.

Comment #19:

In the Methods, the description of study components is clear but reads as a list rather than a rationale. Justify why each step is necessary in the context of MR-Eye and how they build on each other.

AR: We thank the reviewer for the suggestion. We have clarified the rationale of each step, emphasizing how segmentation enables biomarker extraction, quality control ensures robustness, and together they form an integrated MR-Eye pipeline.

Edit: **Original (p. 4, ll. 276–278):**

~~We evaluated quantitatively a deep learning-based automated segmentation method on a cohort of manually segmented subjects using similarity metrics (surface overlap, volume error and distance-based error). Then, we performed a large-scale analysis on relevant ophthalmic biomarkers, namely volumetry of eye structures and AL measurements, for which we developed a method for its automated extraction. This allowed further analysis of possible correlation between volumes and BMI grouped by sex (males and females). We introduced eye-quality control checks that are described later in this section. Thus, the major components of our study were large-scale cohort, automated segmentation methods, automated biometry extraction, quality control analysis, and statistical analysis.~~

Revised (p. 8, ll. 342–347):

To rigorously assess MR-Eye, we first validated a deep learning–based automated segmentation method on manually segmented subjects using similarity metrics (surface overlap, volume error, and distance-based error). Building on this validated framework, we extracted key ophthalmic biomarkers—volumetry of eye structures and axial length—across the large-scale cohort to enable reproducible, clinically relevant measurements, including correlations with BMI stratified by sex. Dedicated eye-quality control checks, described later, ensured

robustness and mitigated imaging artifacts. Together, these components form an integrated pipeline, with each step supporting reliable and generalizable MR-Eye analysis.

Comment #20:

Line 285: The dataset origin is documented, but the statement "The exact criteria or process for this selection were not fully documented" is a limitation and should be explicitly acknowledged in the Discussion section.

AR: We thank the reviewer for this observation. After discussion with the coauthors, SL clarified the dataset selection process. The initial dataset included 3,030 subjects, of which 549 were excluded for insufficient image quality and 555 for missing covariates (cases where axial length but no exophthalmometry could be extracted). Following an additional quality control performed by the SHIP team in 2023, 681 further subjects were removed, resulting in the final dataset of 1,245 subjects provided to us for analysis. We have now clarified this in the revised manuscript.

Edit: **Original (p. 6, ll. 281–292):**

The cohort was originally acquired as part of the Study of Health in Pomerania (SHIP) [36] and reused for the present study. A total of 3030 healthy adult subjects from Northeast Germany underwent whole-body MRI on a 1.5T Magnetom Avanto scanner (Siemens Medical Solutions, Erlangen, Germany) without contrast agent. ~~This dataset includes some cases of lower image quality and missing covariates (e.g., only one evaluable eye or axial length measurements without corresponding exophthalmometry). For the purposes of this study, we received a subset of 1245 subjects, randomly selected from the full cohort. The exact criteria or process for this selection were not fully documented.~~ Participants ranged in age from 28 to 89 years (mean ± SD: 56 ± 13 years). T1-weighted (T1w) images of the head were acquired using a 12-channel head coil, with 176 slices per volume, 1 mm slice thickness, 256 mm field of view, 1 mm^3 voxel size, TR = 1900 ms, TI = 1100 ms, and TE = 3.37 ms. During MRI acquisition, subjects rested their eyes naturally without specific instructions regarding gaze or eyelid position. All participants provided written informed consent. The study was approved by the Medical Ethics Committee of the University of Greifswald and conducted in accordance with the Declaration of Helsinki. All participant data were accessed from an anonymized database.

Revised (p. 8, ll. 350–366):

The cohort was originally acquired as part of the Study of Health in Pomerania (SHIP) [40] and reused for the present study. A total of 3030 healthy adult subjects from Northeast Germany underwent whole-body MRI on a 1.5T Magnetom Avanto scanner (Siemens Medical Solutions, Erlangen, Germany) without contrast agent. For all MRI measurements, the image that bisected the eyeball in the axial plane was chosen. The image had to include the corneal apex as well as the optic nerve head. Consequently, false measurements due to Bell's phenomenon could be avoided. Study participants were excluded from imaging analysis if there was no plane available that included both structures. Further exclusion criterion was a lateral deviation of the subject's viewing direction. Images of inadequate quality due to motion artefacts or other technical factors were excluded as well. Due to these exclusion criteria, which were summarized as "low image quality", 549 subjects had to be precluded. In some cases, only one eye was evaluable or only axial length measurement but

no exophthalmometry was possible. For this reason, another 555 participants were excluded. After another QC by SHIP in 2023, trimming 681 subjects due to insufficient quality, we received a subset of 1245 subjects out of the initial 3030. Participants ranged in age from 28 to 89 years (mean ± SD: 56 ± 13 years). T1-weighted (T1w) images of the head were acquired using a 12-channel head coil, with 176 slices per volume, 1 mm slice thickness, 256 mm field of view, 1 mm^3 voxel size, TR = 1900 ms, TI = 1100 ms, and TE = 3.37 ms. During MRI acquisition, subjects rested their eyes naturally without specific instructions regarding gaze or eyelid position. All participants provided written informed consent. The study was approved by the Medical Ethics Committee of the University of Greifswald and conducted in accordance with the Declaration of Helsinki. All participant data were accessed from an anonymized database.

Comment #21:

Line 315: The choice of splitting into 31 training / 43 testing is unusual for deep learning and might risk undertraining, given the small annotated set. Justification for this split ratio is needed. Also, the manuscript states "five-fold cross-validation" but also a fixed train/test split—potentially inconsistent. Clarify whether the five folds were within the training set only.

AR: We thank the reviewer for raising this point. The train/test split was determined by the availability of annotated data at different stages of the project: initially, 35 subjects were available, of which 31 were used for training/validation and 4 for testing. Afterward, we received 39 additional manually segmented subjects, and the original 4 test cases were incorporated into this larger testing cohort (a total of 43).

To clarify, the reported five-fold cross-validation was performed within the training set only. We have revised the manuscript to make this explicit and to better justify the choice of the split.

Edit: **Original (p. 7, ll. 315):**

We split the manual annotated dataset into 31 for training and 43 for testing.

Revised (p. 9, ll. 396–398):

We split the manual annotated dataset into 31 for training and 43 for testing. The split reflected data availability. Initially, 35 subjects were available, 31 were used for training / validation, and 4 for testing. After receiving 39 additional segmentations, the 4 initial test cases were added to this larger cohort to increase the test set (43 in total).

Edit: **Original (p. 7, ll. 318):**

... five folds cross validation; ...

Revised (p. 9, ll. 401):

... five-fold cross validation for the training; ...

Comment #22:

Line 348: There is a potential circularity risk. AL is computed using segmented labels, and segmentation accuracy could vary with AL itself. Was this evaluated?

AR: We thank the reviewer for this point. While it is true that AL is computed using segmented labels, segmentation accuracy, which was evaluated with similarity metrics on the N=43 test set, cannot vary with AL, so we do not see any potential circularity risk.

Comment #23:

Most figures need larger axis title/font sizes (Figs. 4 and 6 especially) and higher quality. Figure 10.tif contains the entire computer window; it should be cropped.

AR: We thank the reviewer for this comment. We have increased the font sizes in Figs. 2–6, saved the figures at higher quality, and cropped Figure 10 as suggested.

Reviewer #3

Comment #24:

The paper evaluated a well-performing medical image segmentation model, nnU-net on the automatic segmentation of the structures of eye balls, utilizing a large dataset of over 1000 MRI images containing eyes. The work is generally technically sound. However, I feel that there are several aspects that need clarification, and I also worry how much technical contributions have been made by the authors themselves.

AR: We thank the reviewer for the positive assessment of the technical soundness of our work. We will address clarification and technical contributions in the following comments.

Comment #25:

If my understanding is correct, the paper only showed results for the right eye and contributed an atlas for the right eye. This may be due to the limited annotation to the right eyes only. For a paper proposing a new method, it seems fine to just evaluate on one example area. However, since the author did not contribute a new algorithm and the usable tool for users is mainly the atlas (and the web application still under development), it seems hardly useful if users can only segment one eye but not both. If there is no chance to acquire annotation for the left eye, can the trained model generalize to the left eye in any way?

AR: We thank the reviewer for this important observation. It is correct that only right eyes were manually annotated in our dataset, and therefore the current atlas and reported results are limited to the right eye. However, the trained model can in principle generalize to the left eye by flipping the images (left–right axis inversion). We agree that explicitly evaluating this capability would be highly valuable, and we have added a sentence in the Discussion highlighting this as an important direction for future work.

Edit: **Original ():**

Revised (p. 4, ll. 258–260):

> Another limitation is that only right eyes were manually annotated in our dataset, and therefore the current atlas and reported results are restricted to the right eye. However, the trained model can in principle generalize to the left eye through inversion of the left-right axis, and evaluating this capability constitutes an important direction for future work.

Comment #26:

Is the pipeline fully automatic? For example, do the users need to first manually crop the eye region and then send to the pipeline to segment? Does the input image have to have the exactly same size as those being used to train the model, or can the pipeline accommodate variation in image size (and also image resolution)? If not, then this tool is quite limited and needs improvement.

AR: We thank the reviewer for this comment. The proposed pipeline is fully automatic and does not require users to manually crop the images to the eye region prior to segmentation. In addition, the nnU-Net framework can accommodate input images of varying sizes and resolutions, so the inputs do not need to exactly match the training dimensions. This has been incorporated to the paper accordingly.

Edit: **Original (p. 4, ll. 184–186):**

> In this rapidly growing field, it is crucial to enable clinicians to extract measurements from MR-Eye and benchmark new metrics, providing them with tools not available before. To address this need, we propose a comprehensive automated pipeline.

Revised (p. 6, ll. 233–238):

> In this rapidly growing field, it is crucial to enable clinicians to extract measurements from MR-Eye and benchmark new metrics, providing them with tools not available before. To address this need, we propose a comprehensive automated pipeline. The pipeline is fully automatic and does not require manual cropping of the eye region. Moreover, thanks to the nnU-Net framework, the model can handle input images of varying sizes and resolutions, so the inputs do not need to match the training dimensions exactly. This flexibility allows the pipeline to be applied directly to diverse MRI datasets without additional user intervention.

Comment #27:

The rationale of some of the exclusion criteria seems a bit hard to understand. First, I was not sure if the QA/QC is for the purpose of building the atlas, or for the purpose of evaluating the pipeline of segmentation. If it is for building atlas, then some of them may be fine, but if it is for evaluating pipeline, some of them may have helped to make the task easier for the pipeline but may hide any limitation of the tool. For example, while strong motion and artifacts may make segmentation fundamentally challenging (partially true as radiologists sometimes can still read images with artifacts), noise and some degree of blur may be common in practice. A good

> *pipeline should be able to tolerate noise and blur to some degree. When proposing a new tool, it would be very informative to evaluate the performance at different degrees of noise and blur (either by quantifying noise and blur level if possible, or artificially adding noise and blur to see the robustness of the model). This way, users know what to expect based on their image quality.*

AR: We thank the reviewer for this thoughtful comment. To clarify, the QA/QC process was applied only to the large-scale test set in order to exclude images of insufficient quality for reliable evaluation. No images were removed from the training set, and all available cases were included in the atlas construction so as to preserve variability and robustness. As shown in Supplementary Fig. S1 (previously Fig. 3), the correlation between subjective image quality and segmentation performance (DSC) was near zero, suggesting that the model was not strongly influenced by the quality of the included images. While the remaining subjects after QC may still contain some degree of noise and blur, we acknowledge the reviewer's point that a systematic evaluation across controlled levels of noise and blur would further strengthen the robustness assessment.

> **Comment #28:**
>
> *Fig 3 used subjective rating to assess image quality (which does not distinguish different types of image degradation) to show that the model's performance is robust against subjective rating of image quality. This seems great. But if so, why do we still need to exclude very poor images?*

AR: We thank the reviewer for this comment. The plot was intended to illustrate the robustness of the model in different ranges with sufficient image quality. The different dots of the plot represent the images manually segmented, which therefore had already sufficient quality. We excluded images with insufficient quality because they do not allow reliable manual reference segmentations and including them would undermine the evaluation. However, even without reliable ground-truth annotations, the model could produce segmentations, but users should ensure that input images are of sufficient quality to ensure meaningful results.

> **Comment #29:**
>
> *The one critical criterion that I don't understand is the similarity between nnUnet and baseline. The cited paper for the baseline was a demonstration of 7T MRI on imaging some of the structures including optical nerve. It is unclear how that "baseline" is compared here. That paper only included very few subjects (likely not overlapping here) and the magnetic strength is way different from here. I searched for "segment" in the cited paper but it only appeared in the section of OCT, not MRI, even without details of the segmentation tool. So it is not clear how that paper can be compared here. Even if the two papers can be somehow compared, why would outlier need to be excluded if the purpose is to evaluate the method? If a previous method is treated as a gold standard and we exclude outlier values from the current method, it seems to discard any failure cases, which hides the deficit of the current method.*

AR: We thank the reviewer for pointing this out. We apologize for the incorrect reference in the original manuscript. The correct baseline reference is: [77] (former [71]) Barranco J, et al. A-eye: Automated 3D Segmentation of Healthy Human Eye and Orbit Structures and Axial Length Extraction. bioRxiv [Preprint]. 2025; Available from: https://www.biorxiv.org/content/10.1101/2025.05.05.652187v1; doi: 10.1101/2025.05.05.652187. We have updated the manuscript accordingly. The referred baseline is the atlas-based segmentation method, for which we wrote a conference paper with

proceedings about its comparison with nnU-Net.

Edit: **Original (p. 8, ll. 423–424):**

> we computed the already mentioned similarity metrics but this time between the results of the nnU-Net and the baseline [~~70~~] methods...

Revised (p. 11, ll. 523–524):

> we computed the already mentioned similarity metrics but this time between the results of the nnU-Net and the baseline (atlas-based) [77] methods...

Comment #30:

The QC for biomarker is also confusing. It was said that 45 were outliers from the atlas-based method and 150 were nnU-net method. There is no citation for the atlas-based method. In the discussion, it was mentioned "We reported results in the same cohort using an atlas-based segmentation method in our preliminary work [71]". citation 71 has a very similar title giving the same abbreviation of the method (A-eye), and seems to be an earlier version of the current paper, but not a paper proposing an atlas-based paper. This leaves reader clueless of what is being compared against, and why the atlas-based method is mentioned here. The fact that the new approach introduces more outlier than a previous method also suggests it may perform worse.

AR: We thank the reviewer for raising this point. Citation [71] refers to our previous work in which we compared atlas-based segmentation and nnU-Net methods. For the QA/QC process, outliers from both methods were used to discard images with insufficient quality. The nnU-Net method produced more missing values, particularly for axial length (AL), because when the lens is not visible in the T1-weighted image, the model cannot segment it, resulting in an AL value of zero. In contrast, the atlas-based method always includes a lens, even if it is not visible in the original image, since it relies on image registration where the reference atlas contains a lens that is transposed to the subject. We have clarified this in the revised manuscript to better explain the rationale behind using outliers from both methods.

Edit: **Original (p. 4, ll. 195–196):**

> We reported results in the same cohort using an ~~atlas-based~~ segmentation method in our preliminary work [~~71~~].

Revised (p. 6, ll. 247–248):

> We reported results in the same cohort comparing with a baseline (atlas-based) segmentation method in our preliminary work [77], with statistical analysis.

Edit: **Original (p. 9, ll. 432–433):**

> After manual revision, only 2 subjects ~~in total~~ were excluded. In total, in this third step, we removed 23 subjects.

Revised (p. 11, ll. 532–538):

After manual revision, only 2 subjects were excluded. In total, in this third step, we removed 23 subjects. The nnU-Net method produced more outliers, particularly for AL, because when the lens is not visible in the T1w image, the model cannot segment it, resulting in an AL value of zero. In contrast, the atlas-based method always includes a lens, even if it is not visible in the original image, since it relies on image registration where the reference atlas contains a lens that is transposed to the subject. For volumetry, it follows the same reasoning, the atlas-based method would always transpose the structures, unlike the DL method, where sometimes could fail to even segment a single voxel of a specific structure (i.e. the lens).

Comment #31:

I briefly looked into the supplementary materials. The outputs of AL for 150 subjects by the nnUnet approach was zero, matching the number reported above. Any insight of why they are zero? Also, it seems that most of the outputs are integers but a few are float? How come?

AR: We thank the reviewer for this point. As noted in comment #13, using the refined 3D AL computation, only 28/1157 cases (2%) remain unmeasured due to missing lens segmentations, left/right eye mix-ups, or disconnected lens components. Now, all AL values are float due to sub-voxel interpolation along the 3D line when computing intersections with the segmented structures. With the former method, as the computations were integer based on the voxel size, there was one subject whose voxel size differ from 1mm isotropic, therefore the float for that case.

Comment #32:

It was said that the AL was measured by drawing a line perpendicular to the globe from the centroid of the lens. It is not clear how perpendicular is defined here. Is it based on the surface norm of the globe at the lens, or a line connecting the centroid of lens to the center of the globe?

AR: We thank the reviewer for this question. In the revised 3D AL computation, the line is defined as connecting the centroid of the lens to the centroid of the globe, rather than using the local surface normal. See comment #13. This approach leverages the 3D segmented structures and ensures a consistent and robust measurement of AL across all subjects.
