# Supplementary material for: A-eye: Automated 3D MRI segmentation and morphometric feature extraction for eye and orbit atlas construction
Source: PLoS One. 2026 Jul 2;21(7):e0352257. doi: 10.1371/journal.pone.0352257 (PMC13327317; doi:10.1371/journal.pone.0352257)
Supplement: Attachment — Submitted filename: Response to Reviewers.pdf [file pone.0352257.s015.pdf]

Authors’ Response to Reviews of

J. Barranco et al. – “A-eye: Automated 3D MRI Segmentation and Morphometric Feature Extraction for Eye and Orbit Atlas Construction”

AR: Authors’ Response

We thank the reviewers for their thoughtful comments. We are pleased to submit a revised version of the manuscript, having considered all the received feedback.

In this response, we reproduce all changes made to our submission, marked up with the *Edit* keyword. Page and line numbers of the “Original” text cross-reference the submitted version **without** markup of tracked changes. Page and line numbers of the “Revised” text cross-reference the new document **with** markup of tracked changes. For example:

Edit: **Original (p. N, ll. aaa–bbb):**

> Text in the ~~submitted~~ manuscript—version with~~out~~ track-changes markup,—with ~~deletions marked up by a strike-through line~~.

Revised (p. M, ll. yyy–zzz):

> Text in the revised manuscript—version with track-changes markup,—with additions marked up in blue-color text.

Reviewer #1

Comment #1:

Comment – 1 (Lines 252–253): The dataset includes only manually annotated right eyes, yet the atlas and volumetric benchmarks are applied to the full 1,157-subject cohort without validating generalisation to left eyes. While the authors propose an inversion of the left–right axis to extend the model to the left eye, no empirical evidence is provided to support this approach. Because orbital anatomy exhibits documented left–right asymmetry, with left orbits often 2–8% larger in mean volume, training exclusively on right eyes may introduce systematic bias, especially for fine structures such as the rectus muscles and intraconal fat boundaries. To address this, the authors should include a brief validation on left eyes in the current revision by manually annotating a small sample (approximately 5–10 left eyes) and evaluating performance using standard metrics such as DSC, Hausdorff distance, and volume difference. This step is essential to confirm the robustness and applicability of the atlas and volumetric benchmarks to both eyes.

AR: We thank the reviewer for this valuable comment, which prompted us to carry out additional explorations evaluating the generalization of the segmentation pipeline to left eyes. While we appreciate the suggestion of manually labeling a small number of subjects, we argue that such an

effort would not be justified as proof of generalization. Instead, we segmented the left eye in the full cohort and evaluated the robustness by comparing biometric and volumetric measurements between left and right eyes across the cohort (axial length and total orbital volume). In addition, we complemented this analysis with a qualitative assessment by expert ophthalmologists on a subset of 10 cases randomly selected from the automated segmentation outputs of the left eye.

Edit: **Revised (p. 6, ll. 216–256):**

Validation on left eyes.

To evaluate model performance on left eyes, we conducted dedicated validation using the same cohort. Regarding AL, the proportion of cases where it could not be computed for left eyes (22/1157 = 1.9%) was comparable to that observed for right eyes (28/1157 = 2.42%). Most failures were again due to missing lens visibility in the T1-weighted images (20/22 cases), with the remaining two cases occurring when the lens centroid fell on a non-lens voxel. Among the cases with missing lenses, seven subjects overlapped between left and right eyes, indicating that in these images the lenses were poorly depicted in both eyes. In total, 33 subjects presented missing lenses in at least one eye. After excluding missing values, nnU-Net-derived AL measurements for left eyes (N = 1135) were 22.43 ± 1.47 mm in males (N = 557) and 21.51 ± 1.75 mm in females (N = 578). The within-subject correlation of AL between left and right eyes remained moderate but statistically significant (Pearson's $r = 0.73$, $p < 10^{-10}$). Quantitative comparisons showed minimal inter-eye differences (mean difference -0.16 mm, mean absolute difference 0.65 mm, relative asymmetry -0.81%), indicating strong bilateral symmetry despite the moderate correlation.

To further assess bilateral consistency, we compared left and right eye volumetry within subjects. Total orbital volumes (i.e., the combined volume of all segmented structures) showed a very high correlation (N = 1157, Pearson's $r = 0.96$, $p < 10^{-10}$). To evaluate whether the observed Pearson correlation between left- and right-eye measurements could be explained by random pairing, we performed a permutation test. The right-eye measurements were randomly permuted across subjects 5000 times, thereby disrupting the original within-subject correspondence while preserving the marginal distribution of the data. For each permutation, Pearson's correlation coefficient was recalculated to generate a null distribution under the hypothesis of no within-subject association. As expected, the null distribution was centered near zero. None of the permuted correlations was as large as the observed correlation, yielding an empirical permutation p-value of 2×10^{-4}. These findings indicate that the observed inter-eye correlation is unlikely to have arisen by chance and support the presence of a genuine bilateral association within subjects. Beyond correlation, quantitative comparisons demonstrated low mean absolute differences and small relative asymmetry between eyes across structures, with no relevant systematic bias at the global level. In particular, total orbital volume exhibited minimal inter-eye differences, with a mean absolute difference of 465 mm^3 (0.465 mL) and a relative asymmetry of -1.17% (SD = 4.40%), supporting a high level of bilateral consistency. While small but statistically significant differences were observed for several individual structures, these remained limited in magnitude (typically $< 3\%$) and are likely attributable to a combination of subtle physiological asymmetry and segmentation variability in smaller or less well-defined tissues, such as the lens. Larger structures showed negligible asymmetry and no significant bias, further supporting the robustness of the segmentation. These results are provided in S7 Table.

Additionally, a qualitative evaluation of the segmentation results was also performed on a randomly selected subset of 10 T1w images from the full cohort (N = 1210). A trained ophthalmologist (author AKL) visually assessed all structures for the left eye segmentations. Across all subjects and structures, the mean score was $3.42/4$ (0.86), indicating overall good segmentation quality. Most structures received scores between good and excellent, with the highest average score observed for the globe (3.8). The lowest average score corresponded to the extraconal fat (2.6), which reflects the more diffuse boundaries of this structure in MRI. In one case, the lens segmentation was missing due to the absence of a clearly visible lens in the corresponding T1w image. These results are reported in S8 Table.

Table 1: S7 Table. Bilateral consistency analysis of orbital structures. For each structure, Pearson correlation (r), agreement metrics (mean difference, mean absolute difference, and relative asymmetry), and paired statistical tests are reported. A permutation analysis (shuffled left–right pairing) was performed for total orbital volume to assess whether the observed correlation reflects true within-subject correspondence.

**Structure**	**N**	**r**	**Mean diff** (mm^3)	**MAD** (mm^3)	**Asym.** (%)	**SD asym.** (%)	**p (t-test)**	**p (Wilcoxon)**
Ext. fat	1157	0.95	13.25	242.8	0.59	9.31	0.158	0.018
Globe	1157	0.93	-61.88	179.8	-1.38	6.08	$1.1 \cdot 10^{-15}$	$3.2 \cdot 10^{-18}$
Inf. muscle	1157	0.90	-9.42	37.3	-1.57	8.11	$5.3 \cdot 10^{-11}$	$8.0 \cdot 10^{-11}$
Int. fat	1157	0.94	0.64	174.9	0.03	9.13	0.926	0.548
Lat. muscle	1157	0.84	-15.18	43.3	-3.08	11.08	$1.0 \cdot 10^{-20}$	$3.4 \cdot 10^{-22}$
Lens	1157	0.77	-1.79	12.7	-2.90	33.80	$5.7 \cdot 10^{-3}$	$4.68 \cdot 10^{-2}$
Med. muscle	1157	0.85	-18.35	48.1	-2.90	10.73	$5.5 \cdot 10^{-24}$	$3.0 \cdot 10^{-30}$
Nerve	1157	0.85	-27.12	52.4	-5.42	13.75	$2.6 \cdot 10^{-45}$	$9.6 \cdot 10^{-45}$
Sup. muscle	1157	0.86	-41.02	87.8	-5.36	14.84	$7.1 \cdot 10^{-37}$	$6.8 \cdot 10^{-37}$
Total	1157	0.96	-160.9	465.1	-1.17	4.40	$9.5 \cdot 10^{-20}$	$3.3 \cdot 10^{-19}$

Edit: **Revised (p. 8, ll. 330–340):**

Another limitation is that only right eyes were manually annotated in our dataset. However, we demonstrated that the trained model can be successfully applied to left eyes by reorienting images to a common space, cropping the left-eye region, and mapping the resulting segmentations back to the original space. Quantitative comparisons showed close correspondence between left and right eyes for axial length and total orbital volume, with only small inter-eye differences. These differences were limited in magnitude and likely reflect a combination of subtle physiological variability and segmentation uncertainty, particularly for smaller or less well-defined structures. Consistent with prior cadaveric [81] and CT-based [82] studies reporting high bilateral similarity of orbital volumes, our results support a high degree of left–right correspondence, with only minor differences in healthy individuals and without evidence of a systematic directional bias (i.e., one side being consistently larger than the

Table 2: S8 Table. Qualitative evaluation of left-eye segmentation quality. Mean qualitative scores (0–4 scale) assigned by an ophthalmologist across 10 subjects for each segmented structure. Higher values indicate better segmentation quality; extraconal fat showed the lowest average score due to its diffuse MRI boundaries.

Subject	Lens	Globe	Nerve	Int. fat	Ext. fat	Sup.	Inf.	Lat.	Med.	Overall
2022160100228	4	4	4	3	1	4	3	3	2	3.11
2022160101081	4	4	4	4	3	4	4	4	4	3.89
2022160101099	4	4	3	4	3	3	2	3	3	3.22
2022160101331	4	3	4	4	3	4	4	4	4	3.78
2022160102062	4	4	4	4	2	4	4	3	3	3.56
2022160102540	4	4	4	4	3	4	3	4	4	3.78
2022160102800	0	4	4	3	3	3	4	3	4	3.11
2022160103087	4	4	2	4	3	4	3	4	3	3.44
2022160104234	4	4	4	4	2	3	4	4	4	3.67
2022160104392	2	3	1	3	3	3	4	2	3	2.67
**Mean**	3.4	3.8	3.4	3.7	2.6	3.6	3.5	3.4	3.4	3.42

other). Additionally, a qualitative evaluation performed by an expert ophthalmologist on a subset of 10 subjects yielded high segmentation quality scores, further supporting the robustness of the segmentation pipeline for both eyes.

Edit: **Revised (p. 13, ll. 594–599):**

To segment left eyes, all images were first reoriented to a common RAS (Right-Anterior-Superior) space and the quadrant containing the left eye was cropped prior to inference. The model, trained on right-eye anatomy, was then applied to these cropped left-eye images. The resulting segmentations were mapped back to the original image space via inverse cropping. The total duration of the process for one image takes around 15s. This approach enabled consistent segmentation of left eyes and allowed to merge left and right eye segmentations into a single volume per subject. This facilitates downstream volumetric and biometric analyses.

Edit: **Revised (p. 13, ll. 621–629):**

Evaluation: qualitative validation on left eyes. To qualitatively assess the segmentation performance for both eyes, a subset of 10 T1-weighted (T1w) images was randomly selected from the full cohort (N = 1210). For each subject, the left-eye, right-eye, and merged (left–right) segmentations produced by the model were visually evaluated by one of our ophthalmologist coauthors (AKL). Each segmented structure (lens, globe, optic nerve, intraconal fat, extraconal fat, superior rectus, inferior rectus, lateral rectus, and medial rectus) was scored

using a 5-point ordinal scale: 0 = exclude, 1 = poor, 2 = acceptable, 3 = good, and 4 = excellent. The reviewer was also invited to provide comments regarding segmentation quality or potential artifacts. The evaluation focused on anatomical plausibility, boundary accuracy, and consistency with the underlying MRI appearance.

Edit: **Revised (p. 22, l. 1123–1125):**

S8 Table. Qualitative evaluation of left-eye segmentation quality. Mean qualitative scores (0–4 scale) assigned by an ophthalmologist across 10 subjects for each segmented structure. Higher values indicate better segmentation quality; extraconal fat showed the lowest average score due to its diffuse MRI boundaries.

Comment #2:

Comment – 2 (Lines 269–274): The authors correctly highlight the limitations of generic brain-focused MRI-QC tools for eye-specific imaging, which is an important observation. However, the proposed QC protocol lacks formal quantitative validation and inter-rater reliability assessment. The custom 0–4 quality rating scale does not define objective criteria for each level (e.g., measurable noise thresholds, blur quantification, or motion artifact detection), and no inter-rater agreement statistics (Cohen's kappa or ICC) are reported for the two raters mentioned. Additionally, the relationship between QC scores and segmentation performance has not been fully validated across the entire cohort; the correlation observed for 43 test images may not generalise. Addressing these points would strengthen the reliability and applicability of the proposed QC framework.

AR: We thank the reviewer for this constructive comment. We agree that quantitative validation and clearer documentation of the QC protocol strengthen the reliability of the framework. Following the reviewer's suggestion, we assessed inter-rater reliability of the QC ratings, which showed moderate-to-good agreement between the two raters. We also clarify that the analysis relating QC scores to segmentation performance was performed on the test set only and should therefore be interpreted with caution when considering the full cohort. Finally, to improve transparency and reproducibility, we have made the QC evaluation protocol and annotation guidelines available as supplementary material.

Edit: **Original (p. 5, ll. 155–156):**

~~The correlations were near zero, suggesting that the model performance was not strongly influenced by image quality~~.

Revised (p. 5, ll. 153–157):

The correlation analysis showed weak associations between DSC and subjective image quality across all structures (all $|r| \leq 0.25$; see S2 Fig.) and no statistically significant correlations (all $p > 0.05$), indicating no clear monotonic relationship within the test set and suggesting that any potential association is likely weak within the observed quality range. Due to the limited sample size ($N = 43$), this analysis may be underpowered and should be interpreted with caution, and may not generalize to the full cohort.

Edit: **Revised (p. 8, ll. 351–358):**

To ensure the removal of low-quality images that could compromise the results, we introduced QC protocols at multiple stages of the segmentation pipeline. Inspired by the state-of-the-art method MRI-QC [38], we observed a mismatch between low-quality images identified by the MRI-QC toolbox and those identified by our MR-Eye experts. This suggests that QC in MR-Eye requires different metrics and criteria compared to brain imaging. The moderate-to-good inter-rater agreement obtained for the QC ratings supports the consistency of the proposed visual QC protocol. Nevertheless, future work should aim to develop objective quantitative metrics tailored specifically to orbital MRI, incorporating non-tissue metrics and extending scrutiny to the periorbital region to further standardize image quality assessment.

Edit: **Revised (p. 12, ll. 549–564):**

To obtain the subjective eye-quality of the 43 images in the test set, two engineer experts in ophthalmic MR image analysis (20 and 5 years of experience) independently evaluated image quality using a structured visual quality control (QC) protocol adapted from MRIQC reports [38]. These reports consist of an HTML file per subject presenting multiple axial thumbnails as well as sagittal and coronal views to assist visual inspection. A rating widget was provided including several components to evaluate specific image artefacts such as blur, noise, motion, and background air artefacts. We modified the original MRIQC reports to better suit orbital imaging by centering the thumbnails on the right eye and adding eye-specific aspects to the rating interface, such as eye open/closed status (Fig. 7).

The evaluation followed a guided workflow in which raters first assessed individual image artefacts using dedicated sliders and then assigned an overall quality score on a 0–4 scale (0 = excluded, 1 = poor, 2 = acceptable, 3 = good, 4 = excellent). The final rating therefore reflected both the presence of artefacts and the visibility of relevant orbital structures, including external structures (globe and lens) and internal structures (optic nerve and extraocular muscles), evaluated across the three orthogonal views of the 3D MRI volume. Inter-rater reliability for the overall QC rating showed moderate-to-good agreement between raters (ICC(2,1) = 0.71, 95% CI: 0.54–0.81). Detailed QC annotation guidelines used by the raters are provided in S10 File to improve transparency and reproducibility of the evaluation protocol.

Edit: **Revised (p. 22, l. 1130-1132):**

S10 File. MR-Eye Quality Control Annotation Guidelines. Guidelines used by raters for the subjective quality control (QC) evaluation of eye MRI images, including rating criteria, artefact assessment workflow, and instructions for assigning overall image quality scores.

Comment #3:

Comment – 3 (Lines 174–182, 280–287): Axial length (AL) extraction failed in 2% of cases (28/1,157), primarily due to lens segmentation errors, but the manuscript does not systematically characterise these failures or assess performance in pathological eyes. While Figure 3A shows moderate-to-high correlation with manual references on 43 subjects, this limited analysis does not provide full insight into reliability across the cohort. The authors should report sensitivity and specificity for lens detection from T1-weighted images and examine the relationship between image quality scores and segmentation success. Additionally, performance in pathological eyes

> *(e.g., cataracts, post-surgical changes, lens dislocation) should be discussed, as these conditions may reduce contrast and affect segmentation. The relatively high rate of absent cornea detection (17% of eyes imaged open) also indicates sensitivity to anatomical variability and warrants further explanation. Addressing these points would strengthen confidence in the AL extraction pipeline.*

AR: We thank the reviewer for this important comment about the axial length extraction. We clarified that most AL extraction failures were primarily related to image limitations rather than instability of the segmentation model. Following the reviewer's suggestion, we also refined the interpretation of lens detection performance. We further acknowledge that the correlation with manual references was evaluated on the 43 test subjects and does not directly assess the full cohort. Nevertheless, the large-scale cohort results are consistent with previously reported population values, supporting the reliability of the automated AL estimates at the cohort level. Finally, we corrected and clarified the reporting regarding cornea detection and its limitations in MRI.

Edit: **Revised (p. 5, ll. 176–186):**

> However, in 28/1157 (2.42%) cases, after manual inspection, the AL could not be computed due to: (1) 20 missing lens segmentations, typically caused by poor visibility or absence of the lens on the T1w images; (2) 7 instances where the left eye was segmented instead of the right; and (3) 1 case where the lens centroid did not fall within any lens voxel, due to disconnected components in the segmentation (no post-processing applied after inference). To evaluate potential bias introduced by the remaining 28 cases with missing AL measurements, we analyzed their demographic and image-quality characteristics. This subset included 19 males and 8 females, with a mean age of 63.5 $\pm$ 11.8 years and a mean BMI of 30.5 $\pm$ 4.4 kg/m^2. Image quality was generally lower than average, often showing left–right phase-encoding artifacts near the eyes. Overall, these characteristics are comparable to those of the complete cohort, indicating that the small number of missing AL cases is unlikely to affect the overall volumetric or atlas results. Lens detection showed a high success rate (1137/1157, 98.3%). These findings indicate that most AL extraction failures were driven by image limitations rather than instability of the segmentation model.

Edit: **Revised (p. 9, ll. 360–378):**

> To further evaluate the pipeline, we implemented an automated method to estimate AL from the segmented MR-Eye volumes. The automated measurements showed good agreement with reference AL values reported in the literature and with manual measurements obtained in the test set of 43 subjects. In the large-scale cohort, AL could not be computed in 2% of cases (28/1157), primarily due to missing lens segmentations caused by poor visibility or absence of the lens in the T1-weighted images, occasional laterality selection errors, or disconnected segmentation components. These failures suggest that most limitations were related to image characteristics rather than instability of the segmentation model. Potential mitigation strategies include quadrant eye segmentation (as we do for the left eyes), connected-component post-processing, and stricter QC procedures to exclude images with insufficient lens visibility.
>
> From a clinical perspective, several ocular conditions are unlikely to substantially degrade MRI-based lens detection. Cataract-related lens opacification is defined by optical transparency

and does not necessarily reduce MRI contrast; previous studies have even reported increased lenticular MRI signal intensity with age [84], which may facilitate lens delineation. Likewise, in post-surgical eyes such as pseudophakia, the interface between the implant or residual capsule and the vitreous body typically remains sufficiently visible to define lens boundaries. However, severe anatomical alterations, such as lens dislocation, major orbital trauma, or vitreous substitutes (e.g., gas or silicone oil), may still pose challenges for automated segmentation and warrant further investigation. Nevertheless, these cases are often present as acute clinical emergencies where MRI is not the primary diagnostic modality. Finally, the cornea is a very thin structure ($\sim$550 μm), below the spatial resolution of typical MRI acquisitions ($\sim$1 mm), which explains why its detection can be sensitive to acquisition conditions and is facilitated when images are acquired with the eyes closed.

Edit: **Original (p. 10, ll. 433–436):**

When the cornea could not be detected ~~(198/1157 cases, 17%)~~, usually due to ~~open eyes~~, the anterior corneal distance was defined as the median value observed in subjects with eyes closed ~~(83%)~~. The total AL is then defined as the distance from the cornea to the posterior pole of the globe.

Revised (p. 14, ll. 642–650):

When the cornea could not be detected (147/1210≈12.1% of right eyes and 104/1210≈8.6% of left eyes), the anterior corneal distance was defined as the median value observed in subjects with eyes closed. Based on visual inspection of representative failure cases, these non-detections were mainly associated with limited visibility of the anterior eye region in the T1w images, for example when the eyes were open or not fully closed, when the lens was poorly visible or missing, or when image quality around the lens–cornea transition was insufficient for reliable boundary identification. This reflects the limited MRI visibility of the cornea, which is a thin structure ($\sim$0.55 mm) below the spatial resolution of the images used in this study, rather than instability of the segmentation model itself. The total AL is then defined as the distance from the cornea to the posterior pole of the globe.

Comment #4:

Comment – 4 (Lines 188–191, 295–302): The study reports minimal correlation between BMI and eye structure volumes ($R^2 < 0.1$), which contrasts with prior findings linking BMI to exophthalmometry (Reference [42], $p < 0.001$). The explanation invoking head size or body height is speculative, as these variables were not measured in the current cohort. For transparency, the authors should report the correlation coefficients (e.g., Pearson's r) and their associated p-values. If head size or body height are suggested as confounders, they should be clearly framed as hypotheses rather than definitive explanations. Additionally, the manuscript should discuss the phenotypic differences between volumetric measurements and exophthalmometry to contextualise the discrepancy with prior studies. Addressing these points would improve interpretability and strengthen the conclusions regarding BMI-related effects.

AR: We thank the reviewer for this suggestion. We computed Pearson correlation coefficients and corresponding p-values for the association between BMI and orbital structure volumes, confirming statistically significant but weak-to-moderate effect sizes, indicating limited explanatory power of

BMI. Following Reviewer 2.6's suggestion, we also performed adjusted regression analyses including age and body height as covariates, which confirmed that BMI remains independently associated with most structures, although with modest effect sizes. We further clarified the interpretation of these findings by highlighting the distinction between volumetric MRI measurements and exophthalmometric assessments, which capture different anatomical features. The manuscript has been revised accordingly to improve clarity and interpretability of these results.

Table 3: S6 Table. Pearson correlation between BMI and orbital structure volumes.

Structure	N	r	p-value	p_{FDR}
Lateral rectus muscle	1156	0.379	1.02×10^{-40}	9.15×10^{-40}
Optic nerve	1156	0.334	1.82×10^{-31}	8.17×10^{-31}
Superior rectus muscle	1156	0.306	1.93×10^{-26}	5.79×10^{-26}
Medial rectus muscle	1156	0.261	1.79×10^{-19}	4.03×10^{-19}
Intraorbital fat	1156	0.256	9.37×10^{-19}	1.69×10^{-18}
Extraorbital fat	1156	0.176	1.64×10^{-9}	2.45×10^{-9}
Globe	1156	0.136	3.54×10^{-6}	4.56×10^{-6}
Inferior rectus muscle	1156	0.122	3.34×10^{-5}	3.76×10^{-5}
Lens	1156	0.111	1.55×10^{-4}	1.55×10^{-4}

Edit: **Revised (p. 5, ll. 197–205):**

> Interestingly, robust linear regression (Huber) showed low coefficients of determination ($R^2 < 0.1$ in most cases), indicating limited explanatory power of BMI. Fig. 5 illustrates these relationships with scatter plots and Huber regression lines, grouped by sex. To account for potential confounding factors, we further performed adjusted robust regression analyses including age and body height as covariates. BMI remained significantly associated with most orbital structure volumes in both sexes (all FDR-corrected $p < 0.05$, except for lens volume in males), although effect sizes remained modest (see S5 Table). Consistent with these findings, Pearson correlation analysis revealed small to moderate effect sizes ($r = 0.11$–0.38, all $p < 0.001$ after Benjamini–Hochberg correction), corresponding to low explained variance ($R^2 < 0.15$ across all structures, see S6 Table). These results indicate that BMI has an independent but limited contribution to the variability of orbital tissue volumes.

Edit: **Revised (p. 10, ll. 415–431):**

> Interestingly, although previous work [42] reported a significant association between exophthalmometry (defined as the perpendicular distance between the interzygomatic line and the posterior surface of the cornea) and both axial length and BMI ($p < 0.001$), our investigation revealed only weak—albeit statistically significant—associations between BMI and eye structure volumes. This suggests that BMI explains only a limited proportion of the variability in orbital tissue volumes. One possible explanation for this discrepancy lies in the different phenotypes being measured. Exophthalmometry quantifies the anterior displace-

ment of the globe relative to the lateral orbital rim and is therefore strongly influenced by the capacity and geometry of the bony orbit. In contrast, our MRI-based volumetric analysis directly measures intraorbital soft tissues. As a result, increases in orbital fat volume (e.g., Grave's disease) may be partially accommodated by orbital architecture and not necessarily translate into measurable proptosis. By focusing on tissue volume rather than globe position, our approach captures a distinct anatomical phenotype, which may explain the weaker associations observed. We further accounted for potential confounding factors by including age and body height in adjusted regression models, which confirmed that BMI has an independent but modest association with orbital volumes. Although head or orbital size could also influence these relationships, we did not include a head-size covariate in the final models. Automated MRI-derived head masks showed variability across subjects (e.g., due to differences in field-of-view and head segmentation consistency), limiting their reliability as a covariate. Moreover, global head size represents only an indirect proxy for orbital capacity. Future work incorporating direct measures of orbital or craniofacial anatomy may help further clarify these relationships.

Comment #5:

Comment – 5 (Lines 311–314): The authors acknowledge that the cohort is predominantly ethnically homogeneous, but their discussion of cross-ethnic comparability relies on general statements rather than sufficient evidence. The claim that key ocular anatomical features are largely comparable across ethnicities, with only minor differences, requires stronger support. Specifically, the manuscript does not report the ethnic or racial composition of the SHIP cohort, nor does it critically evaluate the cited reference [85]. Moreover, recent ophthalmologic studies document clinically significant ethnic differences in axial length, orbital volume, and myopia prevalence, which may affect the generalizability of the atlas. The authors should provide clearer demographic reporting, evaluate relevant literature, and explicitly discuss potential limitations arising from ethnic homogeneity.

AR: We thank the reviewer for this important comment. We have revised the manuscript to explicitly report the ethnic composition of the SHIP cohort (>99% Caucasian). The discussion now acknowledges documented ethnic differences in ocular biometry and clarifies that the presented atlas reflects a predominantly Caucasian phenotype, whose generalizability requires validation in multi-ethnic cohorts.

Edit: **Revised (p. 10, ll. 442–453):**

The SHIP cohort is representative of the population in Northeastern Germany and consists of more than 99% individuals of Caucasian descent. While this provides a robust anatomical reference for European populations, it limits the generalizability of our findings to other ethnic groups. Previous studies have reported clinically relevant ethnic differences in ocular biometry, including axial length, orbital volume, and the prevalence of myopia, particularly in East Asian populations [93]. These differences may influence both absolute volumetric measurements and the relative proportions of orbital structures. At the same time, studies based on external eye morphology derived from photographic analyses have suggested that several global shape descriptors (e.g., eye height, length, and ellipticity) are relatively consistent across ethnicities, with variability often dominated by inter-individual differences rather than ethnic group effects [94]. However, such findings primarily reflect surface anatomical features and may

not directly translate to internal volumetric characteristics assessed by MRI. Therefore, the atlas presented in this work should be interpreted as reflecting a predominantly Caucasian phenotype. Its applicability to other populations remains to be established and warrants validation in multi-ethnic cohorts.

Edit: **Revised (p. 11, ll. 493–496):**

The cohort was originally acquired as part of the Study of Health in Pomerania (SHIP) [40, 41, 42] and reused for the present study. Whole-body MRI data was obtained from 3030 adult participants drawn from the SHIP-2 and SHIP-Trend cohorts. The SHIP study is a population-based cohort from Northeastern Germany and is demographically representative of this region, with more than 99% of participants of Caucasian (European) descent [41].

Comment #6:

Comment – 6 (Lines 391–399): While the manuscript provides extensive hyperparameter details, it lacks justification for architecture-specific choices, particularly the patch size [128, 160, 112], and does not include ablation studies to support these decisions. Using default nnU-Net parameters with minimal modification leaves critical questions unanswered. Specifically, the authors should clarify why this patch size was selected and whether performance is sensitive to different patch sizes (typical ranges: [64, 64, 64] to [256, 256, 256]). The choice of five-fold cross-validation should also be justified, as a higher fold number (e.g., 10-fold) could better estimate variance. Additionally, the suitability of the data augmentation strategies (scaling, rotation) for fine structures such as the optic nerve and rectus muscles should be discussed. Finally, including training curves (loss, DSC over epochs) would help assess potential overfitting and provide insight into model convergence.

AR: We thank the reviewer for this important comment. The nnU-Net framework automatically determines architecture and training parameters based on dataset characteristics, avoiding manual hyperparameter tuning. We followed the standard nnU-Net protocol with five-fold cross-validation and evaluated performance on an independent test set. Data augmentation was limited to anatomically plausible transformations, and no ablation study was performed, consistent with nnU-Net's design. Training curves have now been added to the supplementary material to document convergence.

Edit: **Revised (p. 12, ll. 572–592):**

NnU-Net [60] is the state-of-the-art supervised deep learning-based segmentation approach in which data augmentation is extensively used and the hyperparameters are automatically optimized. It has never been evaluated for MR-Eye, but with OCT [97]. We split the manual annotated dataset into 31 for training and 43 for testing. The split reflected data availability. Initially, 35 subjects were available, 31 were used for training / validation, and 4 for testing. After receiving 39 additional segmentations, the 4 initial test cases were added to this larger cohort to increase the test set (43 in total).

All hyperparameters were determined using the default nnU-Net experiment planning pipeline without manual tuning. In particular, the patch size [128, 112, 160], batch size (2), and network topology were automatically derived from the image spacing (1 mm isotropic), median image size (176 $\times$ 256 $\times$ 176 voxels), and available GPU memory. The resulting configuration

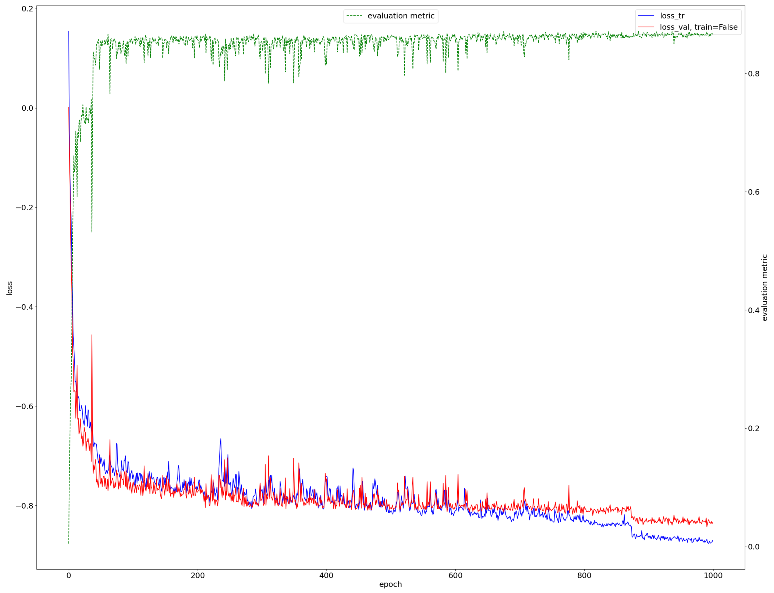

Figure 1: S11 Fig. Training and validation loss curves (blue and red, respectively) and validation Dice score (green dashed line) across epochs for nnU-Net training. The curves show stable convergence, with decreasing loss and consistent improvement of the Dice score, indicating no evident overfitting.

consisted of a single 3D full-resolution stage with five-fold cross-validation during training. Training was performed using an initial learning rate of 0.001 with a ReduceLROnPlateau scheduler, the ADAM optimizer, and deep supervision with a combined cross-entropy and Dice loss function. Data augmentation followed the default nnU-Net strategy, including scaling and rotation within anatomically plausible ranges to improve generalization while preserving the structural integrity of fine orbital anatomy. Kaiming-He (0.01) weight initialization was used, and no postprocessing was applied after inference. Training was performed for up to 1000 epochs, with an elapsed time of approximately 140–170 seconds per epoch. The model included 10 classes (9 ROIs plus background). Computations were carried out on an HPC (High Performance Computing) SLURM-based cluster using GPUs (RTX 2080 and RTX 3090), 10 CPUs per fold, and 64 GB RAM, within Docker containers accessed via Singularity, using PyTorch and Python 3.8. The total training time for the five folds was approximately 208 h 20 min. The inference time was approximately 1 minute per image. Processing the full non-labeled dataset (1157 subjects) required 66,185.53 seconds (18 h 23 min 05 s) using an RTX 3060 Ti GPU.

Comment #7:

Comment – 7 (Lines 443–445): Volumetry is reported at a voxel resolution of 1 mm³, but the manuscript does not provide measurement precision, confidence intervals, or test-retest reliability, limiting the assessment of clinical utility. While Table 3 presents median ± standard deviation, important metrics are missing, including coefficient of variation (CV%), 95% confidence intervals, and repeatability data. Additionally, clinically meaningful thresholds for orbital pathologies are not discussed. For example, extraconal fat volume shows high variability (±1,134 mm³ in males, 31% CV), raising concerns about the reliability of these measurements for diagnostic or clinical purposes. Including these metrics would strengthen the manuscript by demonstrating both precision and potential clinical applicability of the volumetric data.

AR: We thank the reviewer for this insightful comment. We have expanded the reported metrics to provide a more comprehensive description of variability, including additional dispersion measures and confidence intervals. These results show that, although some structures exhibit higher variability, the estimates remain stable at the population level given the large sample size. We acknowledge that repeatability could not be assessed in this dataset, as only one segmentation per subject was available, and clarify that the focus of this study is on population-level benchmarks. Finally, we clarify that the reported measurements should be interpreted as reference distributions rather than diagnostic thresholds, with future work needed to establish clinical cut-offs in patient cohorts.

Edit: **Revised (p. 5, ll. 188–195):**

> In terms of volumetry extraction, we provide the first large-scale benchmark MR-Eye volumetry of all eye structures. Descriptive statistics, including mean ± standard deviation, median with interquartile range, coefficients of variation (CV%), and 95% confidence intervals, are reported in Table 3. Unadjusted analyses showed significantly larger volumes in males compared to females for all structures except the lens (all FDR-corrected $p < 0.05$), with small-to-moderate effect sizes (see S3 Table). After adjustment for body height (and age), several sex differences remained significant, particularly for intra- and extraconal fat and the globe, whereas others were attenuated, indicating that part of the observed differences is explained by anthropometric variability (see S4 Table). Fig. 4 illustrates the distribution of volumetric measurements per structure, grouped by sex, using violin plots from the large-scale cohort of 1,157 subjects.

Edit: **Revised (p. 9, ll. 395–413):**

> We also provide large-scale benchmarks for volumetry of all eye structures at a millimeter scale. Compared with previous studies reporting volumetric measurements for selected structures in cm^3 [45, 46, 47], our work extends these findings by providing a comprehensive and detailed characterization of all major orbital compartments in a large cohort. While unadjusted analyses showed generally larger volumes in males compared to females across most structures, these differences were attenuated after adjustment for body size, indicating that they are partly explained by anthropometric variability. Nevertheless, several structures—particularly intra- and extraconal fat—remained significantly different after adjustment, suggesting that both body size and intrinsic anatomical differences contribute to sex-related variability. Importantly, effect sizes were modest, indicating that sex alone explains only a limited proportion of volumetric variability. This sex-wise differentiation in eye structure volumetry could have relevant implications for understanding sex-specific

Table 4: Table 3. Volumetric measurements of orbital structures by sex. Values are reported as mean $\pm$ standard deviation (SD), median with interquartile range (IQR), 95% confidence intervals (CI), and coefficient of variation (CV).

Structure	Sex	Mean $\pm$ SD (95% CI mean)	Median [IQR] (95% CI median)	CV (%)
Lens	Male	106.16 $\pm$ 33.38 (103.41, 108.91)	110.00 [93.00–127.00] (107.00, 113.00)	31.44
Lens	Female	105.72 $\pm$ 31.95 (103.13, 108.31)	111.00 [91.00–128.00] (109.00, 113.00)	30.22
Globe	Male	5218.60 $\pm$ 1086.09 (5129.09, 5308.11)	5327.50 [4922.25–5752.75] (5263.50, 5395.00)	20.81
Globe	Female	5020.03 $\pm$ 704.02 (4963.06, 5077.01)	5015.00 [4637.00–5398.00] (4969.00, 5094.00)	14.02
Optic nerve	Male	616.00 $\pm$ 136.44 (604.76, 627.25)	626.00 [558.00–691.25] (617.00, 639.00)	22.15
Optic nerve	Female	581.57 $\pm$ 95.46 (573.84, 589.29)	583.00 [524.00–638.00] (574.00, 592.00)	16.42
Intraconal fat	Male	2657.43 $\pm$ 839.44 (2588.24, 2726.61)	2660.00 [2149.75–3152.75] (2576.00, 2746.00)	31.59
Intraconal fat	Female	2164.31 $\pm$ 612.88 (2114.71, 2213.91)	2125.00 [1756.00–2559.00] (2040.00, 2188.00)	28.32
Extraconal fat	Male	3751.62 $\pm$ 1134.92 (3658.09, 3845.15)	3703.00 [3026.75–4451.50] (3602.00, 3832.00)	30.25
Extraconal fat	Female	2687.78 $\pm$ 752.32 (2626.89, 2748.66)	2609.00 [2146.00–3138.00] (2552.00, 2678.00)	27.99
Lateral RM	Male	553.20 $\pm$ 123.65 (543.01, 563.39)	557.00 [493.50–626.00] (548.00, 567.00)	22.35
Lateral RM	Female	502.53 $\pm$ 82.95 (495.82, 509.24)	500.00 [447.00–548.00] (494.00, 508.02)	16.51
Medial RM	Male	719.08 $\pm$ 143.29 (707.27, 730.89)	725.50 [653.00–794.00] (714.00, 738.00)	19.93
Medial RM	Female	679.70 $\pm$ 104.06 (671.28, 688.13)	677.00 [626.00–740.00] (667.00, 686.00)	15.31
Inferior RM	Male	667.28 $\pm$ 139.46 (655.79, 678.77)	671.00 [609.00–751.25] (662.00, 680.50)	20.90
Inferior RM	Female	604.96 $\pm$ 97.51 (597.07, 612.86)	604.00 [546.00–667.00] (595.00, 615.00)	16.12
Superior RM	Male	904.34 $\pm$ 255.13 (883.31, 925.36)	907.50 [782.00–1052.00] (884.00, 935.00)	28.21
Superior RM	Female	787.05 $\pm$ 193.12 (771.42, 802.68)	770.00 [651.00–910.00] (751.00, 792.00)	24.54

ophthalmological conditions and tailoring more personalized medical treatments, particularly as such differentiation is nowadays needed for a better health care [58, 59]. The reported measurements also highlight differences in variability across structures, with higher coefficients of variation observed for fat tissues compared to more compact structures such as the globe or optic nerve. This reflects the diffuse anatomical nature of orbital fat and its greater inter-individual variability. From a clinical perspective, the volumetric values reported here should be interpreted as reference distributions rather than diagnostic thresholds. Absolute quantification of certain tissues, particularly orbital fat, may be less informative than relative changes, asymmetry, or patterns of tissue expansion within the orbital cavity. Future work could apply the proposed automated segmentation framework to patient cohorts and compare volumetric measurements with healthy controls, which may be particularly relevant for conditions such as Graves' orbitopathy and other orbital pathologies.

Comment #8:

8. Interpretation and thresholding of atlas probability maps.

AR: We thank the reviewer for this comment. As no additional details were provided, we understand that it refers to the interpretation and use of the atlas probability maps mentioned in the editorial summary; this is addressed in comment R2.4. We have clarified the interpretation and usage of the probability maps, emphasizing that they represent voxel-wise label frequencies and encode anatomical uncertainty. We also clarify that the atlas labels correspond to maximum-probability assignments, while the probability maps are intended for flexible use depending on the application.

Edit: **Revised (p. 14, ll. 687–696):**

> To generate the atlas labels, we first registered each subject to the corresponding atlas space (male or female) and projected the nnU-Net segmentations accordingly. The overall process required approximately 25 minutes for the male cohort and 39 minutes for the female cohort. We then constructed a maximum-probability atlas using majority voting across subjects. In addition, we explicitly represented uncertainty in the atlas labels through probability maps, which we provide alongside the atlas. These maps encode the voxel-wise frequency of label occurrence across the cohort after spatial normalization, thereby reflecting the degree of consensus (or uncertainty) for each anatomical structure. This probabilistic representation complements the maximum-probability labels and allows identification of regions with higher uncertainty, such as diffuse anatomical boundaries (e.g., extraconal fat). For visualization purposes, we color-coded the probability maps by modulating label-specific intensities according to voxel-wise probabilities, such that lower-probability regions appear less saturated. The eye atlases can be downloaded at [61].

Comment #9:

Comment – 8 (Lines 247–250): The study relies on a single manual segmentation per subject, preventing assessment of inter-rater reliability via Intraclass Correlation Coefficient (ICC), as performed in prior comparative studies (Reference [20]). This is a significant methodological limitation that affects the reliability of the "ground truth" annotations. Without independent rater assessments, the annotations may reflect individual rater bias, particularly for structures with high anatomical variability, such as rectus muscles and orbital fat, where inter-rater disagreement is typically 5–15% in the literature. Consequently, it is difficult to determine whether the reported nnU-Net performance (DSC = 0.80 ± 0.07) represents optimal segmentation accuracy or merely falls within the expected range of human variability. Acknowledging and discussing this limitation is important for contextualising model performance.

AR: We thank the reviewer for this important comment. We agree that the absence of independent multi-rater annotations prevents assessment of inter-rater agreement and limits comparison with human variability. Annotations in this study were reviewed by a senior ophthalmologist (OL), resulting in an expert-curated reference. Accordingly, the reported nnU-Net performance should be interpreted as agreement with this reference. This limitation has now been clarified in the Discussion.

Edit: **Revised (p. 8, ll. 320–328):**

> Additionally, it would be valuable to assess inter-rater variability by including multiple

independent manual annotations, as in [20], where inter-rater agreement was quantified via ICC. However, in our study only a single expert-curated annotation per subject was available, which prevented such an analysis. We acknowledge this limitation; however, prior studies on MRI-based orbital segmentation report moderate-to-good inter-rater agreement (ICC $\approx$ 0.5–0.8), particularly for structures with less well-defined boundaries such as extraocular muscles and orbital fat [79, 80]. In this context, the reported segmentation performance (DSC = 0.80 $\pm$ 0.07) is consistent with the level of variability typically observed in expert annotations. We consider this an important direction for future work to further contextualize the robustness of automated segmentation relative to human variability.

Reviewer #2

Comment #10:

1. Critically Small Training Set With No External Validation. The model was trained on only 31 subjects. No external validation on independent data (different scanner, field strength, or population) was performed. The generalizability claims are therefore entirely unsupported. The authors evaluate a 10-class 3D segmentation with 31 training cases and a 43-case test set, and performance claims are then extended to a 1,000+ subject cohort and atlas construction. This is an unusually small training set for the complexity of the task and raises a high risk of dataset-specific learning.

AR: We thank the reviewer for this important comment. We agree that the training set (31 subjects) is modest for a 10-class 3D segmentation task and that no external validation on independent data was performed. We have revised the manuscript to avoid overstating generalizability. The presented results should be interpreted as an internal proof-of-concept demonstrating that the nnU-Net framework can learn anatomically meaningful segmentations within this dataset. Manual multi-structure annotation of orbital MRI is highly labor-intensive, which limited the size of the training set. To mitigate overfitting, we used the standard nnU-Net framework with data augmentation, five-fold cross-validation during model development, and evaluation on a held-out test set (N = 43). However, we agree that this does not replace external validation. This limitation is now stated more explicitly in the Discussion, and future work will focus on validation across independent multi-site datasets. The proposed web platform is intended to facilitate such future evaluations.

Edit: **Revised (p. 8, ll. 309–318):**

While the validation cohort of 43 subjects is modest compared to the overall dataset, the automated segmentations applied to the remaining 1,157 subjects produced axial length distributions consistent with state-of-the-art manual measurements reported in the ophthalmological literature [22, 42], supporting the robustness of our findings within this dataset. However, the relatively small number of manually annotated cases used for training and the absence of external validation across different scanners, field strengths, or populations remain important limitations of our study. Therefore, the nnU-Net results should be interpreted as an internal proof-of-concept rather than evidence of full generalizability. In this context, the proposed web platform provides a practical framework for future validation by enabling the application and evaluation of the model on data acquired with different scanners, contrasts,

and imaging protocols, thereby supporting the assessment of generalizability in multi-center settings.

Comment #11:

2. Right-Eye-Only Design Severely Limits Utility. Right-eye-only labelling and atlas construction severely limit the usefulness and generalizability of the proposed framework. Left-eye generalization is merely hypothesized but never tested, despite known subtle orbital asymmetries.

AR: We thank the reviewer for raising this important point. This concern overlaps with Reviewer 1 Comment 1, where we have now provided a detailed evaluation of the applicability of the segmentation framework to left eyes. In brief, we implemented an inference strategy enabling the model trained on right-eye annotations to segment left eyes via reorientation to a common space and region-specific cropping. Using this approach, we computed axial length and volumetric measurements for the left eyes across the full cohort. The results demonstrate strong bilateral consistency, with high correlations between left and right eyes (Pearson's $r = 0.73$ for axial length and $r = 0.96$ for total orbital volume), as well as low inter-eye differences in agreement analyses. In addition, a qualitative evaluation by an expert ophthalmologist on a subset of cases confirmed overall good segmentation quality. Regarding the atlas, it was intentionally constructed using right-eye annotations to ensure anatomical consistency during template generation. Extending the atlas to explicitly include left-eye representations is a relevant direction for future work. The manuscript has been updated accordingly, and we refer the reviewer to the detailed additions provided in Reviewer 1 Comment 1.

Comment #12:

3. Single-Rater Ground Truth Makes All Metrics Uninterpretable. All annotations were produced by one junior ophthalmologist (1 year of experience) and reviewed by a senior expert. Without independent multi-rater annotations, there is no inter-rater variability baseline. This fundamentally undermines every quantitative claim in the paper.

AR: We thank the reviewer for emphasizing the importance of inter-rater variability and agree that multi-rater annotations would provide a valuable baseline. However, we respectfully disagree that the use of a single curated reference "undermines every quantitative claim". The annotations were reviewed by a senior ophthalmologist, yielding a consistent expert-defined reference, which is standard practice in medical image segmentation. We acknowledge that the absence of independent raters limits comparison to human variability, and this has now been clarified in the revised manuscript and in comment Reviewer 1 Comment 8. We have also further clarified the manual annotation procedure in the Methods section of the manuscript (dataset description) to improve transparency.

Edit: **Revised (p. 8, ll. 320–328):**

> Additionally, it would be valuable to assess inter-rater variability by including multiple independent manual annotations, as in [20], where inter-rater agreement was quantified via ICC. However, in our study only a single expert-curated annotation per subject was available, which prevented such an analysis. We acknowledge this limitation; however, prior studies on MRI-based orbital segmentation report moderate-to-good inter-rater agreement (ICC $\approx$ 0.5–0.8), particularly for structures with less well-defined boundaries such as extraocular muscles and orbital fat [79, 80]. In this context, the reported segmentation performance

(DSC = 0.80 ± 0.07) is consistent with the level of variability typically observed in expert annotations. We consider this an important direction for future work to further contextualize the robustness of automated segmentation relative to human variability.

Edit: **Revised (p. 12, ll. 541–546):**

Manual annotations on a total of 74 subjects were done, using ITK Snap software [95], by two ophthalmologists: one senior (20 years of experience) and one junior (1 year of experience). The first batch of 35 subjects was annotated by PS, and the remaining 39 by AKL. The senior ophthalmologist (OL), reviewed all annotations and corrected them when necessary, ensuring consistency and quality control across the dataset.

Comment #13:

4. Unvalidated Atlas Inherits Systematic Model Errors. Atlas label generation is based on projecting automated nnU-Net segmentations and majority voting. This means the atlas will inherit systematic model errors (e.g., weaker performance in extraconal fat/rectus muscles, laterality mix-ups, missing lens segmentations). Yet, the manuscript provides no convincing atlas-validation framework (e.g., manual-label evaluation in atlas space, registration-quality/sharpness metrics, or uncertainty quantification tied to label fidelity). Without that, the atlas cannot be considered a reliable reference standard.

AR: We thank the reviewer for this important comment. We emphasize that the atlas is constructed from a large cohort ($N = 1210$) using majority voting, which reduces the impact of isolated segmentation errors. In addition, probability maps are provided to explicitly represent voxel-wise uncertainty and complement the maximum-probability labels. To further assess atlas reliability, we performed an additional validation by comparing the atlas labels with a manual atlas segmentation. This analysis showed good agreement for most structures, particularly well-defined ones (see S9 Table). Overall, the combination of large-cohort aggregation, uncertainty representation, and additional validation supports the reliability of the proposed atlas. The manuscript has been revised accordingly.

Edit: **Revised (p. 7, ll. 266–270):**

The atlas validation indicated overall good agreement between the automatically generated labels and the manual atlas segmentation (see S9 Table). Similarity metrics (DSC, HD and VD) were computed for all structures, showing high agreement for well-defined anatomical structures such as the globe (DSC = 0.92, HD = 0.10) and extraocular muscles (DSC = 0.56–0.84), and low to moderate agreement for more anatomically variable regions such as extraconal fat.

Edit: **Revised (p. 10, ll. 466–476):**

Although the atlas labels were derived from automated segmentations, the aggregation of a large cohort through majority voting is expected to reduce the impact of isolated segmentation errors. The additional manual atlas validation further supports the anatomical plausibility and overall consistency of the resulting atlas representation. Importantly, the similarity metrics extracted from that validation are not directly comparable to the subject-level nnU-Net evaluation (N=43), as the atlas manual segmentation follows a slightly different annotation protocol. In particular, the manual atlas included a more extended optic nerve, a larger and

Table 5: S9 Table. Similarity metrics between manual atlas segmentation and automatically generated atlas labels. DSC: Dice Similarity Coefficient, HD: average Hausdorff distance, VD: volume difference. Differences reflect variations in annotation protocol between atlas and subject-level manual segmentations.

**Structure**	**DSC**	**HD**	**VD**
Average	0.69	0.53	0.47
Lens	0.52	0.38	0.95
Globe	0.92	0.11	0.13
Optic nerve	0.74	0.80	0.36
Intraconal fat	0.75	0.39	0.32
Extraconal fat	0.34	1.73	1.27
Lateral rectus	0.57	0.56	0.83
Medial rectus	0.76	0.31	0.31
Inferior rectus	0.84	0.17	0.16
Superior rectus	0.79	0.32	-0.07

fully connected extraconal fat compartment, and an anterior globe region surrounding the lens, leading to systematic volumetric differences and reduced overlap metrics. Despite these differences, the observed spatial agreement remains consistent with expected anatomical variability, supporting the validity and anatomical coherence of the atlas labels derived from large-cohort aggregation. To promote transparency and reproducibility, the manual atlas segmentation is now provided in the supplementary materials and will be made publicly available through the existing dataset on Zenodo [61].

Edit: **Revised (p. 14, ll. 687–696):**

To generate the atlas labels, we first registered each subject to the corresponding atlas space (male or female) and projected the nnU-Net segmentations accordingly. The overall process required approximately 25 minutes for the male cohort and 39 minutes for the female cohort. We then constructed a maximum-probability atlas using majority voting across subjects. In addition, we explicitly represented uncertainty in the atlas labels through probability maps, which we provide alongside the atlas. These maps encode the voxel-wise frequency of label occurrence across the cohort after spatial normalization, thereby reflecting the degree of consensus (or uncertainty) for each anatomical structure. This probabilistic representation complements the maximum-probability labels and allows identification of regions with higher uncertainty, such as diffuse anatomical boundaries (e.g., extraconal fat). For visualization purposes, we color-coded the probability maps by modulating label-specific intensities according to voxel-wise probabilities, such that lower-probability regions appear less saturated. The eye atlases can be downloaded at [61].

Edit: **Revised (p. 16, ll. 704–706):**

> To evaluate the reliability of the atlas labels, the combined atlas was manually reviewed and segmented by one of our ophthalmologist coauthors (AKL). Similarity metrics between the manual atlas segmentation and the automatically generated atlas labels were computed for each structure using DSC, HD and VD.

Comment #14:

5. Axial Length Estimation Shows Systematic Bias. The manuscript uses an MRI-specific definition of AL (posterior corneal surface to posterior globe boundary with orbital fat) rather than standard clinical optical biometry AL, but does not discuss implications for clinical interpretability or comparability across modalities/devices.

AR: We thank the reviewer for this important comment. We agree that MRI-derived axial length (AL) requires clarification regarding clinical interpretation. We have revised the manuscript to state that MRI-based AL should be considered an anatomical proxy rather than a replacement for optical biometry, which remains the clinical gold standard. We also clarify that our definition (posterior cornea to posterior globe boundary) follows established imaging conventions, where corneal and retinal thicknesses approximately cancel each other out. The Discussion now highlights that MRI-based AL is particularly useful in scenarios where optical methods are limited (e.g., dense cataracts, silicone oil, highly myopic eyes) and provides a reproducible measurement that can be compared across cross-sectional imaging modalities. These points are now explicitly addressed in the revised manuscript.

Edit: **Revised (p. 9, ll. 380–393):**

> MRI-based AL measurements are intended as a robust anatomical proxy of partial coherence interferometry (optical biometry), and not a replacement. While optical methods are the clinical gold standard for refractive measurements, MRI-based AL provides comparable accuracy (millimeter versus micro-meter precision) in cases with dense cataract changes and silicone oil filling the vitreous cavity [85], in highly myopic patients [86], or for research purposes in studies that cannot rely on biometry to study neurodegenerative changes and to control for differing eye size across individuals [43]. It may also provide valuable information in specific clinical scenarios, such as patients with rhegmatogenous retinal detachments with macular detachment or vitreous and submacular hemorrhage, where preoperative axial length is often underestimated [87]. Methodologically, our definition (posterior cornea to posterior globe boundary) follows established imaging standards where the corneal and retinal thicknesses approximately cancel each other out, minimizing the net error in clinical MRI sequences [85]. Unlike optical biometry, which can be affected by opacities or reflections in staphylomatous eyes, MRI offers an investigator-independent, reproducible measurement that is directly transferable to other cross-sectional modalities like CT or ultrasound. For clinical applications requiring the fusion of different modalities, previous studies have already demonstrated the feasibility of registering MRI datasets with ultrasound or biometric data to ensure comparability [88].

Comment #15:

6. BMI and Sex-Difference Analyses Are Statistically Flawed. The BMI–volume analysis uses simple Huber regression without adjusting for age, height, or head size, all established confounders. No p-values for regression coefficients or power analyses are reported. Similarly, sex differences in volumetry (Table 3) are claimed without any formal statistical test, effect sizes, or adjustment for body size. These analyses would not pass basic statistical review.

AR: We thank the reviewer for this important comment. Following this suggestion, we extended the BMI–volume analysis by incorporating additional covariates, including age and body height, and performing adjusted regression models separately for males and females. These analyses confirm that BMI remains independently associated with most orbital structure volumes, although with modest effect sizes, indicating limited explanatory power. We also performed formal comparisons between males and females, including both unadjusted and adjusted analyses. Several differences remained significant after adjustment, while others were attenuated, suggesting that part of the observed sex differences is explained by anthropometric variability. We explored including head size as a covariate; however, due to variability in MRI-derived head masks and its indirect relation to orbital capacity, it was not retained in the final models and is acknowledged as a limitation. All corresponding statistical results are now reported in the manuscript and supplementary materials.

Edit: **Revised (p. 5, ll. 188–195):**

> In terms of volumetry extraction, we provide the first large-scale benchmark MR-Eye volumetry of all eye structures. Descriptive statistics, including mean ± standard deviation, median with interquartile range, coefficients of variation (CV%), and 95% confidence intervals, are reported in Table 3. Unadjusted analyses showed significantly larger volumes in males compared to females for all structures except the lens (all FDR-corrected $p < 0.05$), with small-to-moderate effect sizes (see S3 Table). After adjustment for body height (and age), several sex differences remained significant, particularly for intra- and extraconal fat and the globe, whereas others were attenuated, indicating that part of the observed differences is explained by anthropometric variability (see S4 Table). Fig. 4 illustrates the distribution of volumetric measurements per structure, grouped by sex, using violin plots from the large-scale cohort of 1,157 subjects.

Edit: **Revised (p. 5, ll. 197–205):**

> Interestingly, robust linear regression (Huber) showed low coefficients of determination ($R^2 < 0.1$ in most cases), indicating limited explanatory power of BMI. Fig. 5 illustrates these relationships with scatter plots and Huber regression lines, grouped by sex. To account for potential confounding factors, we further performed adjusted robust regression analyses including age and body height as covariates. BMI remained significantly associated with most orbital structure volumes in both sexes (all FDR-corrected $p < 0.05$, except for lens volume in males), although effect sizes remained modest (see S5 Table). Consistent with these findings, Pearson correlation analysis revealed small to moderate effect sizes ($r = 0.11$–0.38, all $p < 0.001$ after Benjamini–Hochberg correction), corresponding to low explained variance ($R^2 < 0.15$ across all structures, see S6 Table). These results indicate that BMI has an independent but limited contribution to the variability of orbital tissue volumes.

Edit: **Revised (p. 10, ll. 415–431):**

Interestingly, although previous work [42] reported a significant association between exophthalmometry (defined as the perpendicular distance between the interzygomatic line and the posterior surface of the cornea) and both axial length and BMI ($p < 0.001$), our investigation revealed only weak—albeit statistically significant—associations between BMI and eye structure volumes. This suggests that BMI explains only a limited proportion of the variability in orbital tissue volumes. One possible explanation for this discrepancy lies in the different phenotypes being measured. Exophthalmometry quantifies the anterior displacement of the globe relative to the lateral orbital rim and is therefore strongly influenced by the capacity and geometry of the bony orbit. In contrast, our MRI-based volumetric analysis directly measures intraorbital soft tissues. As a result, increases in orbital fat volume (e.g., Grave's disease) may be partially accommodated by orbital architecture and not necessarily translate into measurable proptosis. By focusing on tissue volume rather than globe position, our approach captures a distinct anatomical phenotype, which may explain the weaker associations observed. We further accounted for potential confounding factors by including age and body height in adjusted regression models, which confirmed that BMI has an independent but modest association with orbital volumes. Although head or orbital size could also influence these relationships, we did not include a head-size covariate in the final models. Automated MRI-derived head masks showed variability across subjects (e.g., due to differences in field-of-view and head segmentation consistency), limiting their reliability as a covariate. Moreover, global head size represents only an indirect proxy for orbital capacity. Future work incorporating direct measures of orbital or craniofacial anatomy may help further clarify these relationships.

Table 6: S3 Table. Unadjusted comparisons between males and females for orbital structure volumes. Mean difference is reported as male minus female.

Structure	Mean diff (mm^3)	Cohen's d	p (Welch)	p (FDR)
Lens	0.44	0.01	0.820	0.820
Globe	198.57	0.22	$2.50 \cdot 10^{-4}$	$2.81 \cdot 10^{-4}$
Optic nerve	34.44	0.29	$8.36 \cdot 10^{-7}$	$1.08 \cdot 10^{-6}$
Intraconal fat	493.11	0.67	$2.40 \cdot 10^{-28}$	$1.08 \cdot 10^{-27}$
Extraconal fat	1063.84	1.11	$4.21 \cdot 10^{-67}$	$3.79 \cdot 10^{-66}$
Lateral rectus	50.67	0.48	$1.05 \cdot 10^{-15}$	$1.89 \cdot 10^{-15}$
Medial rectus	39.38	0.32	$1.19 \cdot 10^{-7}$	$1.79 \cdot 10^{-7}$
Inferior rectus	62.31	0.52	$6.92 \cdot 10^{-18}$	$1.56 \cdot 10^{-17}$
Superior rectus	117.29	0.52	$5.81 \cdot 10^{-18}$	$1.56 \cdot 10^{-17}$

Table 7: S4 Table. Adjusted regression analysis of sex differences in orbital volumes, including body height and age as covariates. Sex coefficient (β_{sex}) represents the effect of male relative to female.

Structure	β_{sex} (mm^3)	SE	p	p (FDR)
Lens	-4.10	2.70	0.129	0.145
Globe	-196.39	71.63	0.006	0.011
Optic nerve	-19.80	9.60	0.039	0.051
Intraconal fat	337.83	60.88	$3.56 \cdot 10^{-8}$	$1.60 \cdot 10^{-7}$
Extraconal fat	752.31	75.76	$2.35 \cdot 10^{-22}$	$2.12 \cdot 10^{-21}$
Lateral rectus	21.74	8.73	0.013	0.019
Medial rectus	4.81	10.23	0.638	0.638
Inferior rectus	27.72	9.47	0.003	0.008
Superior rectus	67.31	18.83	$3.64 \cdot 10^{-4}$	0.001

Table 8: S5 Table. Adjusted robust regression results for BMI and orbital volumes (males).

Structure	N	β_{BMI}	SE	p_{FDR}
Lens	568	0.12	0.25	0.65
Globe	568	27.18	7.02	1.39×10^{-4}
Optic nerve	568	8.86	1.31	2.34×10^{-11}
Intraconal fat	568	47.46	6.86	1.03×10^{-11}
Extraconal fat	568	35.33	9.69	2.98×10^{-4}
Lateral rectus muscle	568	8.66	1.02	1.27×10^{-16}
Medial rectus muscle	568	8.86	1.23	1.70×10^{-12}
Inferior rectus muscle	568	5.24	1.19	1.53×10^{-5}
Superior rectus muscle	568	17.70	2.09	1.27×10^{-16}
