# Supplementary material for: A-eye: Automated 3D MRI segmentation and morphometric feature extraction for eye and orbit atlas construction
Source: PLoS One. 2026 Jul 2;21(7):e0352257. doi: 10.1371/journal.pone.0352257 (PMC13327317; doi:10.1371/journal.pone.0352257)
Supplement: Attachment — Submitted filename: Response_to_Reviewers_auresp_2.pdf [file pone.0352257.s016.pdf]

Authors’ Response to Reviews of

J. Barranco et al. – “A-eye: Automated 3D MRI Segmentation and Morphometric Feature Extraction for Eye and Orbit Atlas Construction”

AR: Authors’ Response

Comment #1:

Comment – 9: Check with language and grammar. For me I am happy with the response given by the authors and I don’t have much comment to state as I am satisfied with the authors’ response.

AR: We thank the reviewer for the positive feedback. We have proofread the manuscript and corrected grammatical and typographical errors throughout.

Journal Requirements

AR: We thank the Editor for these requirements and confirm that both points have been addressed, as described below.

Reviewer-recommended references. All references requested by the reviewers were reviewed for relevance and added to the manuscript.

Reference list integrity. We have reviewed the reference list and its formatting, and confirm that none of the cited works has been retracted. All references were managed, checked, and inserted using the Zotero plugin for Microsoft Word. For references without a DOI, the available URL was added instead.
