# Supplementary material for: A-eye: Automated 3D MRI segmentation and morphometric feature extraction for eye and orbit atlas construction
Source: PLoS One. 2026 Jul 2;21(7):e0352257. doi: 10.1371/journal.pone.0352257 (PMC13327317; doi:10.1371/journal.pone.0352257)
Supplement: Attachment — Submitted filename: PONE-D-25-59562.docx [file pone.0352257.s013.docx]

**Study title:** A-eye: Automated 3D MRI Segmentation and Morphometric Feature Extraction for Eye and Orbit Atlas Construction

Dear Authors,

Thank you for this submission to PLOS ONE,

The study demonstrates strong novelty and potential impact, particularly as the first large-scale eye atlas with comprehensive multi-structure segmentation and sex-stratified analysis. The authors are commended for their transparent data practices, extensive supplementary materials, clear metadata, and candid acknowledgement of limitations. The statistical analyses are generally appropriate, employing multiple complementary metrics (DSC, HD, VD) and reporting confidence intervals, which strengthens the credibility of the findings.

However, several points require clarification or further evidence to fully support the conclusions. I request the authors’ input on the following issues, which I believe should be addressed to improve the manuscript’s rigour and interpretability:

**Comment – 1 (Lines 252–253):** The dataset includes only manually annotated right eyes, yet the atlas and volumetric benchmarks are applied to the full 1,157-subject cohort without validating generalisation to left eyes. While the authors propose an inversion of the left–right axis to extend the model to the left eye, no empirical evidence is provided to support this approach. Because orbital anatomy exhibits documented left–right asymmetry, with left orbits often 2–8% larger in mean volume, training exclusively on right eyes may introduce systematic bias, especially for fine structures such as the rectus muscles and intraconal fat boundaries.

To address this, the authors should include a brief validation on left eyes in the current revision by manually annotating a small sample (approximately 5–10 left eyes) and evaluating performance using standard metrics such as DSC, Hausdorff distance, and volume difference. This step is essential to confirm the robustness and applicability of the atlas and volumetric benchmarks to both eyes.

**Comment – 2 (Lines 269–274):** The authors correctly highlight the limitations of generic brain-focused MRI-QC tools for eye-specific imaging, which is an important observation. However, the proposed QC protocol lacks formal quantitative validation and inter-rater reliability assessment. The custom 0–4 quality rating scale does not define objective criteria for each level (e.g., measurable noise thresholds, blur quantification, or motion artifact detection), and no inter-rater agreement statistics (Cohen’s kappa or ICC) are reported for the two raters mentioned. Additionally, the relationship between QC scores and segmentation performance has not been fully validated across the entire cohort; the correlation observed for 43 test images may not generalise. Addressing these points would strengthen the reliability and applicability of the proposed QC framework.

**Comment – 3 (Lines 174–182, 280–287):** Axial length (AL) extraction failed in 2% of cases (28/1,157), primarily due to lens segmentation errors, but the manuscript does not systematically characterise these failures or assess performance in pathological eyes. While Figure 3A shows moderate-to-high correlation with manual references on 43 subjects, this limited analysis does not provide full insight into reliability across the cohort.

The authors should report sensitivity and specificity for lens detection from T1-weighted images and examine the relationship between image quality scores and segmentation success. Additionally, performance in pathological eyes (e.g., cataracts, post-surgical changes, lens dislocation) should be discussed, as these conditions may reduce contrast and affect segmentation. The relatively high rate of absent cornea detection (17% of eyes imaged open) also indicates sensitivity to anatomical variability and warrants further explanation. Addressing these points would strengthen confidence in the AL extraction pipeline.

**Comment – 4 (Lines 188–191, 295–302):** The study reports minimal correlation between BMI and eye structure volumes (R² < 0.1), which contrasts with prior findings linking BMI to exophthalmometry (Reference [42], p < 0.001). The explanation invoking head size or body height is speculative, as these variables were not measured in the current cohort.

For transparency, the authors should report the correlation coefficients (e.g., Pearson's r) and their associated p-values. If head size or body height are suggested as confounders, they should be clearly framed as hypotheses rather than definitive explanations. Additionally, the manuscript should discuss the phenotypic differences between volumetric measurements and exophthalmometry to contextualise the discrepancy with prior studies. Addressing these points would improve interpretability and strengthen the conclusions regarding BMI-related effects.

**Comment – 5 (Lines 311–314):** The authors acknowledge that the cohort is predominantly ethnically homogeneous, but their discussion of cross-ethnic comparability relies on general statements rather than sufficient evidence. The claim that key ocular anatomical features are largely comparable across ethnicities, with only minor differences, requires stronger support.

Specifically, the manuscript does not report the ethnic or racial composition of the SHIP cohort, nor does it critically evaluate the cited reference [85]. Moreover, recent ophthalmologic studies document clinically significant ethnic differences in axial length, orbital volume, and myopia prevalence, which may affect the generalizability of the atlas. The authors should provide clearer demographic reporting, evaluate relevant literature, and explicitly discuss potential limitations arising from ethnic homogeneity.

**Comment – 6 (Lines 391–399):** While the manuscript provides extensive hyperparameter details, it lacks justification for architecture-specific choices, particularly the patch size [128, 160, 112], and does not include ablation studies to support these decisions. Using default nnU-Net parameters with minimal modification leaves critical questions unanswered.

Specifically, the authors should clarify why this patch size was selected and whether performance is sensitive to different patch sizes (typical ranges: [64, 64, 64] to [256, 256, 256]). The choice of five-fold cross-validation should also be justified, as a higher fold number (e.g., 10-fold) could better estimate variance. Additionally, the suitability of the data augmentation strategies (scaling, rotation) for fine structures such as the optic nerve and rectus muscles should be discussed. Finally, including training curves (loss, DSC over epochs) would help assess potential overfitting and provide insight into model convergence.

**Comment – 7 (Lines 443–445):** Volumetry is reported at a voxel resolution of 1 mm³, but the manuscript does not provide measurement precision, confidence intervals, or test-retest reliability, limiting the assessment of clinical utility. While Table 3 presents median ± standard deviation, important metrics are missing, including coefficient of variation (CV%), 95% confidence intervals, and repeatability data.

Additionally, clinically meaningful thresholds for orbital pathologies are not discussed. For example, extraconal fat volume shows high variability (±1,134 mm³ in males, ~31% CV), raising concerns about the reliability of these measurements for diagnostic or clinical purposes. Including these metrics would strengthen the manuscript by demonstrating both precision and potential clinical applicability of the volumetric data.

**Comment – 8 (Lines 247–250):** The study relies on a single manual segmentation per subject, preventing assessment of inter-rater reliability via Intraclass Correlation Coefficient (ICC), as performed in prior comparative studies (Reference [20]). This is a significant methodological limitation that affects the reliability of the “ground truth” annotations.

Without independent rater assessments, the annotations may reflect individual rater bias, particularly for structures with high anatomical variability, such as rectus muscles and orbital fat, where inter-rater disagreement is typically 5–15% in the literature. Consequently, it is difficult to determine whether the reported nnU-Net performance (DSC = 0.80 ± 0.07) represents optimal segmentation accuracy or merely falls within the expected range of human variability. Acknowledging and discussing this limitation is important for contextualising model performance.

As a reviewer, I would like to thank the journal editor for the opportunity to review this novel and impactful work. I appreciate the authors’ efforts in developing a comprehensive, large-scale eye atlas, and I hope my comments and suggestions will further strengthen the manuscript.
